# Global investigation of composition and interaction networks in gut microbiomes of individuals belonging to diverse geographies and age-groups

**DOI:** 10.1186/s13099-016-0099-z

**Published:** 2016-05-06

**Authors:** Deepak Yadav, Tarini Shankar Ghosh, Sharmila S. Mande

**Affiliations:** Bio-Sciences R&D Division, TCS Research, Tata Consultancy Services Ltd., 54-B, Hadapsar Industrial Estate, Pune, 411013 Maharashtra India

**Keywords:** Gut microbiome, Metagenomics, Interaction networks, Bioinformatics, Network analysis

## Abstract

**Background:**

Factors like ethnicity, diet and age of an individual have been hypothesized to play a role in determining the makeup of gut microbiome. In order to investigate the gut microbiome structure as well as the inter-microbial associations present therein, we have performed a comprehensive global comparative profiling of the structure (composition, relative heterogeneity and diversity) and the inter-microbial networks in the gut microbiomes of 399 individuals of eight different nationalities.

**Results:**

The study identified certain geography-specific trends with respect to composition, intra-group heterogeneity and diversity of the gut microbiomes. Interestingly, the gut microbial association/mutual-exlusion networks were observed to exhibit several cross-geography trends. It was seen that though the composition of gut microbiomes of the American and European individuals were similar, there were distinct patterns in their microbial interaction networks. Amongst European gut-microbiomes, the co-occurrence network obtained for the Danish population was observed to be most dense. Distinct patterns were also observed within Chinese, Japanese and Indian datasets. While performing an age-wise comparison, it was observed that the microbial interactions increased with the age of individuals. Furthermore, certain bacterial groups were identified to be present only in the older age groups.

**Conclusions:**

The trends observed in gut microbial networks could be due to the inherent differences in the diet of individuals belonging to different nationalities. For example, the higher number of microbial associations in the Danish population as compared to the Spanish population, may be attributed to the evenly distributed diet of the later. This is in line with previously reported findings which indicate an increase in functional interdependency of microbes in individuals with higher nutritional status. To summarise, the present study identifies geography and age specific patterns in the composition as well as microbial interactions in gut microbiomes.

**Electronic supplementary material:**

The online version of this article (doi:10.1186/s13099-016-0099-z) contains supplementary material, which is available to authorized users.

## Background

Microbial communities residing in different ecological niches are known to play several key functions and often define the phenotypic characteristics of their environments. A typical microbial community consists of numerous bacterial/archaeal species belonging to diverse taxonomic lineages. Several recent studies have indicated that the functional behaviour of a bacterial species is not only dictated by its own genomic content, but is also influenced by the presence of other microbes that co-habit in that given environment [1, 2]. In other words, the function of a given microbe in an environment is dependent on its interactions with other resident microbes present in that environment. Therefore, in order to obtain a holistic insight into the role of the microbial community in determining the phenotypic traits of an environment, it is necessary to understand the inter-microbial interaction patterns present within the environment under study.

A key focus of several concerted efforts by independent research groups as well as consortia like the human microbiome and the meta-HIT projects has been to profile as well as characterize the microbial communities residing in various body sites [3–5]. Various studies have also attempted to identify differences in microbial communities inhabiting different body sites of individuals from certain geographies and age-groups [6–14]. However, a comprehensive analysis across geographies as well as different age groups is still non-existent.

Cohabiting microbes in an environment can interact with each other in various ways. For example, they may have positive interactions like mutualism and commensalism, or negative interactions like parasitism, amensalism and competition. A few recent studies have attempted to infer such inter-microbial co-occurrence/exclusion networks across different environments [15–17]. For example, a study on metagenomic datasets from 18 different human body sites, obtained from 239 individuals, has identified a global network of 3005 significant (positive and negative) interactions across 197 bacterial groups [1].

Most of the reported studies have inferred the inter-microbial interactions based on the co-occurrence patterns of various microbes across samples [1, 15–17]. In other words, a pair of bacterial/archaeal species was considered to ‘interact’ if their abundance profiles exhibited co-occurrence or mutual exclusion across multiple samples. Since the relationships between different microorganisms are predicted based on the similarity/dissimilarity in their abundance patterns in various samples, a correlation-based analysis is a key step in inferring the microbial association networks (in a given environment). While the edges in the co-occurrence network indicate positively correlated species, they depict negatively correlated species in the mutually exclusive network.

The co-occurrence as well as mutual exclusion networks can be utilized to investigate whether changes (or aberrations) in these networks can be associated with any disease or physiological disorder. The gut harbours one of the largest microbial communities in the human body. This microbial community is sensitive to environmental factors like diet, antibiotics as well as exposure to pathogens [18–21]. A recent study indicated variations in the gut microbial co-occurrence networks of individuals with varying nutritional status [22]. However, a comprehensive comparison of the gut microbial interaction networks from individuals belonging to diverse geographical locations is unavailable till date.

The motivation of obtaining and investigating a global (cross-geographic and cross-age-group) picture of the gut microbial communities, both in terms of their taxonomic composition as well as the inherent inter-microbial interaction networks therein, forms the basis of the current study. In this study, we have performed a comparative investigation of the gut microbial communities based on the available gut microbiomes from 399 individuals of various age groups and belonging to eight different nationalities. We have also performed an association analysis of the dietary consumption profiles of these nationalities with the composition of the gut microbiomes of the studied subjects, as well as the architecture of the inherent interaction networks.

## Results

### Core group of genera across gut microbiomes

The pattern of occurrence of each genus across gut microbiomes of 399 individuals from eight nationalities and various age-groups (Table 1) was profiled and relative homogeneity of the gut microbiomes was computed (described in the “Methods” section). While a total of 342 genera were observed to be present in at least one of the 399 gut microbiomes, 75 (17.1 %) and 267 (68 %) were observed to be detected in at least 90 and 50 % of the gut microbiomes, respectively. In other words, although the studied gut microbiomes were obtained from individuals from eight different nationalities and ranged in age from infancy till late 70s, a core group of 267 genera was found to occur across more than 50 % of the gut microbiomes. This indicates that there exists an inherent signature of taxonomic composition in the gut microbiome that is conserved across age and nationality of the individual.

**Table 1:** Distribution of number of individuals in each age group, considering all across nationalities (except America for which metadata was not available)

Group	Age group (years)	Number of individuals
G1	0–10	26
G2	10–30	39
G3	30–40	47
G4	40–50	69
G5	50–60	61
G6	60 and above	64

### Microbial community composition and their interaction patterns in gut microbiomes of individuals from different nationalities

#### Geography-specific trends in the gut microbial community structure of individuals from various geographies

**Fig. 1:**
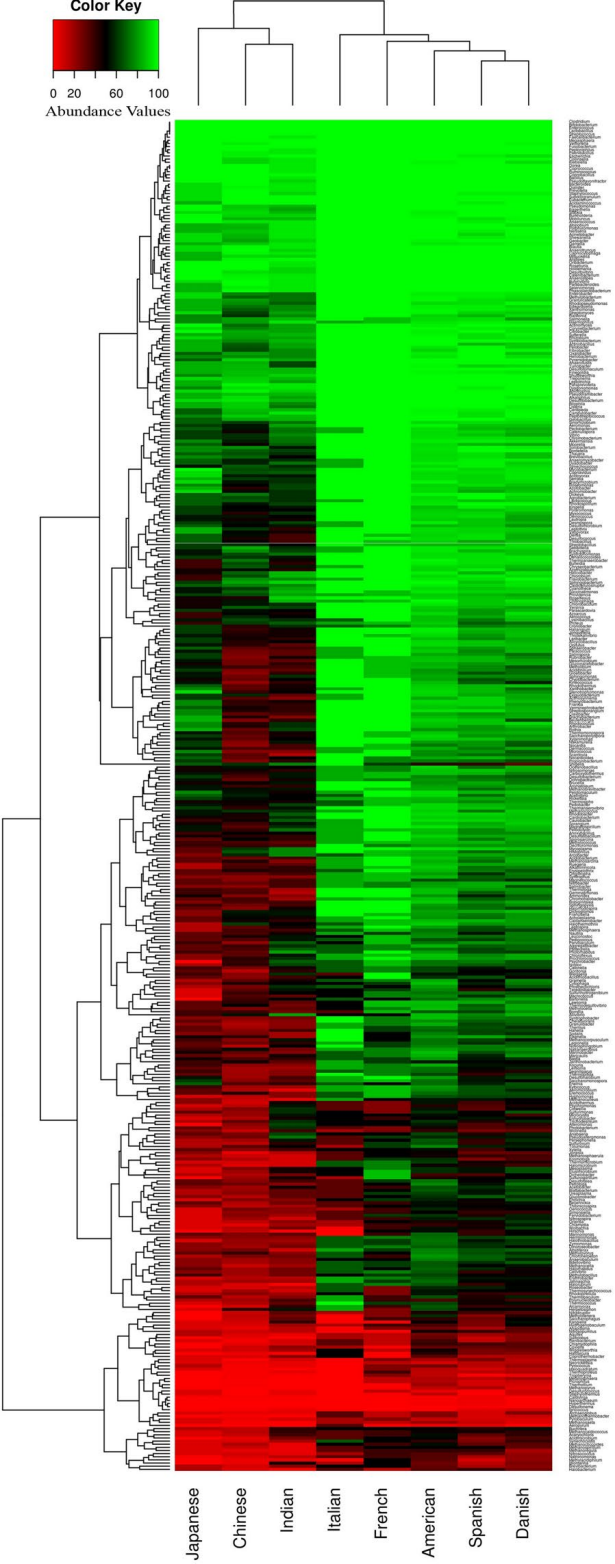
Heatmap showing the detection pattern of various genera in the gut microbiomes of individuals belonging to various nationalities. Two distinct clusters have been identified. The first group contains all the European nationalities along with the American samples. The second group contains the gut microbiomes of the Asian populations (Chinese, Japanese and Indians). *Red color* signifies that the genus is either absent or present in low abundance, whereas the *green color* signifies that it is highly abundant

In spite of the presence of a conserved group of genera across guts of individuals, a clear geography-specific signature of microbial composition was observed in the detected pattern of various genera (Fig. 1). The gut microbiomes from eight nationalities were found to form two distinct clusters. While the first group consisted of individuals belonging to the European and American nationalities, the second group consisted of the Asian populations. As compared to the Asian gut microbiomes (cluster 2), a higher number of genera were found to be associated with the European/American gut microbiomes (cluster 1). These results suggest that there exist geography-specific signatures in the gut microbiomes of the individuals.

One of the distinguishing factors observed in the gut microbiomes belonging to these clusters was the noticeably higher Jaccard distances (intra-nation) within the gut microbiomes of the Asian nationalities, as compared to those from America/Europe (Additional file 1). In other words, Asian populations were found to have much higher inter-individual heterogeneity in the composition of gut microbial community, as compared to the American/European individuals.

#### Overall properties of gut microbial interaction networks across different geographies

The microbial composition and structural properties of the inter-microbial interaction networks (both co-occurrence and mutual exclusion) were investigated in order to probe whether the observed trends in the overall composition of the gut microbiomes was also reflected at the level of microbial interactions. A key objective was also to investigate whether microbial genera dominant in a given microbiome had any influence on the interaction patterns present therein. The network properties of the gut bacterial communities across geographies are provided in Table 2.

**Table 2:** Overall properties of (A) co-occurrence network and (B) mutual exclusion network of gut microbiomes across all geographies

Regions	Number of vertices	Number of edges	Average degree	Diameter	Avg shortest path length	Network density	Clustering coefficient	Network centralization
*A*								
America	74	866	23.09	7	2.14	0.32	0.69	0.29
China	54	343	12.47	6	2.26	0.24	0.59	0.32
Denmark	70	446	12.56	10	2.71	0.18	0.66	0.29
France	76	99	2.57	9	3.52	0.03	0.51	0.10
India	64	216	6.65	10	3.64	0.11	0.58	0.18
Italy	55	81	2.89	1	1.00	0.05	1.00	0.08
Japan	84	455	10.71	8	2.72	0.13	0.54	0.22
Spain	68	160	4.64	8	3.52	0.07	0.51	0.13
*B*								
America	65	86	2.61	7	2.52	0.04	0	0.70
China	45	70	3.04	6	2.84	0.07	0	0.47
Denmark	49	57	2.28	8	3.17	0.05	0	0.34
France	57	48	1.66	7	3.10	0.03	0	0.14
India	6	4	1.14	2	1.43	0.22	0	0.50
Italy	24	14	1.12	2	1.33	0.05	0	0.13
Japan	26	24	1.78	6	3.26	0.07	0	0.27
Spain	31	28	1.75	6	2.78	0.06	0	0.26

Interestingly, although the gut microbiomes of American and European individuals were observed to be similar in terms of the structure, composition and variability of the microbial communities (Fig. 1; Additional file 1), distinct features could be identified in the gut microbial interaction patterns within these nationalities. For example, gut microbiomes of the American individuals had a noticeably higher node degree (23.09) and network density (0.32) in their co-occurrence networks as compared to the European populations. This indicates that the degree of positive inter-dependence between the gut microbiota in the American population is probably much higher than in the European populations.

**Fig. 2:**
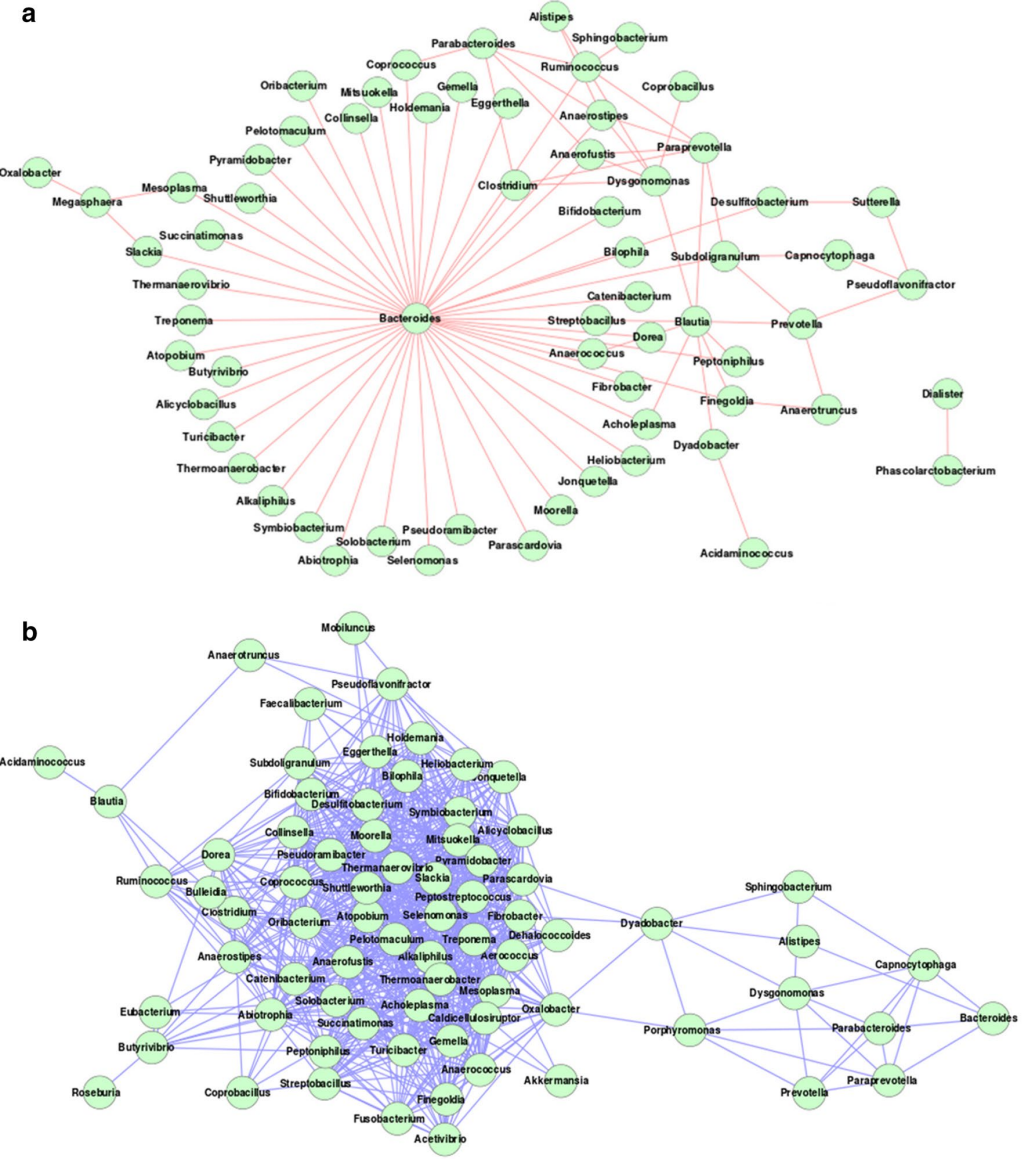
Gut microbial **a** mutual exclusion and **b** co-occurrence network observed in the gut microbiome of American individuals. The high network centralization property in the American individuals indicates a few central hub genera (Bacteroides) exhibiting mutual exclusion with other genera

An inspection of the gut microbiome mutual exclusion network for the American individuals indicated Bacteroides (with degree = 46) to have the highest exclusion tendencies amongst several other genera (Fig. 2a). This was followed by the genus Blautia which was observed to participate in nine exclusion interactions. Thus, there exist a few hub genera like Bacteroides which negatively modulate the occurrence of several other genera in the gut microbiomes of the American individuals. However, neither Bacteroides nor Blautia were observed to be significantly abundant in the American gut microbiomes as compared to other genera (Additional files 2 and 3). In contrast, significantly abundant genera (Allistipes, Sutterella and Akkermansia) in the American populations were not observed to play central role in the inter-microbial interaction networks (Additional file 4). The above result indicates that the key genera modulating the inter-microbial interaction networks may be different from the ones that are the most dominant in terms of composition/abundance.

**Fig. 3:**
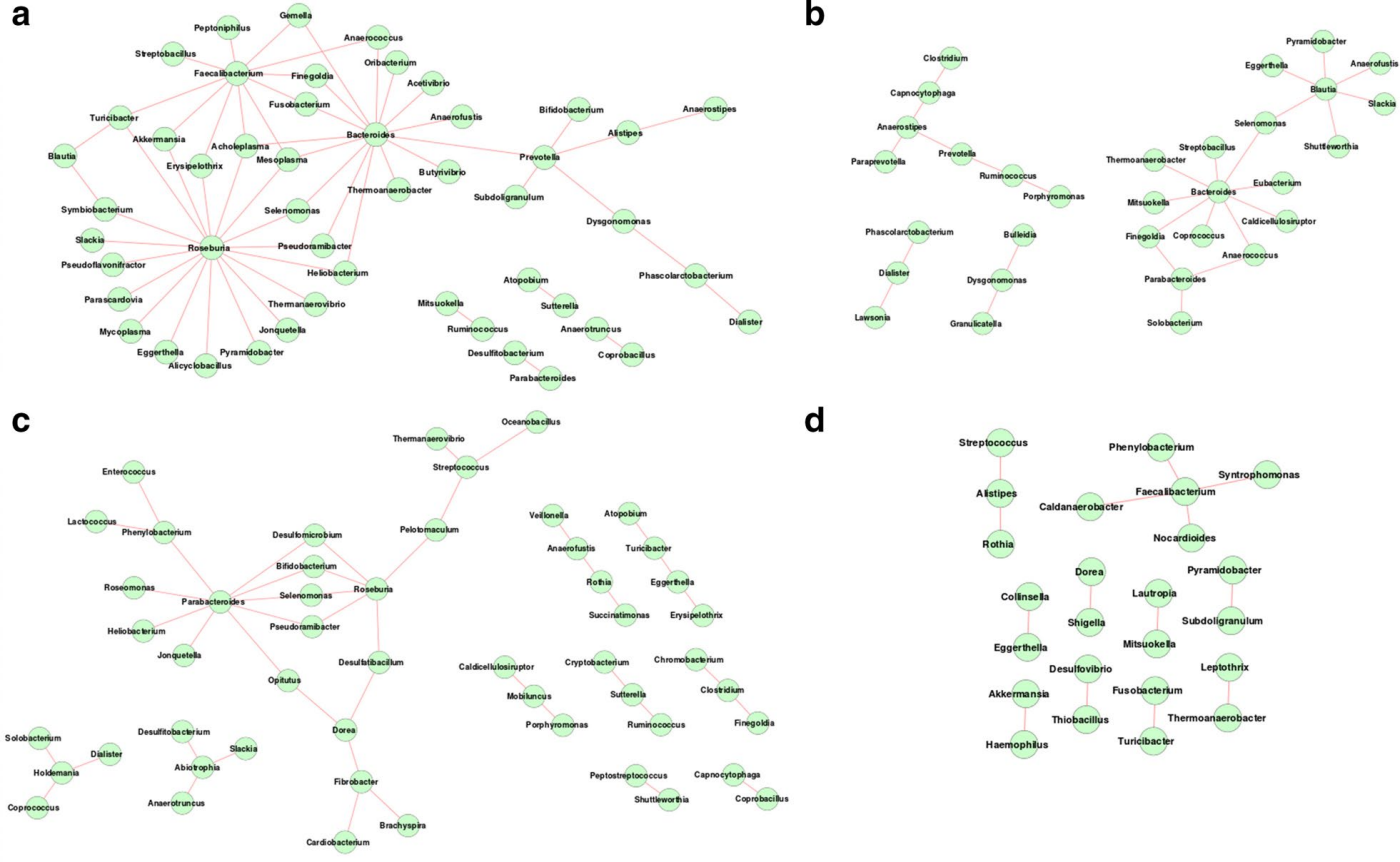
Gut microbial mutual exclusion network observed in individuals from **a** Denmark **b** Spain **c** France **d** Italy. Subtle differences in mutual exclusion patterns exist amongst different European populations

In spite of having a greater degree of homogeneity, with respect to gut microbial community structure and composition, distinct properties were observed amongst the gut microbiome interaction networks obtained for various nationalities within the European continent. Among the Europeans, the co-occurrence network obtained for the Danish population was observed to be the most dense, with an average degree of 12.56 (among the various genera). Although the co-occurrence network observed for the Spanish population was similar to that of the Danish individuals in terms of the number of nodes (70 for Danish and 68 for Spanish) (Table 2), the average degree of the nodes was observed to be noticeably lower (4.64). This probably indicates that in spite of the overall similarity in community composition and structure (characterized by dominance of Allistipes, Phascoloractobacterium, Roseburia, Akkermansia and Faecalibacterium) (Fig. 1; Additional files 1, 5, 6), there is an increased level of functional interdependence among the bacterial groups residing in the guts of the Danish individuals. For mutual exclusion networks, besides Bacteroides, the gut microbiota of the Danish individuals was observed to have genera Roseburia and Faecalibacterium showing multiple relationships (Fig. 3a). In Spanish population, Bacteroides and Blautia were observed to form hubs with Selemonas acting as a linker between these hubs (Fig. 3b). In contrast to the Danish and Spanish individuals, properties of the co-occurrence and mutual exclusion networks obtained for the Italian and French individuals were observed to be similar (Table 2). The similarity of the network properties in these two European countries could be a consequence of the fact that the average age of the individuals in these groups was high. However, this may be an artefact due to the lower number of samples constituting these cohorts (eight French; six Italian). The absence of larger number of publicly available metagenomes from these populations impeded us from further examining this aspect.

**Fig. 4:**
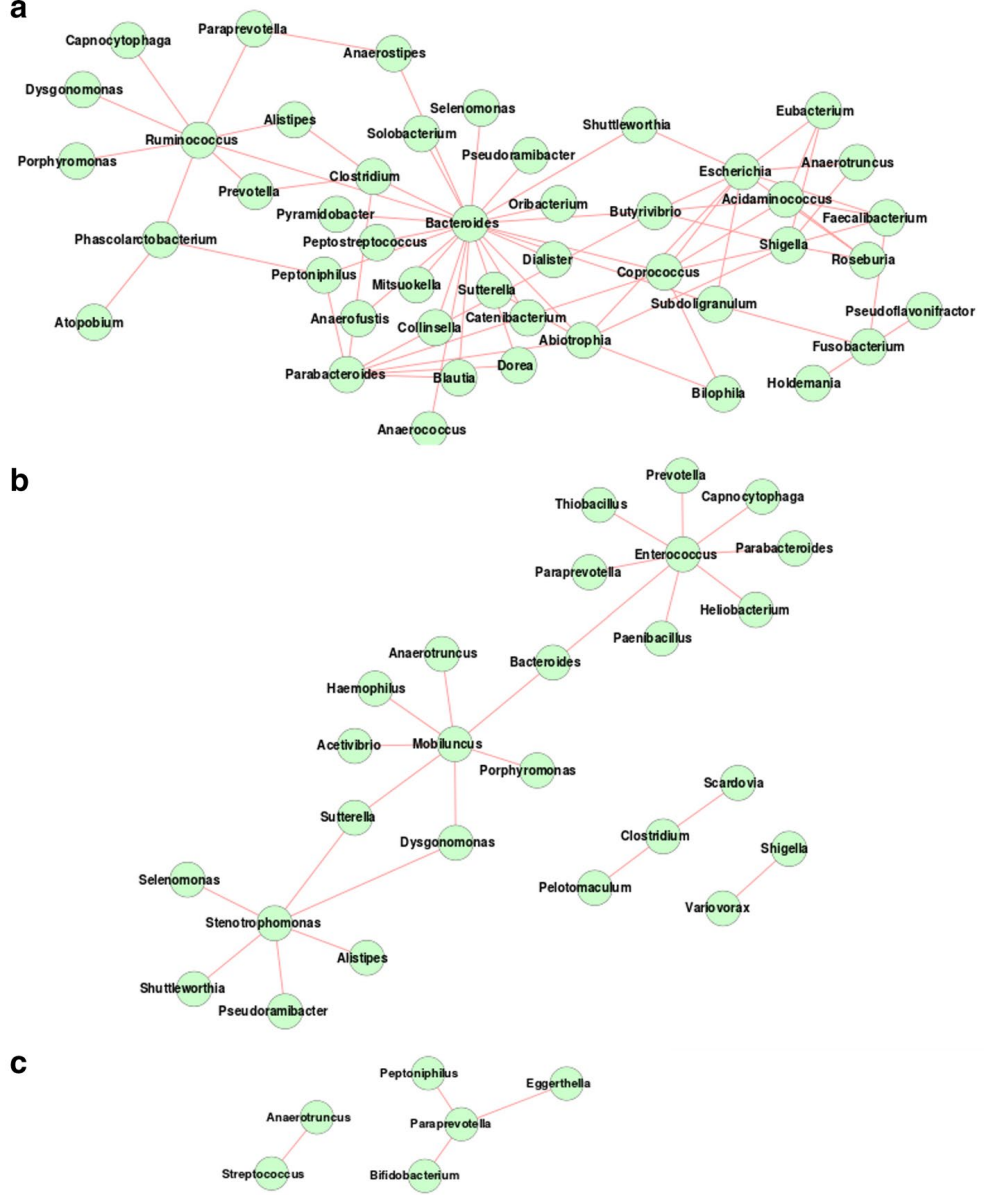
Mutual exclusion network in gut microbiomes of individuals belonging to **a** Chinese **b** Japanese and **c** Indian nationalities. Very few mutual exclusion interactions are observed for Indian, compared to other nationalities

In spite of having an overall similarity in the intra-individual variability (intra-nationality Jaccardian distances), complexity (Shannon diversity) and genera-based membership (presence/absence of different genera) of the microbial communities (Fig. 1; Additional file 1), the different nationalities within the Asian continent were observed to have clearly distinct features, not only with respect to the overall properties of the gut microbial interaction networks, but also with respect to the abundances of various microbial genera (Table 2; Additional files 7, 8, 9, 10). For example, while the overall properties of the interaction networks for the gut microbiota in Chinese and Japanese populations were observed to be similar, the co-occurrence network of the Japanese was observed to be larger in terms of the number of genera (84 in Japan compared to 54 in China). On the other hand, the density of the co-occurrence patterns (average degree of the nodes) was observed to be marginally higher in Chinese population (12.47 for China and 10.71 for Japan). However, as compared to the Japanese, the gut microbiota in Chinese were observed to have much stronger mutual exclusion patterns, not only with respect to the number of genera constituting the network (45 genera in China as compared to 26 in Japan), but also with respect to the average degree of the nodes (China 3.04, Japan 1.78). Similarly, while the genus Bacteroides was observed to have the maximum number of exclusion relationships in the Chinese population (as also observed for the American population), the gut microbial mutual exclusion network in the Japanese individuals was observed to have Enterococcus, Mobiluncus and Strentophomonas as the key hubs of negative associations (Fig. 4). Interestingly, with the exception of Bacteroides and Ruminococcus, none of the genera that were observed to be significantly over-abundant in the Chinese and Japanese populations acted as hubs in the inter-microbial networks. While the guts of the Chinese individuals were found to be characterized by a significant increase in the abundances of genera like Faecalibacterium, Bacteroides, Roseburia, Ruminococcus, and a significant lower abundances of Bifidobacterium, Sutterella, Akkermansia, Prevotella, Dialister, Collinsella, etc. (Additional files 7, 8), Bilophila was observed to be a signature genus in the gut microbiomes of the Japanese population (Additional files 9, 10).

In contrast to the Chinese and the Japanese populations, the gut microbiota of the Indian individuals were observed to be characterized by not only a distinct decrease in the average degree (6.65) of the genera comprising the co-occurrence network, but also lower Freeman network centralization (0.18) (Table 2). This probably indicates lesser functional interdependence as well as competition between the bacterial groups residing the guts of the Indian population. The mutual exclusion network for the Indian population was on the other hand, observed to be sparse with only six genera having four interactions between them.

The above results indicate that the groups of genera that tend to occur in majority in a given gut microbiome are distinct from the genera that play key role in modulating the inter-microbial interactions in a gut microbiome. In other words, specific group of genera act as hubs in the gut microbial networks.

#### Comparison of the compositions of bacterial interaction networks across nationalities

**Fig. 5:**
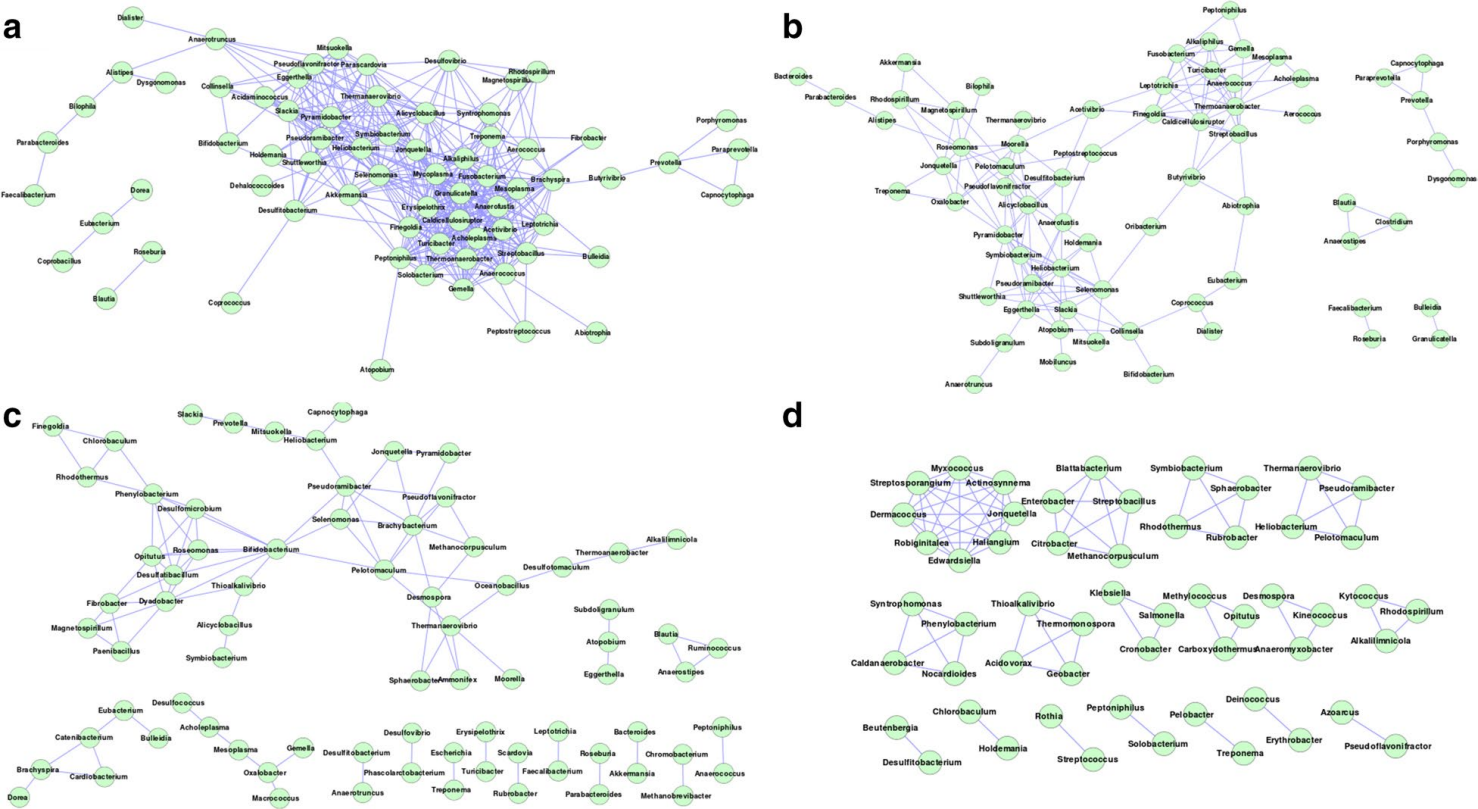
Gut microbial co-occurrence network observed for the **a** Danish **b** Spanish **c** French and **d** Italian nationalities. Distinct differences in the co-occurrence networks are seen in the gut microbiomes of individuals from various European nationalities

**Fig. 6:**
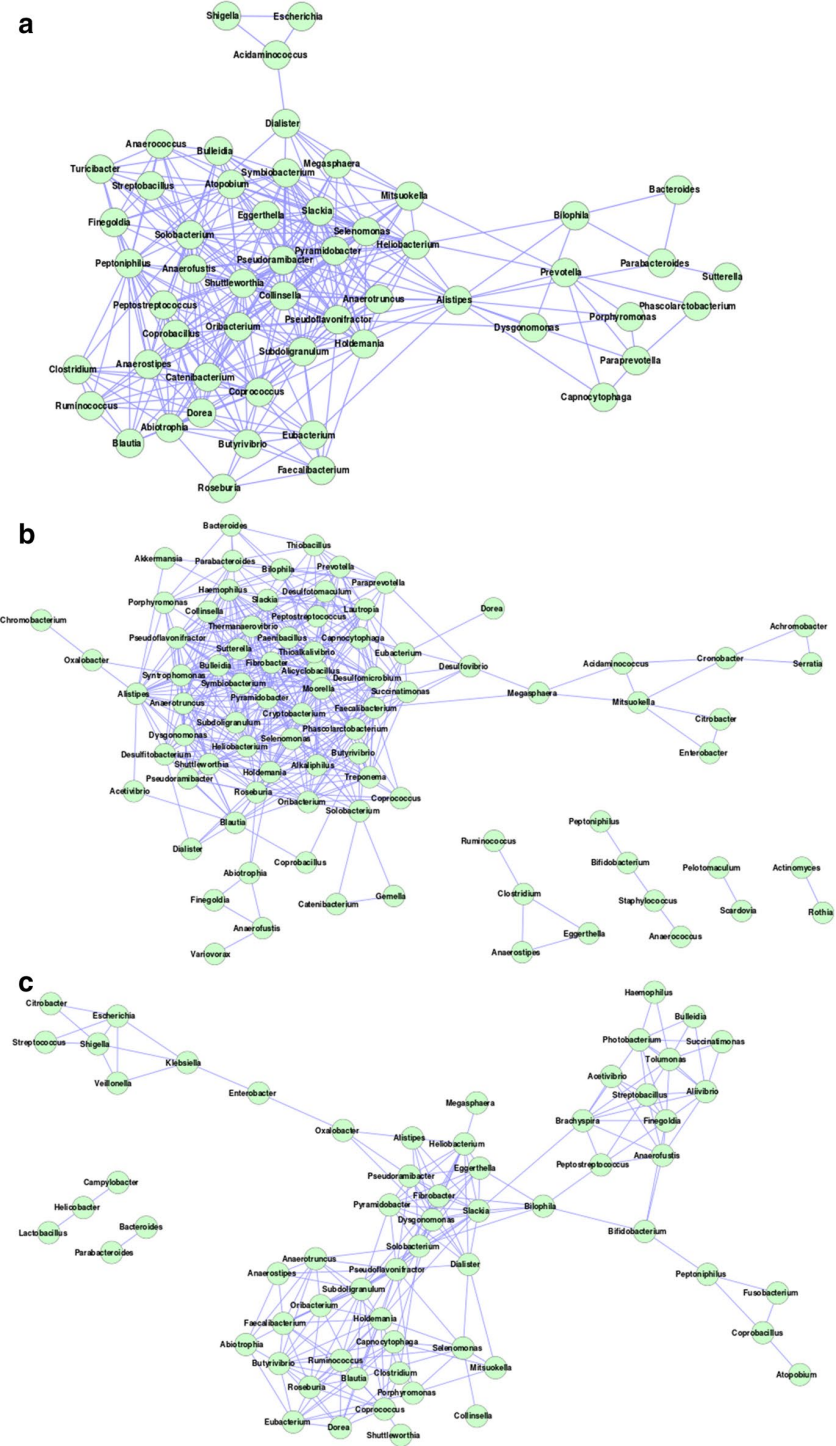
Gut microbial co-occurrence network observed for the **a** Chinese **b** Japanese and **c** Indian nationalities. Distinct differences exist in the co-occurrence networks of the gut microbiomes (specifically Indian v/s Chinese and Japanese)

The co-occurrence networks were observed to have similar architecture in the American, Danish, Chinese and Japanese guts. Each of these networks was characterized by a single major hub of genera, with auxiliary smaller hubs connected to it through one (or a few) connecting genera (Figs. 2b, 5, 6). On the other hand, Spanish, French and Indian populations were found to be similar, with multiple hubs of genera having several interconnections (Figs. 5b, c, 6c). This result suggests lesser functional interdependency among gut microbiota in these nationalities. Interestingly, the co-occurrence network of the Italian individuals was found to be distinct, with occurrence of multiple hubs having no interconnections between each other (Fig. 5d). The Indian individuals were found to have only one hub containing likely pathogenic genera (Escherichia, Shigella, Klebsiella, Streptococcus and Enterobacter). This group of genera were reported earlier to specifically co-occur with each other in the guts of the severely malnourished children [22]. The above results further confirm the presence of core group of pathogens that tend to co-occur in the guts of these children and probably have a key role for deciding nutritional status, as suggested by the original study [22].

The common interacting microbial pairs (i.e. edges) in the interaction networks were identified. Given the lower sample size, the French and Italian populations were removed from this study. Only three interactions were found to be common in all 6 geographic regions. In order to get a better overview of common interacting pairs (edges), the study was narrowed down to pair-wise comparison across specific regions (summarized in Table 3). Interestingly, the largest number (259) of common interacting pairs of taxa was observed between the gut microbiomes of American and Danish populations, followed by 223 between American and Chinese populations. However, only 92 interacting microbial pairs in the Chinese gut was found to be similar to those in the Danish gut. Similarly, gut microbiome networks of Chinese and Japanese individuals had fewer common interacting pairs (83) as compared to those between Chinese and American individuals (223), indicating a cross-continental trend. On the other hand, within the European nationalities, the microbiome interaction networks of Denmark and Spain were found to have 95 common edges. The above results are interesting as they indicate that, in spite of the geography-specific trends in the overall composition and structure of the gut microbial communities, cross-geographic trends exist in the gut microbiome association/exclusion networks.

**Table 3:** Overall network properties of common interacting microbes in gut microbiomes of individuals from various nationalities

Regions	Number of vertices	Number of edges	Average Degree	Diameter	Avg shortest path length	Network density	Clustering coefficient	Network centralization
America China	47	223	9.29	5	2.35	0.20	0.55	0.26
America Denmark Spain	37	85	4.47	7	3.03	0.12	0.60	0.19
America Denmark	53	259	9.59	5	2.13	0.18	0.68	0.28
China Denmark	36	92	4.97	7	2.71	0.14	0.69	0.21
China Japan India	22	24	2.09	6	2.65	0.10	0.19	0.20
China Japan	41	83	3.95	10	3.02	0.10	0.53	0.18
Denmark Spain	40	95	4.63	8	3.24	0.12	0.60	0.20

A deeper investigation of the common networks revealed that a few genera had maximum involvement in the common edges (Additional file 11). As observed earlier, Bacteroides was found to have highest (20) interactions in the mutual exclusion network. While genera Pyramidobacter, Pseudoflavonifractor, Shuttleworthia, Alistipes and Anaerostipes were found in the co-occurrence network in the guts, Bifidobacterium and Escherichia were observed to play roles in mutual exclusion network. In American and Chinese population, Pseudoramibacter was found to occur maximum times (degree = 20) in the common co-occurrence interactions, followed by Pyramidobacter and Collinsella (each having degree = 17). In the common gut microbial interaction networks of American and Danish individuals, while Selenomonas was observed to be an important hub with highest representation in the common edges in the co-occurrence network (betweeness = 1; degree = 23), Bacteroides was seen to be dominant in the mutual exclusion network. Between the gut microbial interaction networks of Chinese and Japanese populations, while Heliobacterium, Pyramidobacter, Anaerotruncus, Pseudoflavonifractor and Shuttleworthia were identified as nodes having high centrality values in the common co-occurrence network, none of the genera were found to be common in the mutual exclusion networks. While Thermoanaerobacter, Pyramidobacter and Turicibacter were observed to be frequent in co-occurrence networks in Spanish and Danish guts, Bacteroides was identified to be the key bacteria (participating in 4 out of 7 common interactions) in the mutual exclusion networks of Spain and Denmark. Pyramidobacter was observed to be present with high degree in most of the common co-occurrence networks. These results suggest that while genera like Pyramidobacter, Pseudoflavonifractor and Shuttleworthia have a general tendency to occur in the co-occurrence networks across various geographies, genera like Bacteroides have a tendency to negatively regulate the occurrence of many gut genera across individuals of different geographies. These results further indicate that gut microbial communities of individuals share specific common features (irrespective of their geographies). Similar analyses were also performed using order level taxonomic assignments for the studied metagenomic datasets, which indicated equivalent results (Additional file 12).

### Microbial community composition and their interaction patterns in gut microbiomes of individuals belonging to different age-groups

#### Age-specific trends in the gut microbial community structure

**Fig. 7:**
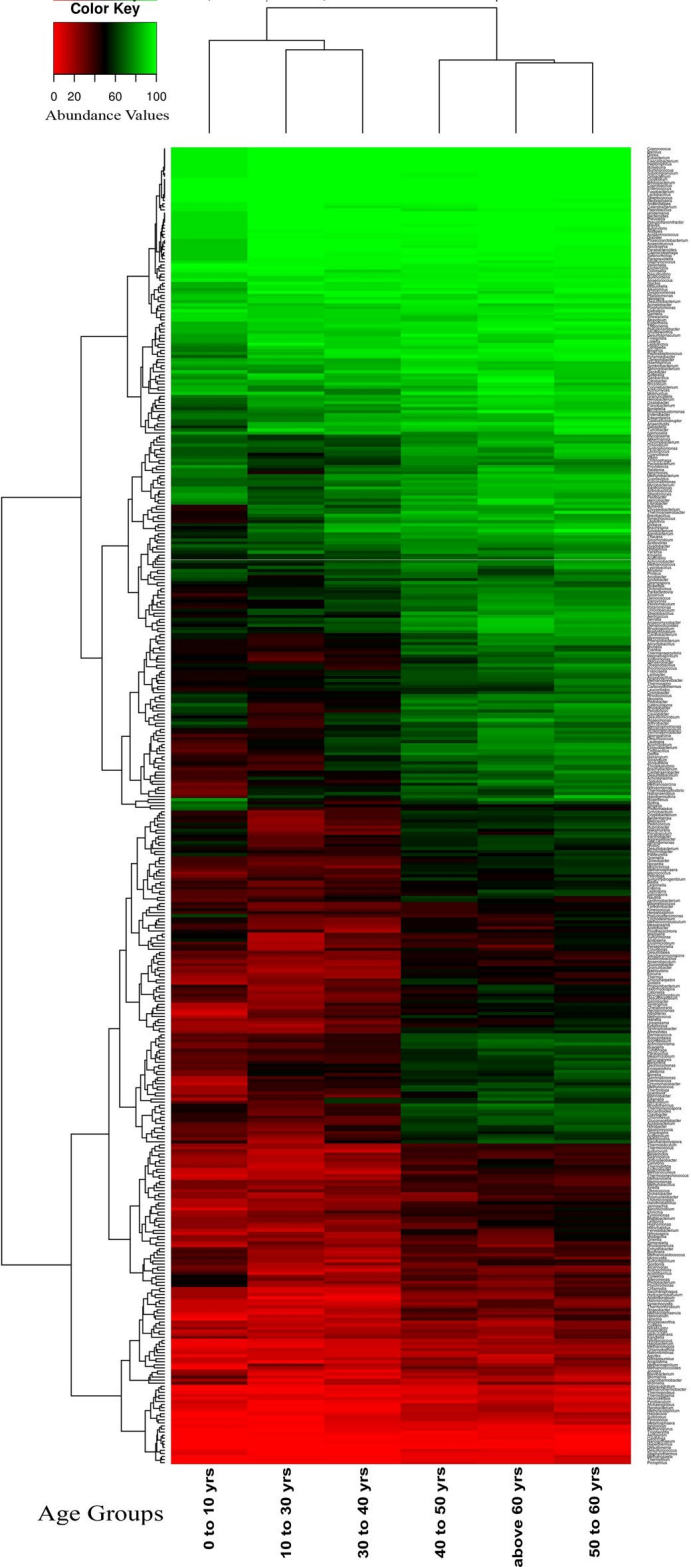
Heatmap showing patterns of various genera present in gut microbiome of individuals belonging to different age groups. In spite of being cross-geographic, the gut microbiomes of individuals belonging to the six different age groups cluster into two distinct groups. While the first group consists of the younger age groups (G1: 0–10 years, G2: 10–30 years and G3: 30–40 years), the second group consists of the older age groups (G4: 40–50 years, G5: 50–60 years and G6: 60 years and above). *Red color* signifies that the genus is either absent or present in low abundance, whereas the *green color* signifies that it is highly abundant

Two distinct clusters of gut microbes were observed in all the individuals, irrespective of the geographies to which they belong (Fig. 7). While the first group consisted of individuals belonging to younger age groups (less than 40 years), the second group consisted of older individuals (more than 40 years). Similar signature patterns of the membership of the gut microbiomes were observed in individuals belonging to various age groups (G1: 0–10 years, G2: 10–30 years, G3: 30–40 years, G4: 40–50 years, G5: 50–60 years, G6: more than 60 years of age). These results suggest that the gut microbiomes of individuals have distinct age specific trends.

The diversity (in terms of the number of genera detected) of the gut microbes in older individuals was observed to be higher as compared to those in younger people. Certain groups of genera were also detected only in the older age groups (G4, G5 and G6) (Additional file 13). Furthermore, the increasing trend of relative homogeneity (i.e. lower intra-age group Jaccardian distances) with age indicates that, the gut microbial population becomes much more homogeneous across the individuals with age, irrespective of the geographies where they live (Additional file 1). Similarly, the Shannon diversity indices also showed noticeable increase with age and was found to stabilize after a certain age-group (Additional file 1). This indicates that with increasing age, the gut microbial population not only becomes consistent across individuals from various geographies, but its diversity increases till the age of 40 and stabilizes after that.

#### Overall properties of gut microbial interaction networks across different age-groups

Investigating the co-occurrence networks across individuals belonging to different age groups revealed a distinct change in the average degree of the nodes (genera) across age groups (Table 4). The average node degree of the lower age groups (G1, G2, G3) (7.1–9.0) were observed to be noticeably lower as compared to that of the older age groups (G4, G5, G6) (16.4–19.3). This indicates an increase in the functional inter-dependence among gut bacterial groups for individuals above the age of 40. Therefore, the gut microbial communities showed distinct age specific trends not only with respect to their composition (Fig. 7), but also with respect to certain properties of inter microbial association/exclusion networks.

**Table 4:** Overall properties of (A) co-occurrence network and (B) mutual exclusion network in gut microbiomes of individuals belonging to various age groups

Age groups	Number of vertices	Number of edges	Average Degree	Diameter	Avg shortest path length	Network density	Clustering coefficient	Network centralization
*A*								
G1 (0–10)	64	279	8.585	8	2.810	0.136	0.536	0.218
G2 (10–30)	57	206	7.103	7	2.419	0.127	0.440	0.329
G3 (30–40)	56	257	9.018	8	2.856	0.164	0.578	0.355
G4 (40–50)	72	704	19.288	8	2.260	0.272	0.666	0.325
G5 (50–60)	69	574	16.400	6	2.098	0.241	0.597	0.339
G6 (>60)	74	663	17.680	6	2.177	0.242	0.632	0.339
*B*								
G1 (0–10)	17	18	2.000	6	3.085	0.125	0	0.204
G2 (10–30)	26	26	1.926	6	3.487	0.077	0	0.217
G3 (30–40)	19	18	1.800	2	1.895	0.100	0	1.000
G4 (40–50)	52	55	2.075	6	2.302	0.041	0	0.875
G5 (50–60)	39	38	1.900	8	3.508	0.050	0	0.334
G6 (>60)	40	36	1.756	2	1.932	0.045	0	0.815

In addition to the increase in average node degree in the co-occurrence networks, an increase in the network centralization properties were observed in the gut microbial mutual exclusion networks for individuals in the age groups G3–G6. This indicates that in individuals above 30 years of age, there exist certain key microbial groups that strongly inhibit several other genera. Overall causes of these trends and their implications on physiology and gut health requires deeper investigation on the life-style habits, environmental exposure and dietary changes that normally happen with age. It also requires investigation of the compositional changes happening within the interaction networks at different age-groups.

#### Comparison of the microbial interaction networks across age groups

**Fig. 8:**
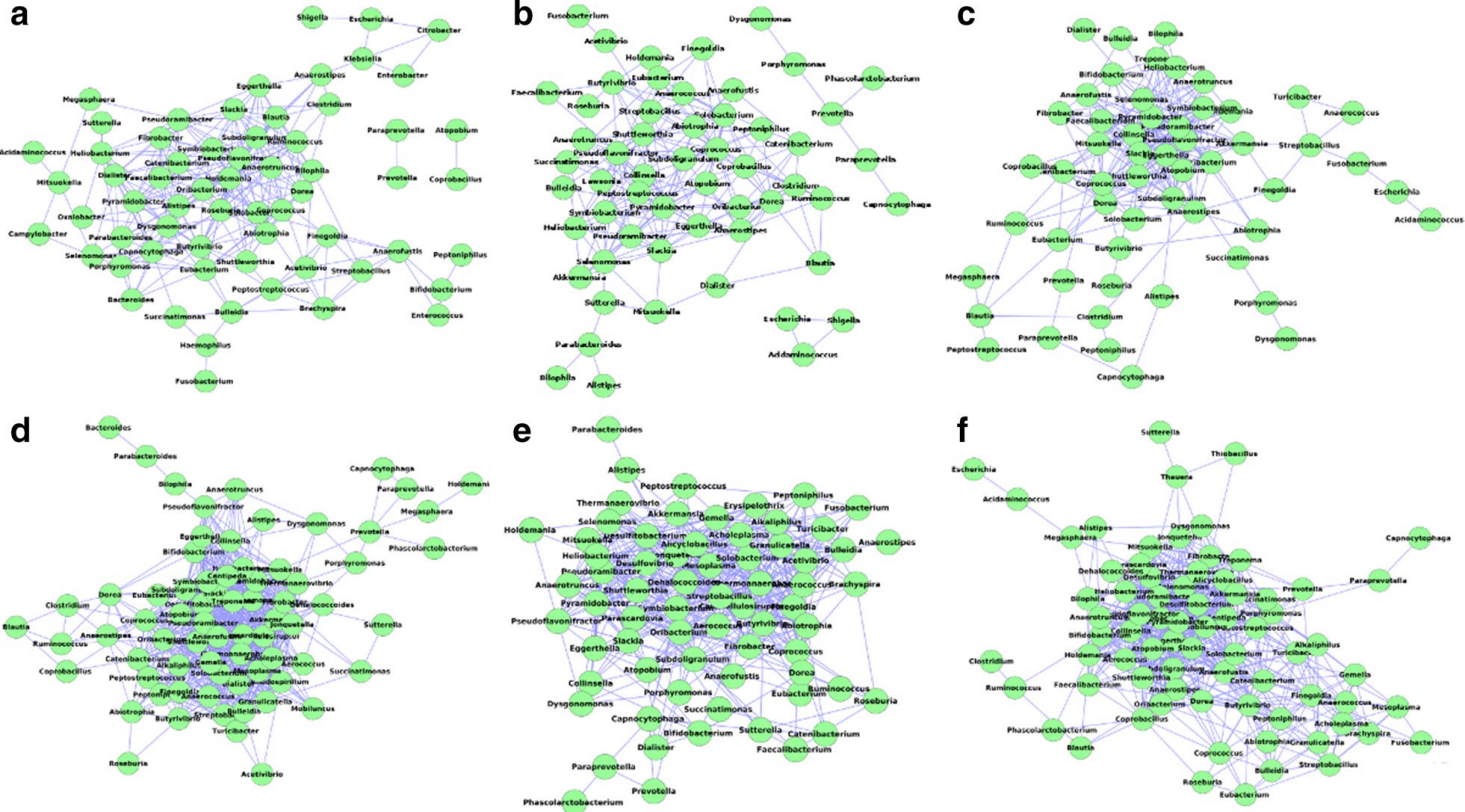
Gut microbial co-occurrence network in individuals belonging to the six age groups namely, **a** Group 1 (0–10 years) **b** Group 2 (10–30 years) **c** Group 3 (30–40 years) **d** Group 4 (40–50 years) **e** Group 5 (50–60 years) **f** Group 6 (above 60 years). The variations in the co-occurrence networks are depicted across the different age-groups

Visual inspection of the co-occurrence networks (Fig. 8) revealed similar characteristics across age groups. The networks were characterized by the presence of a central dense hub of co-occurring genera, connected with auxiliary smaller hubs. However, the density of the central hub was observed to increase with age of the individuals. Network density was observed to increase from 0.13 in G1 to 0.24 in G6. This observation, in line with the earlier observation of an increase of average degree of nodes, further indicates an increase in inter-dependence among the bacterial genera (especially those constituting the central hub) with age of the individuals.

**Fig. 9:**
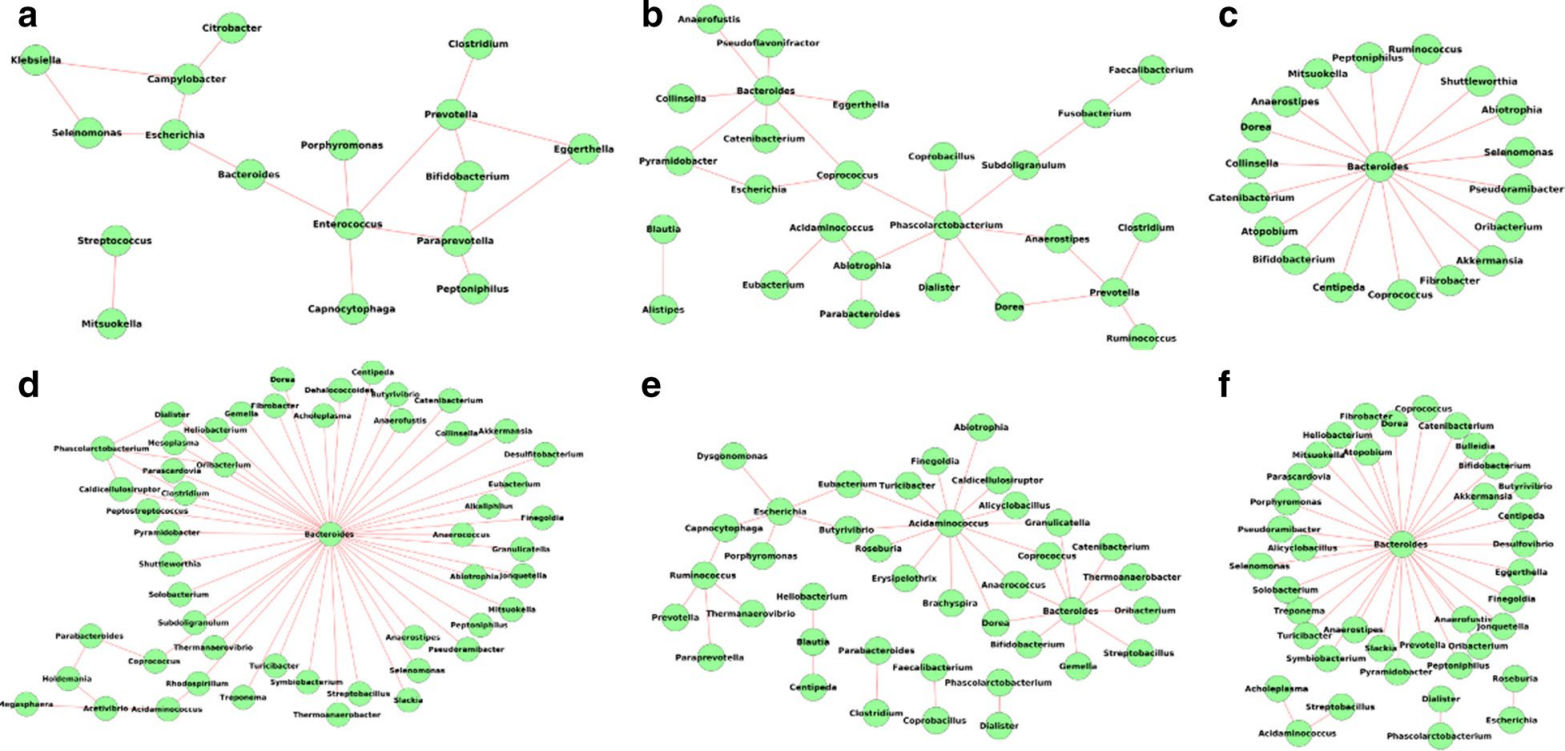
Gut microbial mutual exclusion network in individuals belonging to the six age groups namely, **a** Group 1 (0–10 years) **b** Group 2 (10–30 years) **c** Group 3 (30–40 years) **d** Group 4 (40–50 years) **e** Group 5 (50–60 years) **f** Group 6 (above 60 years). The variations in the mutual exclusion networks are depicted across the different age-groups

Analysis of the networks (Fig. 9) identified Bacteroides as a central genus having multiple mutual exclusion patterns with other genera in the gut microbiomes of individuals above 30 years. The number of exclusion relationships was found to be especially high in the 30–50 (G3, G4) and above 60 (G6) age groups. However, for the age group G5, although the number of mutual exclusion relationships involving Bacteroides was observed to be relatively less, additional central genera like Ruminococcus, Acidiminococcus and Escherichia were present in the network. In summary, the above observations indicate that the gut microbial community probably adapts with age a strong bi-partite ‘compartmentalized’ structure, wherein groups of genera show strong co-occurrence relationships with each other, with certain key genera strongly inhibiting the presence of the members of these groups.

#### Specific microbial interactions in the gut microbiome of middle aged and elderly individuals

Analyses of the microbiome networks indicated several genera that specifically existed in the interaction networks of individuals above the age of 40 (groups G4–G6) (Table 5). The functional characteristics of these genera were subsequently probed from literature. Genera like, Acholeplasma, Aerococcus, Treponema, Desulfovibrio and Brachyspira are found to contain species that are opportunistic pathogens (referred to as ‘Pathobionts’) [23–27]. Furthermore, two genera, namely Gemmella and Parascardovia, have been seen to be associated with opportunistic infections and dental caries, respectively [28–30]. It is known that there is a progressive decrease in the immunity of individuals with age. The decrease in immunity is likely to result in the entry of such opportunistic pathogens in the gut microbial community. Another interesting observation in the gut microbiome of elderly was the presence of thermophilic/alkaliphilic genera like Alkaliphilus, Caldicellulosiruptor, Thermoanaerobacter and Thiobacillus. Distinct physiological changes associated with age, disease and dietary habits may form the basis for the entry of such extremophiles. The functional implication of the presence of such genera in the guts of the elderly however needs further investigation.

**Table 5:** Bacterial species found exclusively in older age groups (G4–G6)

Genus	Order	G1	G2	G3	G4	G5	G6
Acholeplasma	Acholeplasmatales	0	0	0	1	1	1
Gemella	Bacillales	0	0	0	1	1	1
Parascardovia	Bifidobacteriales	0	0	0	1	1	1
Alkaliphilus	Clostridiales	0	0	0	1	1	1
Desulfitobacterium	Clostridiales	0	0	0	1	1	1
Dehalococcoides	Dehalococcoidales	0	0	0	1	1	1
Mesoplasma	Entomoplasmatales	0	0	0	1	1	1
Aerococcus	Lactobacillales	0	0	0	1	1	1
Jonquetella	Synergistales	0	0	0	1	1	1
Thermanaerovibrio	Synergistales	0	0	0	1	1	1
Granulicatella	Lactobacillales	0	0	0	1	1	1
Mobiluncus	Actinomycetaceae	0	0	0	1	0	1
Alicyclobacillus	Bacillales	0	0	0	0	1	1
Desulfovibrio	Desulfovibrionales	0	0	0	0	1	1
Brachyspira	Spirochaetales	0	0	0	0	1	1
Caldicellulosiruptor	Thermoanaerobacterales	0	0	0	1	1	0
Thermoanaerobacter	Thermoanaerobacterales	0	0	0	1	1	0
Rhodospirillum	Rhodospirillales	0	0	0	1	0	0
Erysipelothrix	Erysipelotrichales	0	0	0	0	1	0
Thiobacillus	Hydrogenophilales	0	0	0	0	0	1
Thauera	Rhodocyclales	0	0	0	0	0	1

### Influence of diet on the composition and network properties of the gut microbiomes of the various nationalities

The probable relationships between the population level dietary statistics for different nationalities (obtained from http://faostat3.fao.org/download/FB/FBS/E) and the gut microbial composition profile of the corresponding microbiomes were investigated using partial least square (PLS) regression analysis. The analysis indicated varied degree of correlations between various genera and per-capita dietary intakes of the different nationalities. For all the genera, the maximum correlation was observed for the first component of the partial least square regression (PC1). Further, the strength of the correlations (of the genera abundances with the component) ranged from as high as 0.91 (observed for Bulleidia) to 0.46 (observed for Peptostreptococcus) (Additional file 14). This suggests probable influence of diet on the abundances of various genera present in the gut.

Further, a core set of 28 genera, identified to be present in four (out of the eight) nationalities, was observed to have regression (R^2^) values greater than 0.64 (R > 0.8) with the PC1. Subsequently, specific relationships between the per-capita intakes of the different dietary components with the abundances of these 28 gut microbial genera were investigated (Additional file 15). Based on the distinct patterns of association with the different dietary factors, the present analysis identified three distinct groups of genera. The first group consisted of the genera Prevotella, Paraprevotella, Succinatimonas, Porphyromonas and Mitsuokella. This group was observed to have a strong association with the dietary pulse content, followed by aquatic products, starchy roots, sugar crops and vegetables (specifically Prevotella and Paraprevotella). On the other hand, a negative association was observed for this group with food categories consisting of meat, animal fats and vegetable oils. Prima facie, this group of genera seems to have an association with vegetarian diet. Interestingly, earlier studies have indicated presence of such genera in the gut of individuals having higher intake of vegetables and dietary fibre and lower intake of fats [18, 31]. The second group consisted of genera like, Roseburia, Butyrivibrio, Allistipes, Abiotropha, Bulleidia and Finegoldia, which were observed to have a positive association with a variety of food sources, with the exception of pulses, tree nuts and fruits. The third group consisted of commensal genera, including Bacteroides, Faecalibacterium, Clostridium, Ruminococcus, Blautia and  Phascoloractobacterium. Most of the genera belonging to this group had a high positive association with Berry index (indicative of diversity of  a diet) as well as with healthy food diversity index [32]. These results are in line with earlier studies which have indicated the positive association of these genera (especially Faecalibacterium and Ruminococcus) with the health of the human host [22, 33]. However, in terms of the association with the different food categories, two sub-groups were observed among the members of this group. While the members of the first sub-group (Bacteroides, Clostridium, Phascoloractobacterium, Blautia and Peptoniphilus) were observed to have a positive association with consumption of fish, aquatic products and eggs, those belonging to the second sub-group (Faecalibacterium, Pyramidobacter, Pseudoflavonifractor, Slackia and Eggerthela) were observed to have a noticeably higher association with tree nuts and fruits. Given that this is an indirect association study, the basis of such associations for many of these genera needs to be validated experimentally. It is interesting to note that two of  the identified genera (Bacteroides and Faecalibacterium) are in line with previous findings highlighting the impact of dietary patterns on their abundances [21, 34, 35]. It has been observed previously that Bacteroides is associated with high protein-based animal-content rich diet, whereas dietary-fibre rich content (e.g. vegetables, fruits) causes an increase in the abundance of Faecalibacterium species [35].

Subsequently, a similar analysis was performed to evaluate whether the level of gut microbial co-occurrence/mutual-exclusion networks is dependent on the country-specific dietary habits (Additional file 16). Correlation between the various microbial network properties and Berry as well as HFD indices, measures for the number of food components consumed and health values of the food components, respectively, indicated interesting findings (Additional file 16). The observed decrease in the density of the co-occurrence network with increase in Berry index suggests that with increase in the variety (or diversity) of food consumption, there is a reduced functional interdependency amongst the microbial community. On the other hand, with the increase of HFD index, the key microbial players (hubs), represented as central nodes in the co-occurrence networks were found to have increased connections (i.e. higher network centralization). This suggests that with increase in health value of the diet, the regulatory effect of the key microbial players on other microbes increases.

A probable reason for this could be that the increase in diet diversity could support varied groups of bacteria with diverse nutritional requirements. The positive correlation between HFD index (as well as vegetable, meat, egg, and aquatic product consumption) with various network properties of mutual exclusion networks indicates that the exclusion patterns among microbial genera increases with healthy food diet. On the other hand, the negative interactions amongst the microbes were found to decrease with increase in number of food items consumed (as indicated by Berry index).The observed negative correlation between the number of food items consumed and the network properties in both co-occurrence and co-inhibitory networks suggest that with increase in the variability of the nutritional sources (from the diet), the interdependencies amongst the microbial community not only decreases, but also abets competition (thereby supporting a diverse eco-system). The different food components were also observed to have distinct influences on the properties of the co-occurrence and mutual exclusion networks.

## Discussion

The current computational analysis was performed with the objective of profiling not only the microbial composition landscape of gut microbiomes of individuals belonging to different geographies and age-groups, but also their microbial interaction patterns. To the best of our knowledge, this is the first study of its kind performed on a large dataset (399 individuals from eight nationalities). The study indicated distinct geography as well as age-group specific trends, with respect to the composition, diversity and intra-group heterogeneity of gut microbial communities of individuals. However, unlike the signature trends in the community composition and diversity of gut microbes across geographies, the trends pertaining to gut microbial association/mutual-exclusion networks were found to be relatively cross-geographic, with distinct geography specific trends in the overall network properties of gut microbiomes. These specific patterns could be due to the inherent differences in the diet of individuals belonging to different nationalities. In order to evaluate whether the observed geography specific gut microbial networks are in accordance with the dietary habits of different nationalities, we attempted to investigate the relationships between the composition and network properties of the gut microbiome for different nationalities and the corresponding population level dietary statistics. Since the dietary intake patterns of the subjects whose gut microbiomes were analyzed in the current analysis were not profiled in the original studies, we considered nation-specific diet intake patterns from the Food and Agricultural Organization of the United Nations information repository (http://faostat3.fao.org/download/FB/FBS/E). The analysis showed a correspondence between the per-capita intakes of the various food components across different nationalities (Additional file 17) and the respective gut microbiome structures.

Interestingly, many patterns observed in the diet analysis does corroborate with results from previous studies. For example, some genera belonging to Clostridia class, like Roseburia, Butyrivibrio, Eubacterium and Clostridium, were observed to be positively associated with the intake of meat (including fish), animal fat, milk, eggs and oils. This is in line  with previous studies [18, 36] which had shown that shifting to a high fat diet causes a shift in gut microbial composition having increased abundances of these specific genera. Faecalibacterium was observed to have a noticeably higher association with intake of vegetables and fruits as compared to other Firmicutes genera like Roseburia and Eubacterium (Additional file 15), which is in line with the observation reported in a previous study [33]. The highest number of positive associations with different food items observed for Bacteroides could be a reflection of the high substrate versatility of species belonging to this genus. Notably, genome of *Bacteroides thetaiotamicroton* has been shown to encode more than 200 families of carbohydrate active enzymes, indicating a higher variation in its substrate preferences [37, 38]. In this regard, the most interesting observation made in the present study pertains to the differential association of dietary components with the driver genera for the two well known enterotypes [7], namely Bacteroides and Prevotella. In contrast to the positive associations of Bacteroides with the wide range of dietary components (especially those rich in protein and animal diet), Prevotella was observed to be specifically associated with vegetarian contents like pulses, starchy roots, sugar crops and vegetables. This observation is in line with that obtained in a previous study which investigated the linkages between changes of gut microbial composition in individuals with long-term dietary patterns [34].

In addition, association between the intakes of various food categories with the overall properties of the co-occurrence networks was more evident from the present study. It has been reported earlier that, based on the resources available in an environment, the functional interdependence or the interactions between co-occurring genera may be driven by metabolite exchanges between them [2]. In the present study, the density of the co-occurrence networks, indicative of the functional interdependencies, were observed to decrease with consumption of food products like vegetables, eggs, tree nuts, fruits, milk, aquatic and sea food. On the other hand, the interdependencies amongst resident microbes in the gut were found  to increase with the consumption of meat, animal fats, cereals, pulses and sugars/sweeteners. This is in accordance with the reported hypothesis of dysbiosis in the gut microbiome with consumption a high-fat-high-protein diet [18, 33, 36]. Interestingly, increased functional interdependencies with higher nutritional status have also been reported earlier [22]. Furthermore, the decrease in the functional interdependency amongst microbial community (network density) with food diversity could explain the apparent differences observed in the gut microbial co-occurrence networks for the Danish and the Spanish populations. Although belonging to the same continent, the Danish individuals were observed to have a much higher degree of functional interdependence (in terms of the average degree of nodes) in their gut co-occurrence networks as compared to the Spanish individuals. This trend is explained by the differences in the dietary patterns between the Spanish and Danish populations. The Spanish population have an evenly distributed diet compared to Danish population, indicated by a higher Berry Index as well as a higher HFD (Additional file 18). Previous studies have indicated that the microbes having similar nutrient preferences tend to co-occur together [39]. Consequently, the gut of individuals having a homogenous diet (that is dominated by specific constituents) is likely to favour the growth of inter-dependent species having strong co-occurrence relationships among each other. On the other hand, gut of individuals having a highly variable or diverse diets (in terms of the different constituents) are likely to result in the growth of diverse bacterial groups with different nutrient preferences having lesser functional interdependence/competition among each other. The higher diversity in diet could therefore be a reason for the lesser interdependence among microbial genera observed for the Spanish individuals.

The most interesting observation of the current study is that the similarities in the genera level composition of interaction networks were not observed to be dictated by ethnicities or geographical proximity. A key example is the similarity between the microbial interaction networks of the Chinese and American populations (as compared to Japanese/Indian and European populations). The cross ethnicity similarities in the interaction patterns of gut microbiota is an interesting observation that requires further validation and profiling of the environment, hygiene as well as life-style habits (including dietary intake) of individuals belonging to different nationalities.

Distinct similarities in the gut microbial interaction networks, based on the age of the individuals were observed from the present study. Networks obtained for individuals below 30 years of age (G1–G3) were observed to be similar and distinct from those obtained for the age groups above 40 years. A key distinguishing factor was the increased functional interdependence between bacterial groups in the networks in the higher age groups.

In spite being the first of its kind study, the current study has distinct limitations primarily pertaining to the composition of cohorts constituting the age-groups and geographies. First, since some of the cohorts, especially those corresponding to French and Italian populations, have lower sample size, the reliability of the results obtained are specifically lower for these nationalities. Further, there is an inherent bias in the composition of individuals constituting the age-wise cohorts below 10 years of age, which primarily belonged to Indian nationality. Furthermore, sub-population specific biases could also occur in the diet-microbiota association analysis performed in the current study. This is because, while the dietary intake patterns used in the current study are population-level statistics obtained for entire nationalities, the gut microbiomes are only obtained from specific individuals in distinct neighbourhoods of a given country. Given the global nature of this study, as well as a more or less even representation of individuals in a majority of cohorts, a concordance was observed between the results obtained in the current study with previous reports, potential biases mentioned above. These inferences from the current study are likely to form the basis for future metagenomic investigations across much larger cohorts of individuals from specific regions.

## Conclusions

The present study reports a comprehensive analysis of gut microbial composition of individuals belonging to different geographies and age-groups, as well as the microbial interaction patterns in these microbiomes. Although a conserved group of genera was found to inhabit most of the datasets analyzed, a clear geography-specific trend of microbial composition was also noted. Inter-individual heterogeneity in the composition of gut microbial communities also exhibited geography specific variations. Analysis of microbial interaction networks pertaining to the analyzed gut microbiomes from different geographies revealed that despite the presence of equivalent number and types of genera in these networks, the connectivities (i.e. the set of interactions) can drastically vary across geographies. A meta-analysis incorporating population level dietary information, indicated the probable role of dietary habits in shaping the gut microbiome composition as well as the inter-microbial interactions. The findings from the present study also indicate that the gut microbiota becomes more diverse with age, wherein several genera with similar functional profiles such as alkaliphiles, opportunistic pathogens, and sulfate reducers tend to co-occur specifically in the gut microbiomes of the middle-aged and elderly. The inferences drawn from this study are expected to form the basis for future metagenomic investigations involving much larger cohorts from various regions/age-groups/health-status, and can potentially lead to translational outcomes such as dietary/therapeutic recommendations.

## Methods

### Datasets used

Publicly available 399 gut metagenomes were downloaded from the following sources. Assembled contigs corresponding to 90 American gut metagenomes were downloaded from the HMP DACC website (http://www.hmpdacc.org/HMASM/) (Table 1 in Additional file 19). These metagenomes were sequenced as part of the human microbiome project (HMP) and previously analyzed by Ghosh et al. [6]. Contigs corresponding to gut metagenomes belonging to the French, Italian and Japanese individuals, previously analyzed by Arumugam et al. [7], were downloaded from http://www.bork.embl.de/Docu/Arumugam_et_al_2011/downloads.html. Gut metagenomic contigs corresponding to 81 Danish and 35 Spanish individuals, previously analyzed by Qin et al. [4], were obtained from http://gutmeta.genomics.org.cn/. Assembled contigs from 144 Chinese gut metagenomes, previously studied by Li et al. [8], were downloaded from http://gigadb.org/dataset/100036. In addition, gut metagenomes from 22 Indian children, previously analyzed by Gupta et al. [6] and Ghosh et al. [22], were downloaded from http://www.ncbi.nlm.nih.gov/Traces/sra. Since the lengths of the sequences constituting these metagenomes were comparatively smaller (about 400 base pairs), an additional step of sequence assembly was performed on each of these datasets for obtaining longer contigs. The nation-specific dietary pattern data was obtained from the website of the Food and Agricultural Organization of the United Nations (http://faostat3.fao.org/download/FB/FBS/E).

Previous studies have noted that a major concern with using samples from multiple studies is the presence of study-specific biases [40]. To address this issue, we have performed several analysis (Additional file 19) in order to be sure that such biases in the datasets used (wherever present) do not significantly effect the results of this study.

### Relative homogeneity of gut microbiomes across individuals from various nationalities/age-group

The homogeneity of gut microbial community across individuals from a specific geography/age-group was obtained using Jaccardian indices. For a given geography/age-group, pair wise Jaccardian indices were obtained from the corresponding gut genera profiles using the following formula:

$$Jaccard Index = \frac{{Genus_{11} }}{{Genus_{11} + Genus_{01} + Genus_{10} }}$$

where, Genus_11_ are number of pairs of datasets in which the genus is present in both.

Genus_10_ and Genus_01_ are number of pairs of datasets where genus is present in one member of the pair and absent in another one.

Jaccard distance was then calculated using the formula,

$$Jaccard distance = \text{1}-JaccardIndex$$

Thus, Jaccardian indices would be higher in individuals (belonging to a specific nationality/age-group) having relatively higher homogeneity (that is relatively lesser variation) across their gut microbial community structure.

### Profiling complexity of gut microbiomes belonging to different groups of individuals using Shannon diversity indices

While relative homogeneity indicates intra-sample variability across gut microbiomes of a given group, Shannon diversity indices provide measures of how complex the microbiomes are in terms of the abundances of various taxonomic groups. For each gut microbiome, the Shannon diversity indices were computed as:

$${H^{'} = - \sum\limits_{i = 1}^{N} {\mu_{i} { \ln }\mu_{i} } }$$

where, N is total number of genera and μ_i_ is the proportion of microbiomes belonging to the ith genera.

### Identifying group specific signatures of gut microbial composition

For identifying groups of genera that are significantly over or under represented in different groups, the abundance values of various genera in the gut microbiomes of each group were compared with those belonging to all other groups. For this purpose, the STAMP analysis pipeline [41] was used. The comparisons were performed using Welch’s t-test (with multiple test corrections and false detection rate obtained using Benjamini Hochberg tests). Differentially abundant genera with corrected *p* value less than 0.001 were identified to be significantly over- or under-represented in the given group.

### Network inference methodology

#### Obtaining genera abundance matrices corresponding to gut microbiomes of individuals belonging to a nationality

Metagenomic contigs corresponding to each gut microbiome were taxonomically classified using the approach adopted by Ghosh et al. [22]. In this approach, a similarity search of the metagenomic contigs was first performed against a reference database of 2352 bacterial/archaeal genomes [22]. BLASTn output thus obtained was filtered (hits with percentage identity >65 and e value <10e−10 were retained). Filtered results were provided as input to the DiScRIBinATE method [42] for obtaining the final taxonomic assignment of the metagenomic contigs (constituting each dataset).

The abundance profile of various bacterial groups (at genera level) for each gut microbiome was obtained as described in Additional file 20. Genera that were identified in at least 30 % of the metagenomes were only considered for subsequent analysis (genera absent in 70 % of datasets were filtered out). This was done to limit the number of ‘zero abundance values’, which may otherwise lead to false/biased correlation results. The abundance profiles of bacterial genera in the gut microbiomes belonging to the different nationalities were then grouped separately and represented as separate abundance matrices (for each nationality).

#### Obtaining gut microbial interaction networks for different nationalities

For individuals belonging to each nationality, corresponding abundance matrix was provided as input to the network analysis tool (NAMAP) developed as part of this study (described in Additional file 20). The centrality measures of the microbial interaction networks were calculated using the i-graph module (C language) [43], whereas network visualization was performed using cytoscape and community-analyzer [44, 45]. The gut microbial networks for the corresponding nationality were then inferred using the following strategy. Correlations between each pair of genera were calculated using the Spearman’s ranked correlation coefficients. The statistical significance of each of these correlations was then obtained using the ReBoot approach implemented in the CoNet method [1, 22].

This approach finds the statistical significance of a given correlation (between a given pair of genera) by comparing the *ReSampling and null* distributions of correlation values (obtained from the given abundance values). These distributions were obtained by performing 100,000 iterations.

The correlation values obtained for both these distributions were then compared using the z-score (described in Additional file 20). Co-occurring and mutually exclusive pairs of genera were identified as those for which obtained z-score was greater than 1.96 and less than −1.96, respectively (i.e. with P < 0.01). The co-occurrence and mutual exclusion interaction networks were then constructed with genera as nodes and edges between the co-occurring and mutually exclusive pairs of genera, respectively.

Eight gut inter-microbial interaction networks were thus obtained. For each network, various network properties were computed and compared (Additional file 4).

#### Analysis based on age groups

Except for American samples (for which age information was unavailable), the samples were grouped based on the age of individuals from various nationalities. Six groups were formed (Table 1). The method described in the above section was followed to further analyze the groups.

### Associating the dietary patterns corresponding to the different nationalities with the structure and network properties of the gut microbiomes

In order to investigate whether (and if so, how much) the nation-specific dietary patterns influenced the structure and the network properties of gut microbiomes (across various nationalities), a PLS regression based analysis was performed. Since the diet data for most of the microbiomes used in the current study were unavailable (that is, not recorded in the original studies on these microbiomes), the nation-specific dietary pattern data was obtained from the website of the Food and Agricultural Organization of the United Nations (http://faostat3.fao.org/download/FB/FBS/E). This information repository contains the production, consumption, export as well as the consumption of the various food items for around 113 countries. From this repository, nation specific intake of the dietary consumption patterns of the various food items corresponding to the 8 different nationalities was filtered out (Additional file 18). Further, from these dietary patterns, the overall variability in the diets of the various nationalities was then quantified using two different food diversity indices namely, Berry index and healthy food diversity (HFD) index. While Berry index just considers the increase in number of food components, HFD also considers their value [32]. Subsequently, a PLS regression was performed with the overall dietary diversity as well as the consumption patterns of the various dietary components as predictor variables and the median genera abundances and the network properties of the gut co-occurrence and mutual exclusion networks (Table 1) as the response variables for the various nationalities.

## Additional files

10.1186/s13099-016-0099-z A) Jaccardian distances between the microbial genera detection profiles within the gut microbiomes of individuals belonging to different nationalities. B) Shannon diversity indices of the gut microbial communities of individuals from three different groups of nationalities. C) Jaccardian distances between the microbial genera detection profiles within the gut microbiomes of individuals belonging to different age-groups. D) Shannon Diversity of the gut microbiomes of individuals from various age-groups.
10.1186/s13099-016-0099-z Significantly over/under-represented genera in the gut microbiomes of the American Individuals as compared to those of the others. Significantly different genera were identified using Welch’s t-test with P-value < 0.05, corrected using Benjamini-Hochberg FDR method for multiple test corrections. Further stringency was established using mean ratio of mean proportions to be 1.5. All tests were performed using the STAMP analysis package.
10.1186/s13099-016-0099-z Heatmap showing the normalized abundances of major genera in the gut microbiomes of American individuals. Only those genera, present in at least 40 % of the individuals with a minimum abundance of 0.05, have been shown in the heatmap. Red color signifies that the genus is either absent or present in low abundance, whereas the green color signifies that it is highly abundant.
10.1186/s13099-016-0099-z Centrality measures of different genera in the gut microbiomes of American, China, Denmark, France, India, Italy, Japan and Spain for co-occurrence and mutual exclusion networks.
10.1186/s13099-016-0099-z Heatmap showing the normalized abundances of major genera in the gut microbiomes of Danish individuals. Only those genera, present in at least 40 % of the individuals with a minimum abundance of 0.05, have been shown in the heatmap. Red color signifies that the genus is either absent or present in low abundance, whereas the green color signifies that it is highly abundant.
10.1186/s13099-016-0099-z Heatmap showing the normalized abundances of major genera in the gut microbiomes of Spanish individuals. Only those genera, present in at least 40 % of the individuals with a minimum abundance of 0.05, have been shown in the heatmap. Red color signifies that the genus is either absent or present in low abundance, whereas the green color signifies that it is highly abundant.
10.1186/s13099-016-0099-z Significantly over/under-represented genera in the gut microbiomes of the Chinese Individuals as compared to those of the others. Significantly different genera were identified using Welch’s t-test with P-value < 0.05, corrected using Benjamini-Hochberg FDR method for multiple test corrections. Further stringency was established using mean ratio of mean proportions to be 1.5. All tests were performed using the STAMP analysis package.
10.1186/s13099-016-0099-z Heatmap showing the normalized abundances of major genera in the gut microbiomes of Chinese individuals. Only those genera, present in at least 40 % of the individuals with a minimum abundance of 0.05, have been shown in the heatmap. Red color signifies that the genus is either absent or present in low abundance, whereas the green color signifies that it is highly abundant.
10.1186/s13099-016-0099-z Significantly over/under-represented genera in the gut microbiomes of the Japanese Individuals as compared to those of the others. Significantly different genera were identified using Welch’s t-test with P-value < 0.05, corrected using Benjamini-Hochberg FDR method for multiple test corrections. Further stringency was established using mean ratio of mean proportions to be 1.5. All tests were performed using the STAMP analysis package.
10.1186/s13099-016-0099-z Heatmap showing the normalized abundances of major genera in the gut microbiomes of Japanese individuals. Only those genera, present in at least 40 % of the individuals with a minimum abundance of 0.05, have been shown in the heatmap. Red color signifies that the genus is either absent or present in low abundance, whereas the green color signifies that it is highly abundant.
10.1186/s13099-016-0099-z Genera occurrences in the common networks between specific pairs of nationalities.
10.1186/s13099-016-0099-z Order-level affiliations of the various genera constituting the gut microbial networks of different geographies, along with the number of co-occurrence and mutual exclusion interactions between them.
10.1186/s13099-016-0099-z List of genus occurring only in older age groups in gut microbial compositions.
10.1186/s13099-016-0099-z Regression coefficients of the abundance patterns of various genera with first component (PC1) of the PLS regression analysis.
10.1186/s13099-016-0099-z Heatmap showing the rank-normalized PLS coefficients of association of the nation specific per-capita intakes of the various food components and the food diversity indices (Berry Index and HFD) with the median abundances of the various genera in the corresponding gut microbiomes. Red color signifies low coefficient of association between the per-capita intakes of food components and the genera whereas the green color signifies a high coefficient.
10.1186/s13099-016-0099-z Heatmap showing the rank-normalized PLS coefficients of association of the nation specific per-capita intakes of the various food components and the food diversity indices (Berry Index and HFD) with the network properties of the (a) co-occurrence and (b) mutual exclusion networks observed for the gut microbiomes of the corresponding nationalities. Red color signifies low coefficient of association between the per-capita intakes of food components and the network properties whereas the green color signifies a high coefficient.
10.1186/s13099-016-0099-z Heatmap showing the rank normalized median of per-capita intakes of the various food components across the different nationalities. Red color signifies low per-capita intakes of the food component whereas the green color signifies a high per-capita intakes.
10.1186/s13099-016-0099-z Nation specific intake of the various food components (computed for 1000 individuals) obtained from the website of the Food and Agricultural Organization of the United Nations and the food diversity indices (Berry Index and Healthy Food Diversity (HFD) index computed for each geographic region.
10.1186/s13099-016-0099-z Evaluation of possible biases arising due to differences in datasets used in the present study and effects of such biases on the results.
10.1186/s13099-016-0099-z Short description of the network analysis tool, NAMAP, developed for this study.
