# Supplementary figures and images for: Global investigation of composition and interaction networks in gut microbiomes of individuals belonging to diverse geographies and age-groups
Source: Gut Pathog. 2016 May 6;8:17. doi: 10.1186/s13099-016-0099-z (PMC4858888; doi:10.1186/s13099-016-0099-z)

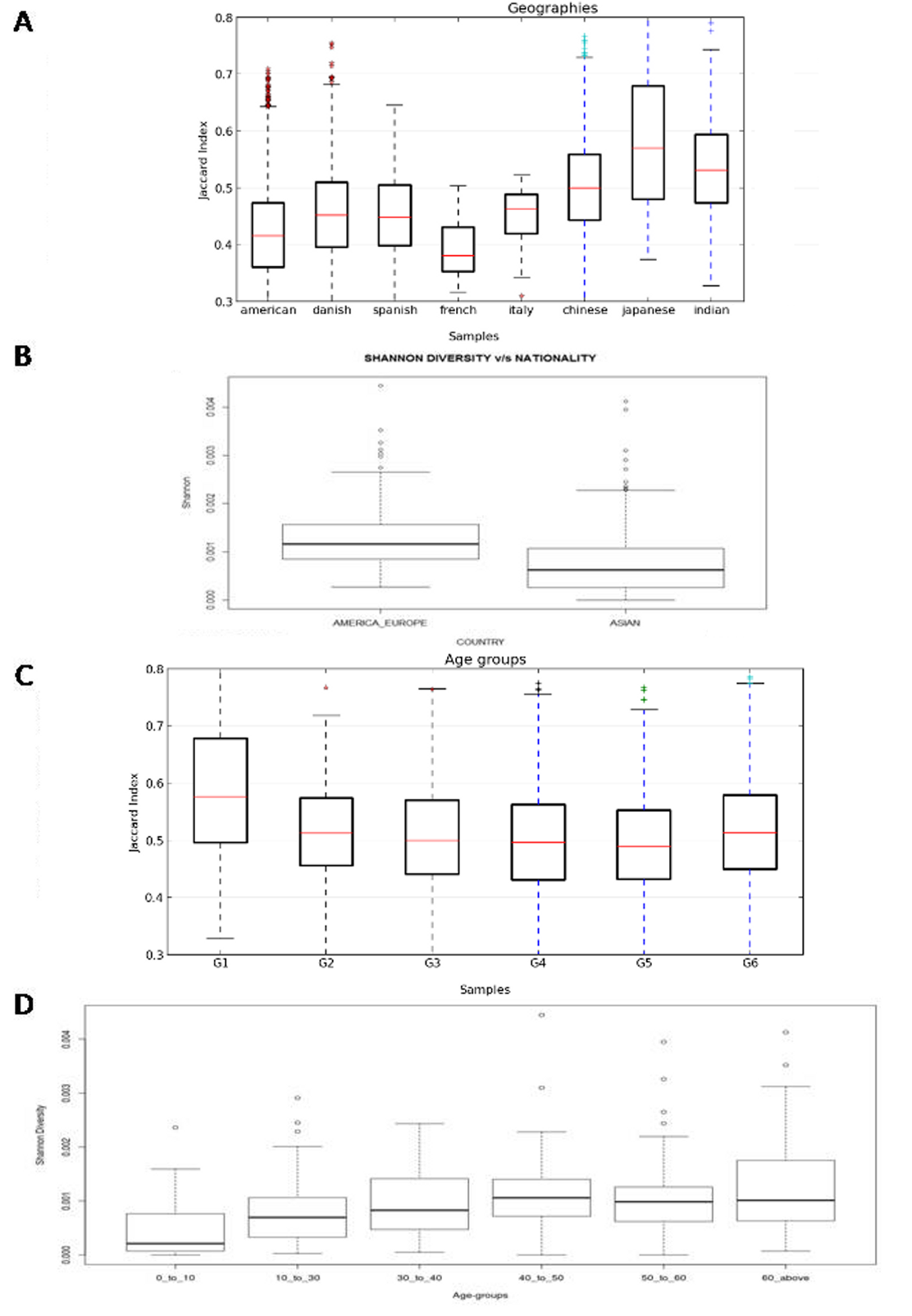

Supplement: Supplementary file 1 — 10.1186/s13099-016-0099-z A) Jaccardian distances between the microbial genera detection profiles within the gut microbiomes of individuals belonging to different nationalities. B) Shannon diversity indices of the gut microbial communities of individuals from three different groups of nationalities. C) Jaccardian distances between the microbial genera detection profiles within the gut microbiomes of individuals belonging to different age-groups. D) Shannon Diversity of the gut microbiomes of individuals from various age-groups. [file 13099_2016_99_MOESM1_ESM.tif]

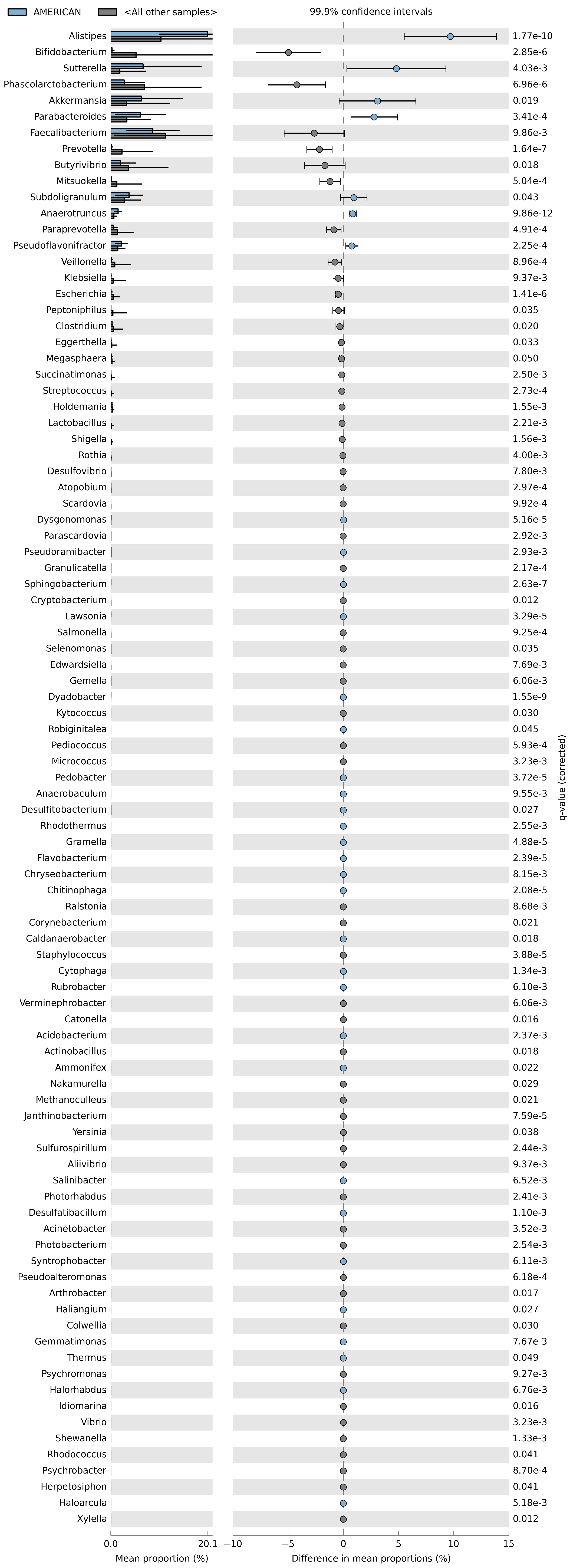

Supplement: Supplementary file 2 — 10.1186/s13099-016-0099-z Significantly over/under-represented genera in the gut microbiomes of the American Individuals as compared to those of the others. Significantly different genera were identified using Welch’s t-test with P-value < 0.05, corrected using Benjamini-Hochberg FDR method for multiple test corrections. Further stringency was established using mean ratio of mean proportions to be 1.5. All tests were performed using the STAMP analysis package. [file 13099_2016_99_MOESM2_ESM.tif]

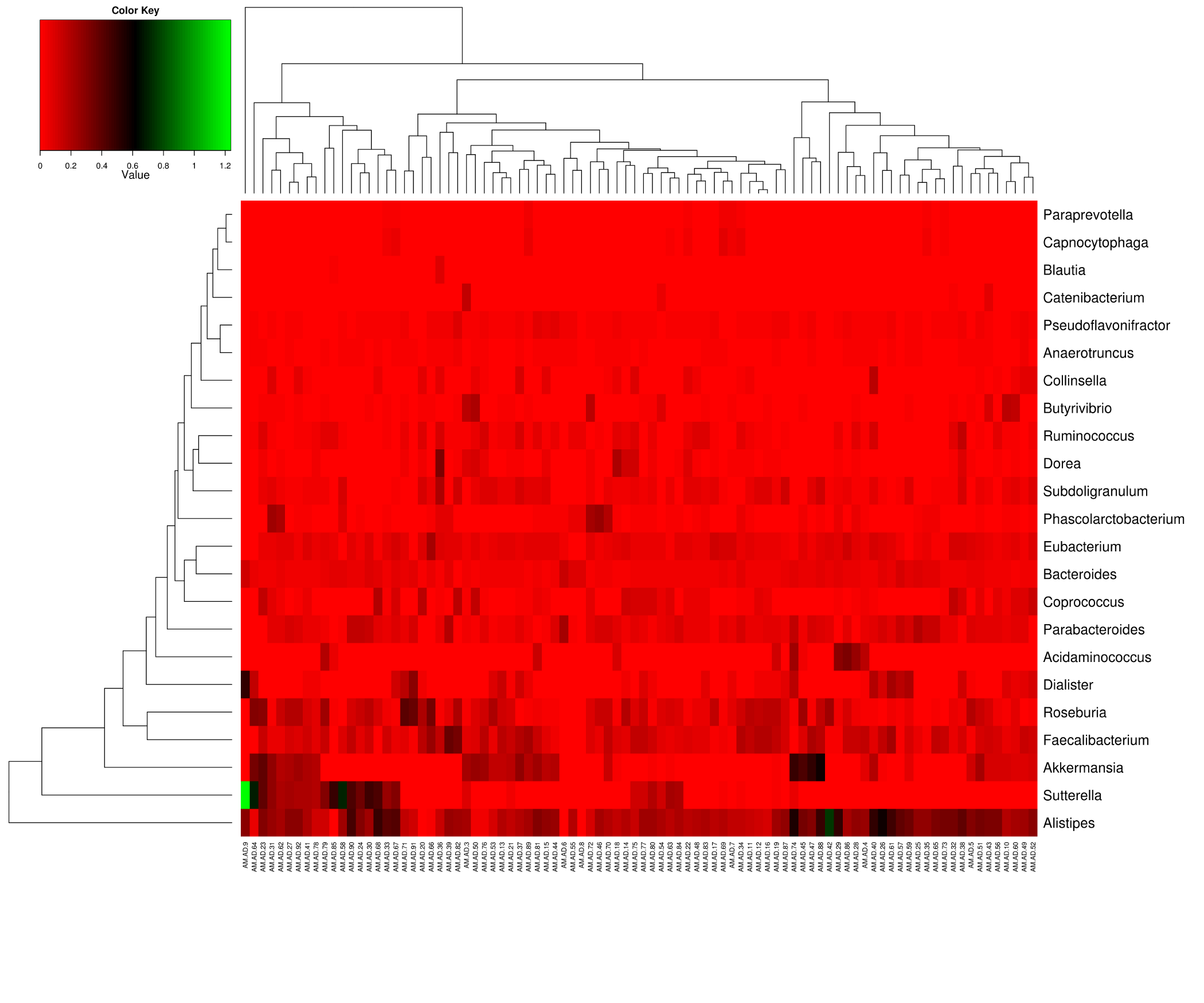

Supplement: Supplementary file 3 — 10.1186/s13099-016-0099-z Heatmap showing the normalized abundances of major genera in the gut microbiomes of American individuals. Only those genera, present in at least 40 % of the individuals with a minimum abundance of 0.05, have been shown in the heatmap. Red color signifies that the genus is either absent or present in low abundance, whereas the green color signifies that it is highly abundant. [file 13099_2016_99_MOESM3_ESM.tif]

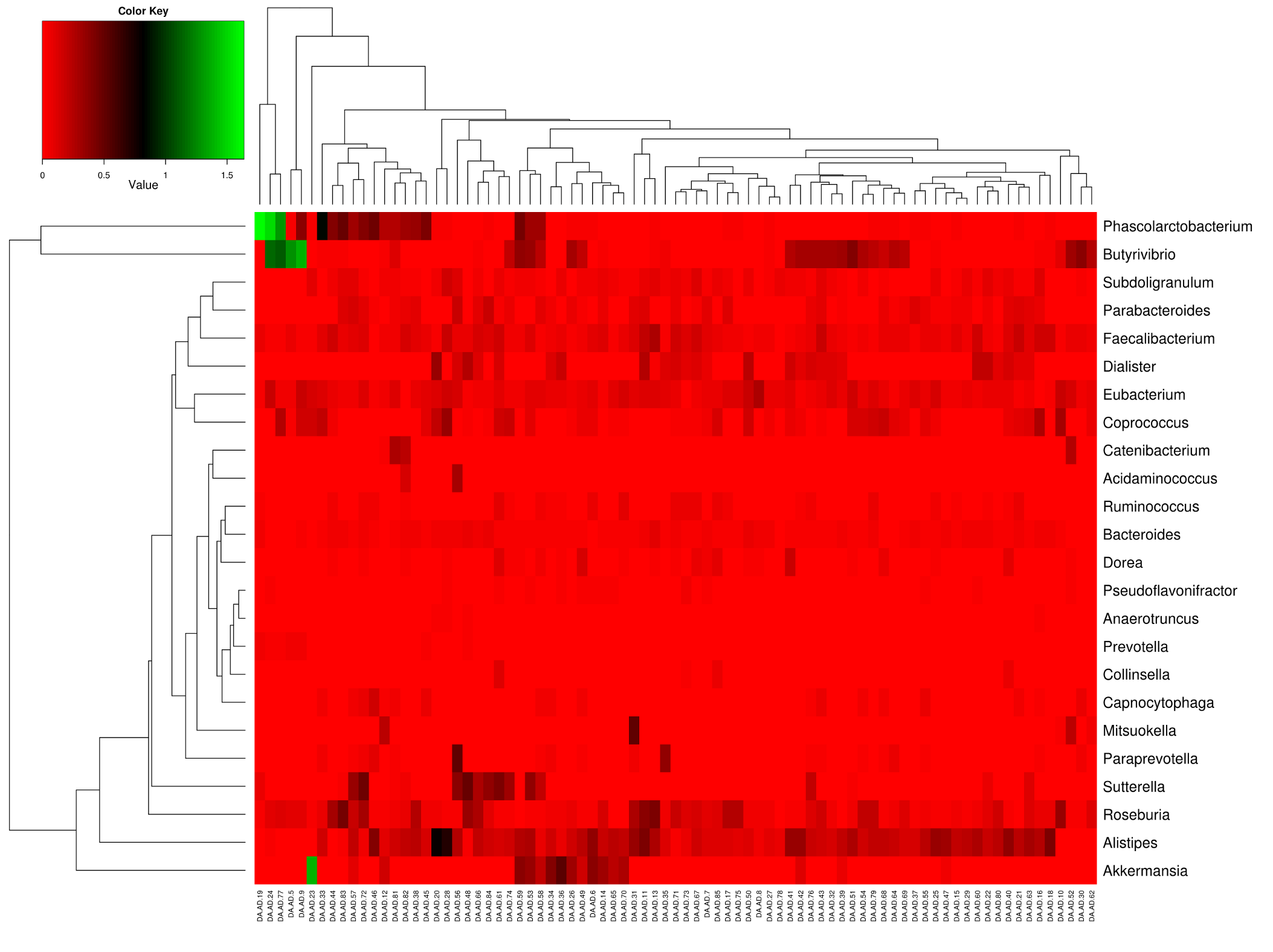

Supplement: Supplementary file 5 — 10.1186/s13099-016-0099-z Heatmap showing the normalized abundances of major genera in the gut microbiomes of Danish individuals. Only those genera, present in at least 40 % of the individuals with a minimum abundance of 0.05, have been shown in the heatmap. Red color signifies that the genus is either absent or present in low abundance, whereas the green color signifies that it is highly abundant. [file 13099_2016_99_MOESM5_ESM.tif]

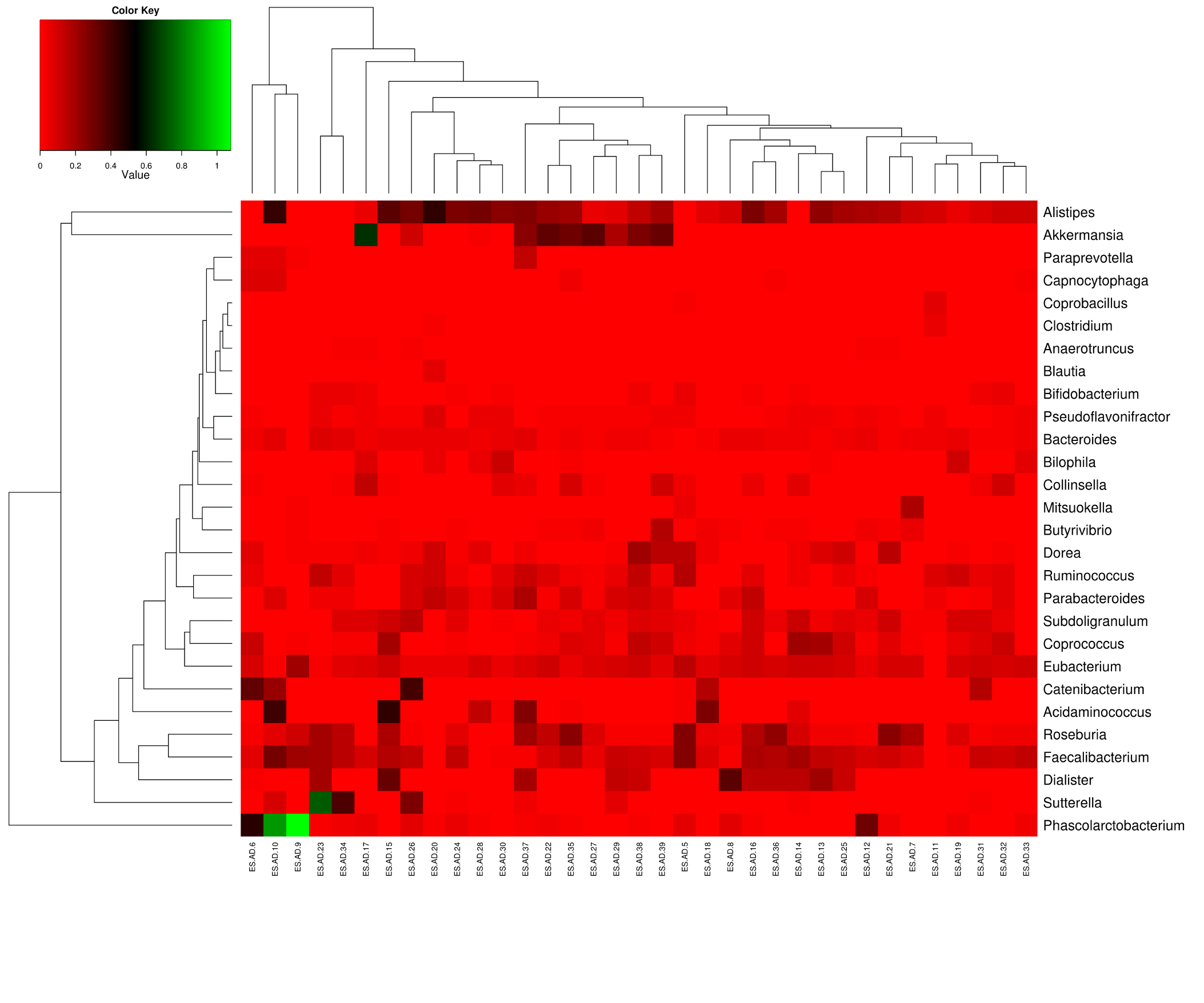

Supplement: Supplementary file 6 — 10.1186/s13099-016-0099-z Heatmap showing the normalized abundances of major genera in the gut microbiomes of Spanish individuals. Only those genera, present in at least 40 % of the individuals with a minimum abundance of 0.05, have been shown in the heatmap. Red color signifies that the genus is either absent or present in low abundance, whereas the green color signifies that it is highly abundant. [file 13099_2016_99_MOESM6_ESM.tif]

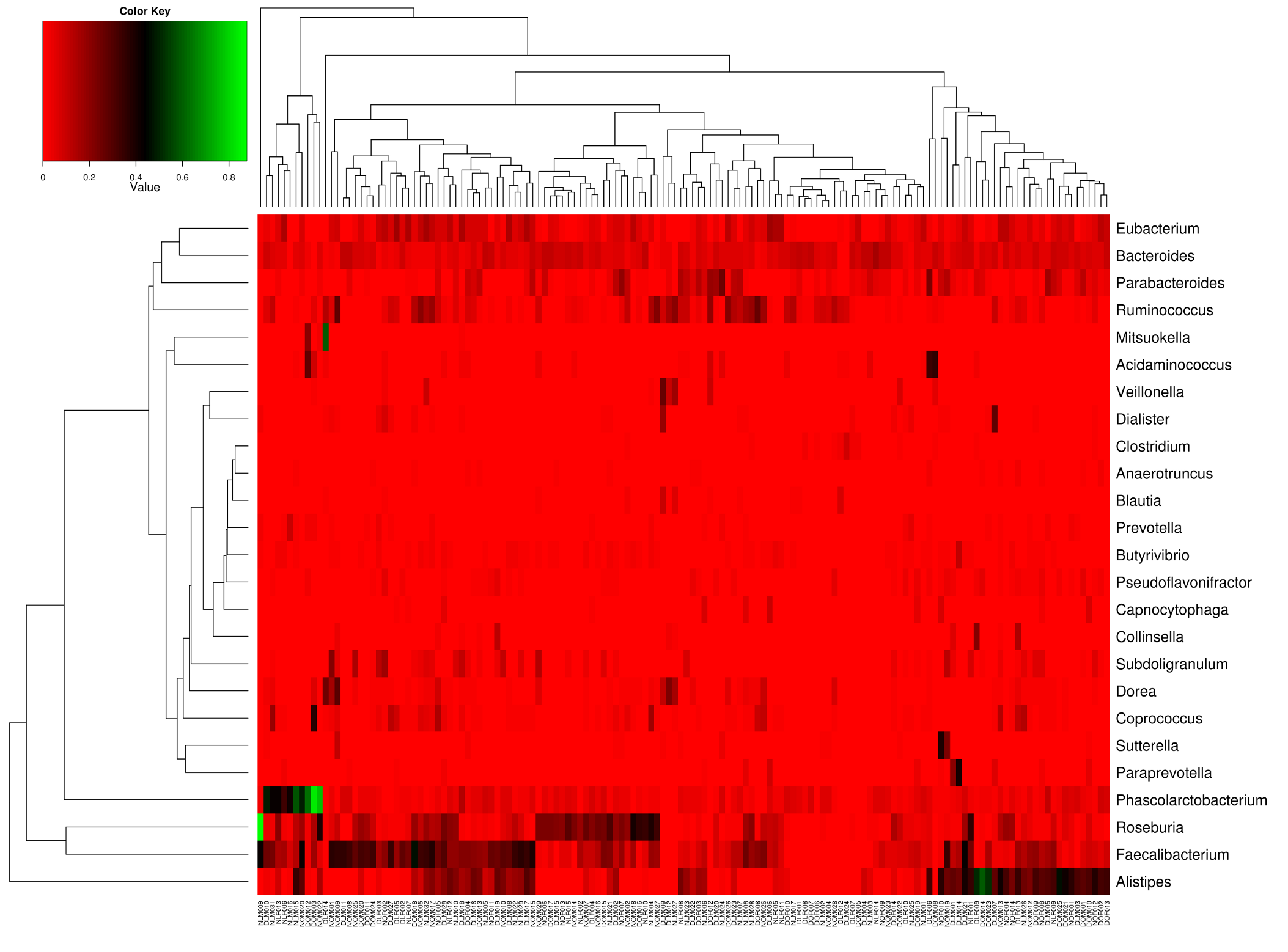

Supplement: Supplementary file 8 — 10.1186/s13099-016-0099-z Heatmap showing the normalized abundances of major genera in the gut microbiomes of Chinese individuals. Only those genera, present in at least 40 % of the individuals with a minimum abundance of 0.05, have been shown in the heatmap. Red color signifies that the genus is either absent or present in low abundance, whereas the green color signifies that it is highly abundant. [file 13099_2016_99_MOESM8_ESM.tif]

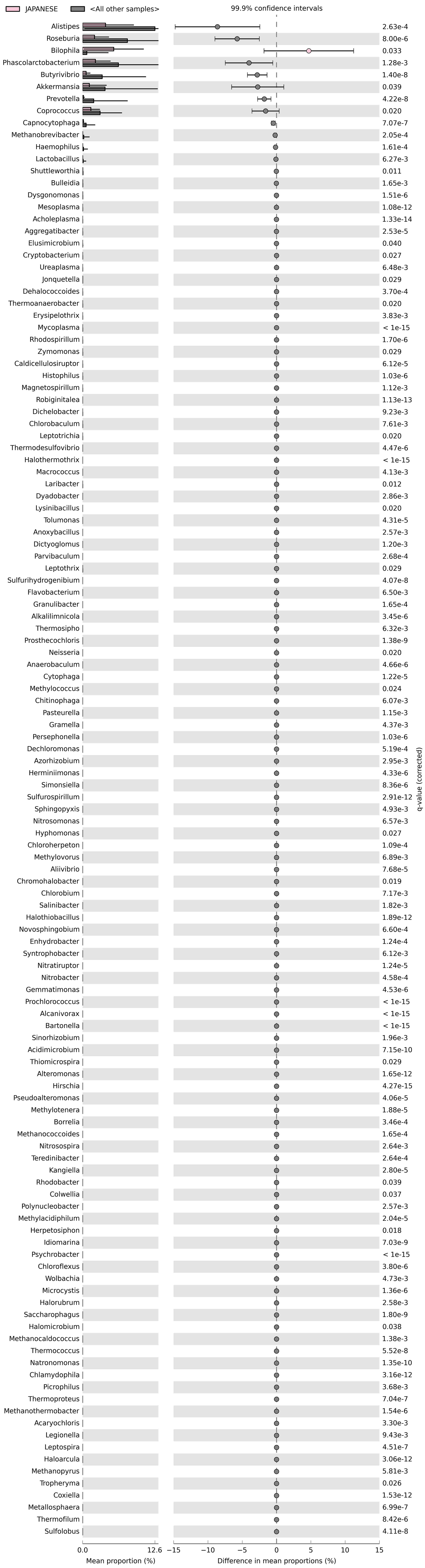

Supplement: Supplementary file 9 — 10.1186/s13099-016-0099-z Significantly over/under-represented genera in the gut microbiomes of the Japanese Individuals as compared to those of the others. Significantly different genera were identified using Welch’s t-test with P-value < 0.05, corrected using Benjamini-Hochberg FDR method for multiple test corrections. Further stringency was established using mean ratio of mean proportions to be 1.5. All tests were performed using the STAMP analysis package. [file 13099_2016_99_MOESM9_ESM.tif]

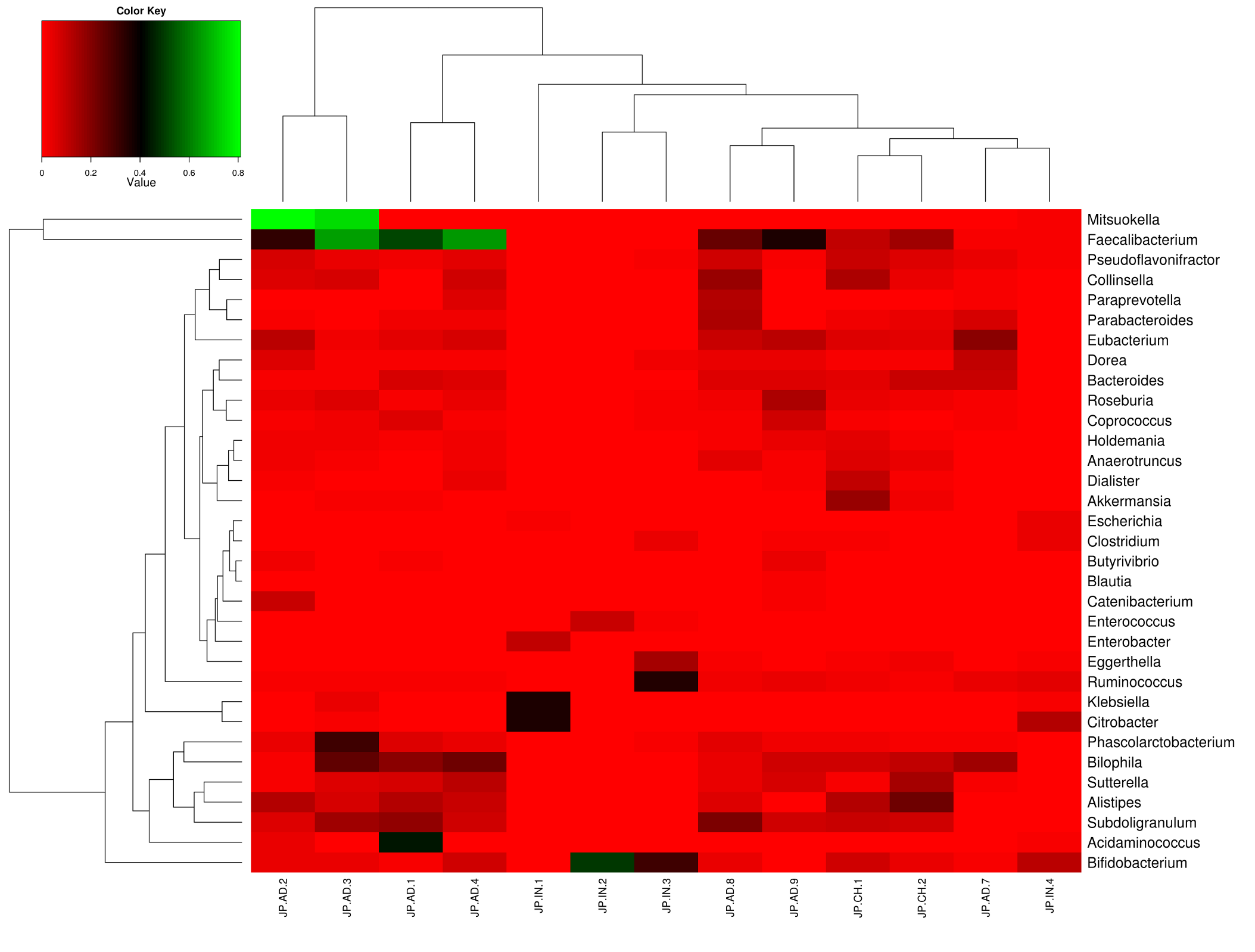

Supplement: Supplementary file 10 — 10.1186/s13099-016-0099-z Heatmap showing the normalized abundances of major genera in the gut microbiomes of Japanese individuals. Only those genera, present in at least 40 % of the individuals with a minimum abundance of 0.05, have been shown in the heatmap. Red color signifies that the genus is either absent or present in low abundance, whereas the green color signifies that it is highly abundant. [file 13099_2016_99_MOESM10_ESM.tif]

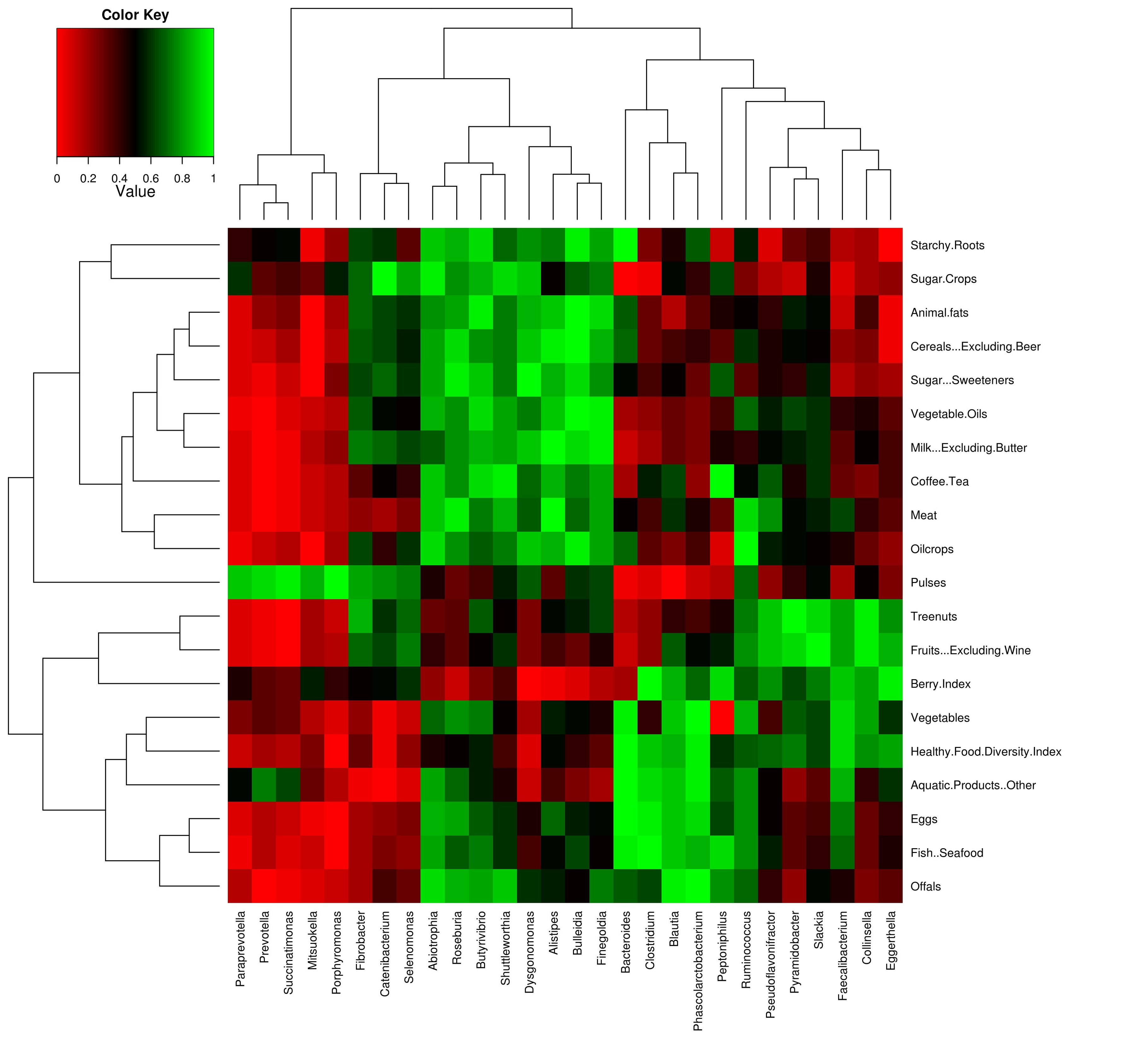

Supplement: Supplementary file 15 — 10.1186/s13099-016-0099-z Heatmap showing the rank-normalized PLS coefficients of association of the nation specific per-capita intakes of the various food components and the food diversity indices (Berry Index and HFD) with the median abundances of the various genera in the corresponding gut microbiomes. Red color signifies low coefficient of association between the per-capita intakes of food components and the genera whereas the green color signifies a high coefficient. [file 13099_2016_99_MOESM15_ESM.tif]

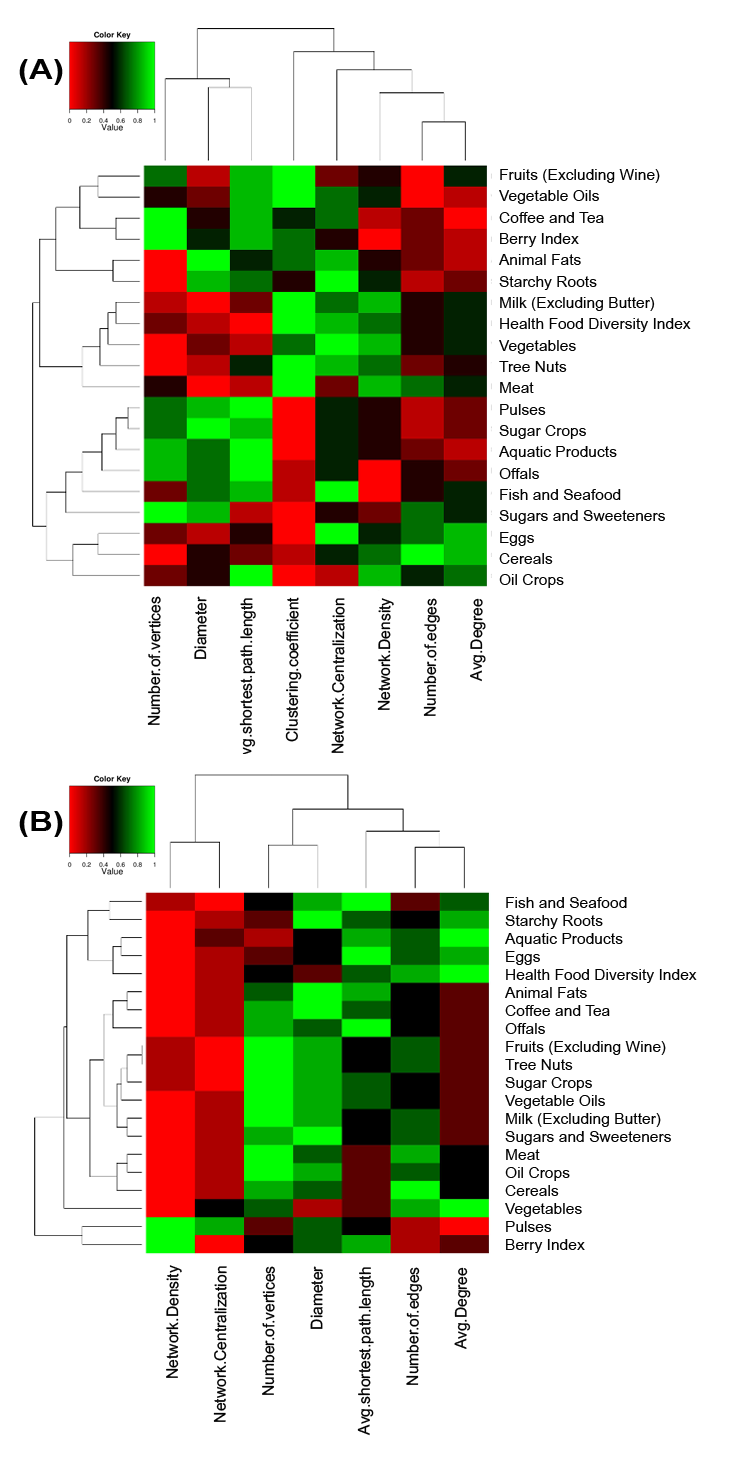

Supplement: Supplementary file 16 — 10.1186/s13099-016-0099-z Heatmap showing the rank-normalized PLS coefficients of association of the nation specific per-capita intakes of the various food components and the food diversity indices (Berry Index and HFD) with the network properties of the (a) co-occurrence and (b) mutual exclusion networks observed for the gut microbiomes of the corresponding nationalities. Red color signifies low coefficient of association between the per-capita intakes of food components and the network properties whereas the green color signifies a high coefficient. [file 13099_2016_99_MOESM16_ESM.tif]

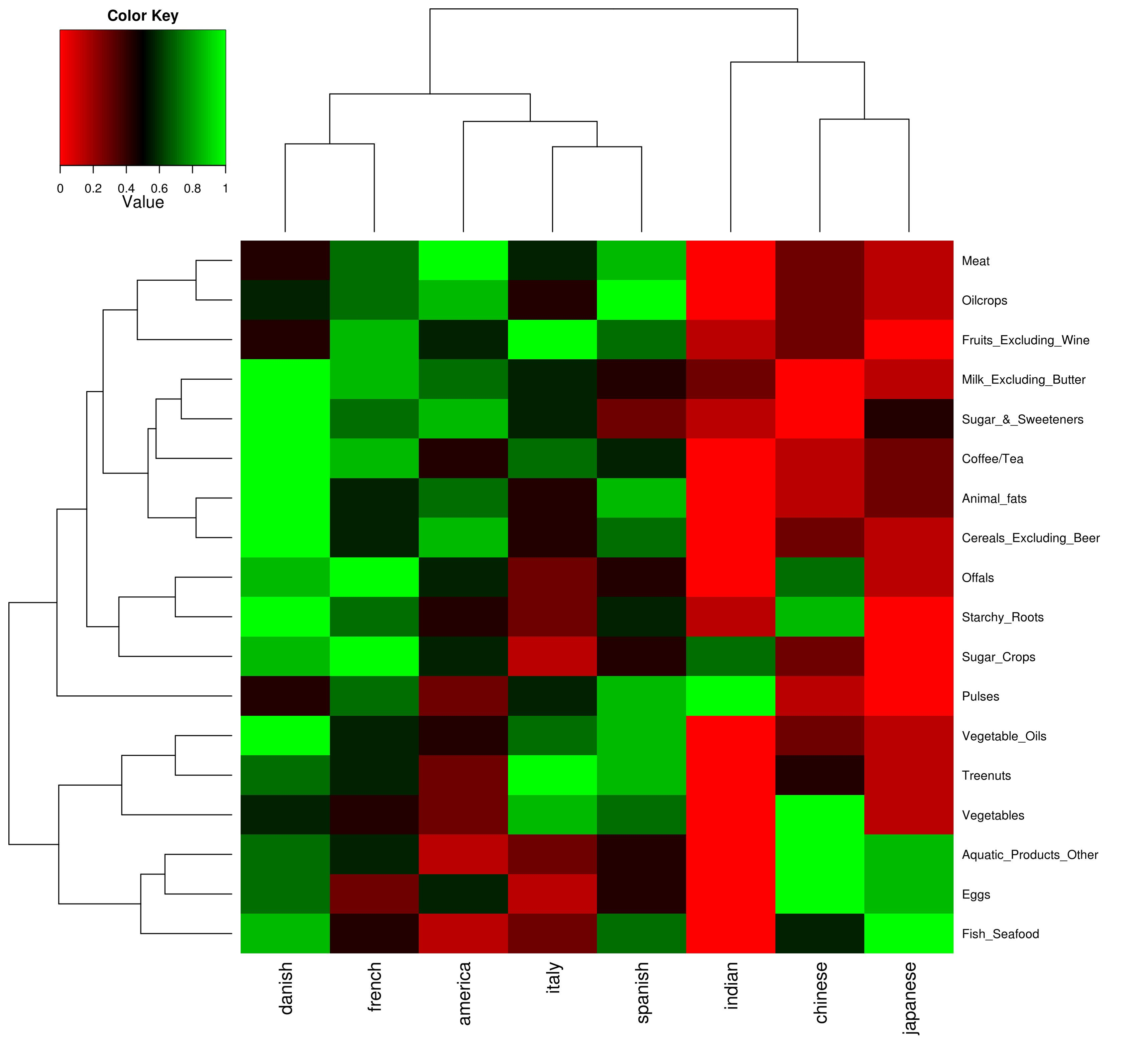

Supplement: Supplementary file 17 — 10.1186/s13099-016-0099-z Heatmap showing the rank normalized median of per-capita intakes of the various food components across the different nationalities. Red color signifies low per-capita intakes of the food component whereas the green color signifies a high per-capita intakes. [file 13099_2016_99_MOESM17_ESM.tif]
