# Supplementary material for: Global investigation of composition and interaction networks in gut microbiomes of individuals belonging to diverse geographies and age-groups
Source: Gut Pathog. 2016 May 6;8:17. doi: 10.1186/s13099-016-0099-z (PMC4858888; doi:10.1186/s13099-016-0099-z)
Supplement: Supplementary file 4 — 10.1186/s13099-016-0099-z Centrality measures of different genera in the gut microbiomes of American, China, Denmark, France, India, Italy, Japan and Spain for co-occurrence and mutual exclusion networks. [file 13099_2016_99_MOESM4_ESM.pdf]

Additional File 4: Centrality measures of different genera in the gut microbiomes of America, China, Denmark, France, India, Italy, Japan, Spain for co-occurrence and mutual exclusion networks

Co-occurrence network

Node_Index	Label	Degree	Cluster_Coeff	Closeness	Betweenness	Eigen_Vector	PageRank	HubScore
1	Abiotrophia	32	0.556	0.533	0.163	0.120	0.017	0.120
2	Acholeplasma	38	0.686	0.593	0.218	0.162	0.019	0.162
3	Alkaliphilus	44	0.631	0.640	0.205	0.183	0.021	0.183
4	Anaerococcus	30	0.789	0.553	0.032	0.132	0.015	0.132
5	Anaerofustis	37	0.592	0.579	0.165	0.148	0.019	0.148
6	Anaerostipes	19	0.626	0.490	0.213	0.073	0.011	0.073
7	Atopobium	42	0.632	0.608	0.140	0.176	0.021	0.176
8	Bifidobacterium	28	0.685	0.545	0.055	0.117	0.014	0.117
9	Bulleidia	8	0.786	0.429	0.002	0.031	0.006	0.031
10	Butyrivibrio	10	0.622	0.451	0.231	0.032	0.009	0.032
11	Caldicellulosiruptor	33	0.782	0.566	0.038	0.149	0.016	0.149
12	Catenibacterium	36	0.643	0.579	0.148	0.149	0.018	0.149
13	Collinsella	37	0.692	0.584	0.091	0.158	0.018	0.158
14	Coprobacillus	6	0.733	0.432	0.002	0.023	0.005	0.023
15	Coprococcus	31	0.630	0.557	0.107	0.126	0.016	0.126
16	Dorea	21	0.614	0.493	0.050	0.075	0.012	0.075
17	Eubacterium	4	1.000	0.415	0.000	0.014	0.004	0.014
18	Finegoldia	22	0.857	0.514	0.016	0.094	0.012	0.094
19	Gemella	36	0.706	0.589	0.086	0.155	0.018	0.155
20	Mesoplasma	37	0.743	0.589	0.057	0.165	0.018	0.165
21	Oribacterium	44	0.605	0.624	0.334	0.178	0.022	0.178
22	Pelotomaculum	44	0.646	0.629	0.212	0.186	0.022	0.186
23	Peptoniphilus	26	0.643	0.500	0.079	0.100	0.014	0.100
24	Peptostreptococcus	29	0.754	0.541	0.063	0.133	0.015	0.133
25	Ruminococcus	12	0.697	0.448	0.079	0.039	0.008	0.039
26	Shuttleworthia	35	0.731	0.566	0.053	0.157	0.017	0.157
27	Solobacterium	36	0.563	0.579	0.223	0.139	0.019	0.139
28	Streptobacillus	23	0.798	0.507	0.025	0.100	0.012	0.100
29	Subdoligranulum	20	0.684	0.510	0.041	0.080	0.011	0.080
30	Thermanaerovibrio	41	0.685	0.624	0.146	0.178	0.020	0.178
31	Thermoanaerobacter	42	0.641	0.613	0.186	0.175	0.021	0.175
32	Treponema	43	0.657	0.624	0.145	0.183	0.021	0.183
33	Turicibacter	35	0.709	0.584	0.101	0.150	0.017	0.150
34	Acetivibrio	17	0.934	0.497	0.006	0.073	0.009	0.073
35	Fusobacterium	19	0.836	0.510	0.026	0.079	0.010	0.079
36	Oxalobacter	21	0.743	0.533	0.941	0.089	0.013	0.089
37	Aerococcus	24	0.725	0.514	0.023	0.107	0.012	0.107
38	Akkermansia	3	1.000	0.401	0.000	0.015	0.003	0.015
39	Alicyclobacillus	33	0.822	0.557	0.018	0.156	0.016	0.156
40	Fibrobacter	33	0.748	0.593	0.436	0.149	0.017	0.149
41	Mitsuokella	35	0.790	0.566	0.024	0.162	0.017	0.162
42	Moorella	37	0.728	0.584	0.057	0.164	0.018	0.164
43	Parascardovia	39	0.699	0.629	0.648	0.170	0.020	0.170
44	Pseudoramibacter	39	0.677	0.593	0.108	0.166	0.019	0.166
45	Pyramidobacter	39	0.717	0.603	0.094	0.172	0.019	0.172
46	Selenomonas	40	0.728	0.608	0.076	0.179	0.019	0.179
47	Slackia	41	0.694	0.613	0.112	0.179	0.020	0.179
48	Symbiobacterium	35	0.714	0.566	0.081	0.152	0.017	0.152
49	Acidaminococcus	1	0.000	0.260	0.000	0.000	0.003	0.000
50	Blautia	5	0.300	0.349	0.237	0.005	0.008	0.005

51	Bilophila	25	0.763	0.521	0.036	0.110	0.013	0.110
52	Dehalococcoides	13	0.705	0.462	0.007	0.057	0.008	0.057
53	Desulfitobacterium	35	0.630	0.575	0.199	0.142	0.018	0.142
54	Eggerthella	30	0.724	0.541	0.108	0.125	0.016	0.125
55	Heliobacterium	31	0.746	0.549	0.092	0.134	0.016	0.134
56	Holdemania	28	0.743	0.545	0.205	0.119	0.015	0.119
57	Jonquetella	23	0.874	0.518	0.013	0.106	0.012	0.106
58	Alistipes	2	0.000	0.317	0.167	0.000	0.006	0.000
59	Bacteroides	4	0.500	0.251	0.011	1.99E-05	0.010	1.99E-05
60	Dyadobacter	7	0.286	0.448	1.000	0.013	0.011	0.013
61	Clostridium	16	0.583	0.480	0.163	0.057	0.010	0.057
62	Succinatimonas	8	0.786	0.440	0.002	0.035	0.006	0.035
63	Anaerotruncus	3	0.333	0.369	0.017	0.006	0.004	0.006
64	Pseudoflavonifractor	16	0.825	0.456	0.049	0.064	0.010	0.064
65	Capnocytophaga	6	0.600	0.253	0.011	3.85E-05	0.014	3.85E-05
66	Parabacteroides	6	0.733	0.284	0.032	0.000	0.013	0.000
67	Paraprevotella	6	0.733	0.284	0.032	0.000	0.013	0.000
68	Faecalibacterium	6	0.867	0.412	0.000	0.024	0.005	0.024
69	Roseburia	1	0.000	0.312	0.000	0.001	0.003	0.001
70	Dysgonomonas	7	0.571	0.326	0.140	0.000	0.014	0.000
71	Prevotella	5	0.900	0.282	0.010	0.000	0.011	0.000
72	Sphingobacterium	3	0.667	0.320	0.084	0.000	0.007	0.000
73	Mobiluncus	3	1.000	0.384	0.000	0.012	0.003	0.012
74	Porphyromonas	6	0.533	0.376	0.665	0.003	0.011	0.003

Mutual exclusion network

Node_Index	Label	Degree	Cluster_Coeff	Closeness	Betweenness	Eigen_Vector	PageRank	HubScore
1	Abiotrophia	1	0.000	0.233	0.000	0.096	0.007	0.096
2	Bacteroides	46	0.000	0.299	1.000	0.672	0.260	0.672
3	Acholeplasma	2	0.000	0.242	0.014	0.112	0.011	0.112
4	Blautia	9	0.000	0.220	0.099	0.119	0.043	0.119
5	Acidaminococcus	1	0.000	0.156	0.000	0.002	0.008	0.002
6	Dyadobacter	2	0.000	0.183	0.036	0.017	0.013	0.017
7	Alicyclobacillus	1	0.000	0.233	0.000	0.096	0.007	0.096
8	Alistipes	2	0.000	0.201	0.000	0.042	0.010	0.042
9	Anaerostipes	5	0.000	0.244	0.041	0.146	0.023	0.146
10	Ruminococcus	6	0.000	0.246	0.077	0.149	0.028	0.149
11	Alkaliphilus	1	0.000	0.233	0.000	0.096	0.007	0.096
12	Anaerococcus	2	0.000	0.242	0.014	0.112	0.011	0.112
13	Anaerofustis	4	0.000	0.242	0.024	0.140	0.019	0.140
14	Dysgonomonas	6	0.000	0.215	0.045	0.101	0.028	0.101
15	Parabacteroides	5	0.000	0.204	0.004	0.097	0.023	0.097
16	Paraprevotella	6	0.000	0.217	0.018	0.116	0.026	0.116
17	Anaerotruncus	2	0.000	0.203	0.001	0.022	0.011	0.022
18	Finegoldia	3	0.000	0.244	0.044	0.116	0.016	0.116
19	Prevotella	4	0.000	0.211	0.026	0.039	0.020	0.039
20	Atopobium	1	0.000	0.233	0.000	0.096	0.007	0.096
21	Bifidobacterium	1	0.000	0.233	0.000	0.096	0.007	0.096
22	Bilophila	1	0.000	0.233	0.000	0.096	0.007	0.096
23	Butyrivibrio	1	0.000	0.233	0.000	0.096	0.007	0.096
24	Catenibacterium	1	0.000	0.233	0.000	0.096	0.007	0.096

25	Clostridium	4	0.000	0.242	0.024	0.140	0.019	0.140
26	Collinsella	1	0.000	0.233	0.000	0.096	0.007	0.096
27	Coprococcus	2	0.000	0.234	0.006	0.109	0.011	0.109
28	Desulfitobacterium	2	0.000	0.236	0.039	0.098	0.012	0.098
29	Dorea	1	0.000	0.233	0.000	0.096	0.007	0.096
30	Eggerthella	1	0.000	0.233	0.000	0.096	0.007	0.096
31	Fibrobacter	1	0.000	0.233	0.000	0.096	0.007	0.096
32	Gemella	1	0.000	0.233	0.000	0.096	0.007	0.096
33	Heliobacterium	1	0.000	0.233	0.000	0.096	0.007	0.096
34	Holdemania	1	0.000	0.233	0.000	0.096	0.007	0.096
35	Jonquetella	1	0.000	0.233	0.000	0.096	0.007	0.096
36	Mesoplasma	2	0.000	0.236	0.035	0.100	0.013	0.100
37	Mitsuokella	1	0.000	0.233	0.000	0.096	0.007	0.096
38	Moorella	1	0.000	0.233	0.000	0.096	0.007	0.096
39	Oribacterium	1	0.000	0.233	0.000	0.096	0.007	0.096
40	Parascardovia	1	0.000	0.233	0.000	0.096	0.007	0.096
41	Pelotomaculum	1	0.000	0.233	0.000	0.096	0.007	0.096
42	Peptoniphilus	2	0.000	0.242	0.014	0.112	0.011	0.112
43	Pseudoramibacter	1	0.000	0.233	0.000	0.096	0.007	0.096
44	Pyramidobacter	1	0.000	0.233	0.000	0.096	0.007	0.096
45	Selenomonas	1	0.000	0.233	0.000	0.096	0.007	0.096
46	Shuttleworthia	1	0.000	0.233	0.000	0.096	0.007	0.096
47	Slackia	2	0.000	0.236	0.035	0.100	0.013	0.100
48	Solobacterium	1	0.000	0.233	0.000	0.096	0.007	0.096
49	Streptobacillus	2	0.000	0.242	0.014	0.112	0.011	0.112
50	Subdoligranulum	4	0.000	0.243	0.081	0.120	0.020	0.120
51	Succinatimonas	1	0.000	0.233	0.000	0.096	0.007	0.096
52	Symbiobacterium	1	0.000	0.233	0.000	0.096	0.007	0.096
53	Thermanaerovibrio	1	0.000	0.233	0.000	0.096	0.007	0.096
54	Thermoanaerobacter	1	0.000	0.233	0.000	0.096	0.007	0.096
55	Treponema	1	0.000	0.233	0.000	0.096	0.007	0.096
56	Turicibacter	1	0.000	0.233	0.000	0.096	0.007	0.096
57	Capnocytophaga	2	0.000	0.200	0.009	0.019	0.011	0.019
58	Pseudoflavonifractor	3	0.000	0.179	0.006	0.010	0.017	0.010
59	Coprobacillus	1	0.000	0.179	0.000	0.014	0.006	0.014
60	Sutterella	2	0.000	0.199	0.009	0.015	0.012	0.015
61	Dialister	1	0.000	0.016	0.000	1.84E-18	0.015	0.000
62	Phascolarctobacterium	1	0.000	0.016	0.000	0.000	0.015	0.000
63	Megasphaera	3	0.000	0.195	0.036	0.029	0.020	0.029
64	Oxalobacter	1	0.000	0.165	0.000	0.004	0.008	0.004
65	Sphingobacterium	1	0.000	0.199	0.000	0.021	0.006	0.021

Co-occurrence network

Node_Index	Label	Degree	Cluster_Coeff	Closeness	Betweenness	Eigen_Vector	PageRank	HubScore
1	Abiotrophia	14	0.626	0.482	0.042	0.114	0.019	0.114
2	Anaerofustis	18	0.569	0.505	0.067	0.176	0.022	0.176
3	Anaerostipes	15	0.619	0.486	0.037	0.124	0.019	0.124
4	Butyrivibrio	12	0.621	0.453	0.025	0.098	0.016	0.098
5	Catenibacterium	18	0.601	0.505	0.061	0.169	0.023	0.169
6	Clostridium	9	0.833	0.421	0.003	0.070	0.013	0.070
7	Coprococcus	25	0.480	0.541	0.238	0.210	0.031	0.210
8	Dorea	17	0.588	0.495	0.056	0.132	0.022	0.132
9	Eubacterium	10	0.667	0.482	0.109	0.080	0.015	0.080
10	Oribacterium	20	0.532	0.525	0.141	0.190	0.025	0.190
11	Peptoniphilus	21	0.452	0.530	0.299	0.159	0.027	0.159
12	Peptostreptococcus	12	0.652	0.473	0.017	0.118	0.016	0.118
13	Roseburia	5	0.900	0.390	0.001	0.032	0.009	0.032
14	Shuttleworthia	29	0.463	0.589	0.265	0.261	0.035	0.261
15	Subdoligranulum	23	0.545	0.582	0.236	0.219	0.029	0.219
16	Acidaminococcus	3	0.333	0.333	0.355	0.004	0.015	0.004
17	Dialister	8	0.500	0.477	0.534	0.067	0.015	0.067
18	Escherichia	2	1.000	0.252	0.000	0.000	0.012	0.000
19	Shigella	2	1.000	0.252	0.000	0.000	0.012	0.000
20	Alistipes	18	0.366	0.541	1.000	0.125	0.032	0.125
21	Anaerotruncus	13	0.885	0.525	0.017	0.149	0.017	0.149
22	Bilophila	5	0.400	0.398	0.150	0.018	0.015	0.018
23	Capnocytophaga	3	1.000	0.363	0.000	0.009	0.009	0.009
24	Collinsella	24	0.569	0.589	0.195	0.236	0.030	0.236
25	Dysgonomonas	4	0.500	0.411	0.018	0.021	0.010	0.021
26	Heliobacterium	17	0.691	0.546	0.252	0.168	0.024	0.168
27	Holdemania	17	0.706	0.546	0.075	0.178	0.022	0.178
28	Parabacteroides	4	0.333	0.366	0.224	0.008	0.016	0.008
29	Paraprevotella	5	0.600	0.368	0.037	0.010	0.014	0.010
30	Porphyromonas	4	0.667	0.366	0.005	0.010	0.011	0.010
31	Prevotella	7	0.381	0.424	0.190	0.026	0.017	0.026
32	Pseudoflavonifractor	23	0.573	0.582	0.247	0.226	0.029	0.226
33	Pseudoramibacter	26	0.529	0.624	0.298	0.248	0.032	0.248
34	Pyramidobacter	24	0.572	0.609	0.223	0.234	0.030	0.234
35	Selenomonas	20	0.605	0.582	0.297	0.192	0.027	0.192
36	Slackia	20	0.679	0.558	0.099	0.205	0.025	0.205
37	Anaerococcus	10	0.800	0.453	0.006	0.087	0.014	0.087
38	Atopobium	18	0.588	0.515	0.073	0.171	0.023	0.171
39	Bulleidia	12	0.712	0.465	0.019	0.121	0.016	0.121
40	Finegoldia	10	0.644	0.431	0.012	0.084	0.014	0.084
41	Solobacterium	24	0.504	0.546	0.158	0.217	0.029	0.217
42	Streptobacillus	10	0.778	0.457	0.007	0.092	0.014	0.092
43	Turicibacter	9	0.861	0.424	0.003	0.077	0.013	0.077
44	Eggerthella	16	0.742	0.510	0.022	0.172	0.020	0.172
45	Blautia	8	0.893	0.414	0.001	0.061	0.012	0.061
46	Coprobacillus	14	0.615	0.486	0.045	0.133	0.018	0.133
47	Ruminococcus	9	0.889	0.417	0.002	0.070	0.013	0.070
48	Symbiobacterium	17	0.728	0.505	0.039	0.179	0.021	0.179
49	Bacteroides	2	1.000	0.290	0.000	0.001	0.009	0.001
50	Faecalibacterium	8	0.679	0.424	0.012	0.066	0.012	0.066
51	Megasphaera	7	0.857	0.442	0.006	0.072	0.011	0.072

52	Mitsuokella	12	0.697	0.505	0.084	0.116	0.018	0.116
53	Sutterella	1	0.000	0.269	0.000	0.000	0.006	0.000
54	Phascolarctobacterium	2	1.000	0.301	0.000	0.002	0.007	0.002

Mutual exclusion network

Node_Index	Label	Degree	Cluster_Coeff	Closeness	Betweenness	Eigen_Vector	PageRank	HubScore
1	Abiotrophia	6	0.000	0.454	0.151	0.244	0.037	0.304
2	Bacteroides	23	0.000	0.611	1.000	0.534	0.166	0.000
3	Bilophila	2	0.000	0.324	0.001	0.090	0.014	0.000
4	Escherichia	9	0.000	0.379	0.144	0.261	0.051	0.000
5	Parabacteroides	7	0.000	0.373	0.064	0.236	0.043	0.000
6	Shigella	7	0.000	0.367	0.080	0.209	0.039	0.000
7	Sutterella	1	0.000	0.314	0.000	0.043	0.009	0.000
8	Acidaminococcus	5	0.000	0.349	0.034	0.151	0.029	0.000
9	Butyrivibrio	5	0.000	0.444	0.097	0.247	0.029	0.308
10	Coprococcus	6	0.000	0.454	0.127	0.263	0.035	0.328
11	Eubacterium	3	0.000	0.284	0.002	0.110	0.018	0.137
12	Faecalibacterium	4	0.000	0.295	0.037	0.119	0.025	0.149
13	Roseburia	3	0.000	0.284	0.002	0.110	0.018	0.137
14	Alistipes	2	0.000	0.319	0.003	0.053	0.016	0.000
15	Clostridium	4	0.000	0.404	0.063	0.156	0.028	0.194
16	Ruminococcus	8	0.000	0.444	0.302	0.145	0.063	0.180
17	Anaerococcus	1	0.000	0.383	0.000	0.095	0.009	0.118
18	Anaerofustis	1	0.000	0.383	0.000	0.095	0.009	0.118
19	Anaerostipes	2	0.000	0.389	0.024	0.103	0.017	0.128
20	Paraprevotella	2	0.000	0.314	0.005	0.044	0.017	0.000
21	Anaerotruncus	2	0.000	0.280	0.000	0.083	0.013	0.104
22	Atopobium	1	0.000	0.246	0.000	0.009	0.010	0.012
23	Phascolarctobacterium	3	0.000	0.324	0.066	0.053	0.025	0.000
24	Blautia	2	0.000	0.389	0.004	0.137	0.015	0.170
25	Catenibacterium	1	0.000	0.383	0.000	0.095	0.009	0.118
26	Collinsella	1	0.000	0.383	0.000	0.095	0.009	0.118
27	Dialister	1	0.000	0.383	0.000	0.095	0.009	0.118
28	Dorea	2	0.000	0.389	0.004	0.137	0.015	0.170
29	Mitsuokella	1	0.000	0.383	0.000	0.095	0.009	0.118
30	Oribacterium	1	0.000	0.383	0.000	0.095	0.009	0.118
31	Peptoniphilus	3	0.000	0.404	0.061	0.146	0.022	0.182
32	Peptostreptococcus	1	0.000	0.383	0.000	0.095	0.009	0.118
33	Pseudoramibacter	1	0.000	0.383	0.000	0.095	0.009	0.118
34	Pyramidobacter	1	0.000	0.383	0.000	0.095	0.009	0.118
35	Selenomonas	1	0.000	0.383	0.000	0.095	0.009	0.118
36	Shuttleworthia	2	0.000	0.419	0.021	0.141	0.014	0.176
37	Solobacterium	1	0.000	0.383	0.000	0.095	0.009	0.118
38	Subdoligranulum	3	0.000	0.444	0.160	0.150	0.021	0.187
39	Capnocytophaga	1	0.000	0.310	0.000	0.026	0.010	0.000
40	Prevotella	2	0.000	0.319	0.003	0.053	0.016	0.000
41	Dysgonomonas	1	0.000	0.310	0.000	0.026	0.010	0.000
42	Fusobacterium	4	0.000	0.333	0.125	0.051	0.032	0.000
43	Holdemania	1	0.000	0.251	0.000	0.009	0.010	0.011
44	Pseudoflavonifractor	1	0.000	0.251	0.000	0.009	0.010	0.011
45	Porphyromonas	1	0.000	0.310	0.000	0.026	0.010	0.000

Co-occurrence network

Node_Index	Label	Degree	Cluster_Coeff	Closeness	Betweenness	Eigen_Vector	PageRank	HubScore
1	Abiotrophia	1	0.000	0.125	0.000	0.009	0.003	0.009
2	Anaerococcus	22	0.784	0.141	0.190	0.184	0.020	0.184
3	Acetivibrio	22	0.823	0.142	0.020	0.191	0.018	0.191
4	Acholeplasma	27	0.638	0.143	0.179	0.212	0.023	0.212
5	Alicyclobacillus	24	0.500	0.145	0.280	0.153	0.021	0.153
6	Alkaliphilus	30	0.579	0.145	0.197	0.227	0.025	0.227
7	Anaerofustis	21	0.795	0.142	0.075	0.181	0.018	0.181
8	Brachyspira	19	0.801	0.139	0.028	0.161	0.016	0.161
9	Caldicellulosiruptor	24	0.754	0.142	0.035	0.203	0.020	0.203
10	Erysipelothrix	25	0.730	0.143	0.049	0.212	0.021	0.212
11	Finegoldia	23	0.719	0.145	0.194	0.192	0.019	0.192
12	Fusobacterium	26	0.643	0.143	0.116	0.202	0.022	0.202
13	Gemella	18	0.948	0.141	0.005	0.167	0.015	0.167
14	Granulicatella	26	0.708	0.143	0.056	0.216	0.021	0.216
15	Leptotrichia	17	0.926	0.137	0.006	0.153	0.015	0.153
16	Mesoplasma	27	0.613	0.144	0.990	0.208	0.025	0.208
17	Mycoplasma	32	0.567	0.148	0.249	0.241	0.026	0.241
18	Peptoniphilus	21	0.705	0.142	0.234	0.173	0.019	0.173
19	Selenomonas	31	0.467	0.147	0.535	0.198	0.027	0.198
20	Solobacterium	16	0.900	0.140	0.005	0.149	0.014	0.149
21	Streptobacillus	21	0.752	0.140	0.065	0.171	0.019	0.171
22	Syntrophomonas	12	0.652	0.138	0.046	0.094	0.011	0.094
23	Thermoanaerobacter	22	0.814	0.141	0.027	0.189	0.018	0.189
24	Turicibacter	27	0.661	0.144	0.139	0.218	0.023	0.218
25	Aerococcus	13	0.654	0.136	0.023	0.102	0.012	0.102
26	Bulleidia	3	1.000	0.129	0.000	0.028	0.004	0.028
27	Heliobacterium	24	0.496	0.143	0.343	0.137	0.023	0.137
28	Jonquetella	25	0.540	0.146	0.315	0.182	0.022	0.182
29	Peptostreptococcus	3	1.000	0.128	0.000	0.028	0.004	0.028
30	Treponema	12	0.545	0.138	0.057	0.088	0.012	0.088
31	Acidaminococcus	7	1.000	0.134	0.000	0.033	0.008	0.033
32	Eggerthella	16	0.692	0.140	0.153	0.068	0.017	0.068
33	Mitsuokella	10	0.933	0.136	0.002	0.050	0.011	0.050
34	Pseudoramibacter	19	0.614	0.141	0.118	0.096	0.018	0.096
35	Pyramidobacter	20	0.547	0.143	0.357	0.088	0.020	0.088
36	Slackia	18	0.641	0.141	0.225	0.078	0.019	0.078
37	Magnetospirillum	7	0.667	0.133	0.004	0.045	0.008	0.045
38	Rhodospirillum	5	0.800	0.131	0.001	0.030	0.006	0.030
39	Akkermansia	15	0.562	0.140	0.146	0.106	0.014	0.106
40	Bifidobacterium	3	0.333	0.128	0.002	0.009	0.005	0.009
41	Thermanaerovibrio	22	0.524	0.142	0.143	0.124	0.020	0.124
42	Desulfovibrio	11	0.564	0.138	0.065	0.066	0.011	0.066
43	Parascardovia	17	0.669	0.142	0.340	0.087	0.017	0.087
44	Pseudoflavonifractor	15	0.686	0.140	0.225	0.068	0.016	0.068
45	Shuttleworthia	14	0.714	0.139	0.049	0.069	0.014	0.069
46	Symbiobacterium	20	0.611	0.143	0.168	0.133	0.018	0.133
47	Alistipes	3	0.000	0.119	0.688	0.001	0.014	0.001
48	Anaerotruncus	7	0.476	0.131	1.000	0.018	0.014	0.018
49	Bilophila	2	0.000	0.108	0.351	3.99E-05	0.012	3.99E-05
50	Dysgonomonas	1	0.000	0.107	0.000	3.98E-05	0.006	3.98E-05

51	Fibrobacter	3	0.667	0.129	0.000	0.022	0.005	0.022
52	Dialister	1	0.000	0.117	0.000	0.001	0.004	0.001
53	Atopobium	1	0.000	0.125	0.000	0.008	0.003	0.008
54	Collinsella	6	0.733	0.128	0.007	0.019	0.008	0.019
55	Parabacteroides	2	0.000	0.099	0.178	1.86E-06	0.014	1.86E-06
56	Blautia	1	0.000	0.014	0.000	0.000	0.014	0.000
57	Roseburia	1	0.000	0.014	0.000	5.08E-19	0.014	0.000
58	Butyrivibrio	2	0.000	0.129	0.680	0.010	0.007	0.010
59	Prevotella	4	0.167	0.117	0.524	0.000	0.020	0.000
60	Capnocytophaga	2	1.000	0.106	0.000	2.23E-05	0.011	2.23E-05
61	Paraprevotella	2	1.000	0.106	0.000	2.23E-05	0.011	2.23E-05
62	Coprobacillus	1	0.000	0.015	0.000	0.000	0.011	0.000
63	Eubacterium	2	0.000	0.015	0.003	0.000	0.021	0.000
64	Coprococcus	1	0.000	0.122	0.000	0.002	0.003	0.002
65	Desulfitobacterium	9	0.639	0.137	0.181	0.046	0.011	0.046
66	Dehalococcoides	1	0.000	0.127	0.000	0.006	0.003	0.006
67	Dorea	1	0.000	0.015	0.000	0.000	0.011	0.000
68	Faecalibacterium	1	0.000	0.090	0.000	8.70E-08	0.008	8.70E-08
69	Holdemania	4	1.000	0.133	0.000	0.022	0.005	0.022
70	Porphyromonas	1	0.000	0.106	0.000	2.13E-05	0.006	2.13E-05

Mutual exclusion network

Node_Index	Label	Degree	Cluster_Coeff	Closeness	Betweenness	Eigen_Vector	PageRank	HubScore
1	Acetivibrio	1	0.000	0.093	0.000	0.079	0.009	0.039
2	Bacteroides	15	0.000	0.100	1.000	0.404	0.105	0.536
3	Acholeplasma	3	0.000	0.100	0.144	0.237	0.021	0.116
4	Faecalibacterium	11	0.000	0.096	0.347	0.326	0.074	0.432
5	Roseburia	18	0.000	0.099	0.883	0.475	0.130	0.631
6	Akkermansia	2	0.000	0.094	0.026	0.157	0.015	0.077
7	Alicyclobacillus	1	0.000	0.092	0.000	0.093	0.009	0.046
8	Alistipes	2	0.000	0.089	0.086	0.019	0.021	0.026
9	Anaerostipes	1	0.000	0.083	0.000	0.004	0.012	0.002
10	Prevotella	5	0.000	0.095	0.549	0.094	0.044	0.046
11	Anaerococcus	2	0.000	0.095	0.028	0.143	0.015	0.070
12	Anaerofustis	1	0.000	0.093	0.000	0.079	0.009	0.039
13	Anaerotruncus	1	0.000	0.021	0.000	0.000	0.020	0.000
14	Coprobacillus	1	0.000	0.021	0.000	0.000	0.020	0.000
15	Atopobium	1	0.000	0.021	0.000	0.000	0.020	0.000
16	Sutterella	1	0.000	0.021	0.000	0.000	0.020	0.000
17	Butyrivibrio	1	0.000	0.093	0.000	0.079	0.009	0.039
18	Finegoldia	2	0.000	0.095	0.028	0.143	0.015	0.070
19	Fusobacterium	2	0.000	0.095	0.028	0.143	0.015	0.070
20	Gemella	2	0.000	0.095	0.028	0.143	0.015	0.070
21	Heliobacterium	2	0.000	0.099	0.090	0.173	0.015	0.085
22	Mesoplasma	3	0.000	0.100	0.144	0.237	0.021	0.116
23	Oribacterium	1	0.000	0.093	0.000	0.079	0.009	0.039
24	Pseudoramibacter	2	0.000	0.099	0.090	0.173	0.015	0.085
25	Selenomonas	2	0.000	0.099	0.090	0.173	0.015	0.085
26	Thermoanaerobacter	1	0.000	0.093	0.000	0.079	0.009	0.039
27	Bifidobacterium	1	0.000	0.089	0.000	0.019	0.011	0.025
28	Blautia	2	0.000	0.088	0.003	0.053	0.016	0.071

29	Symbiobacterium	2	0.000	0.092	0.027	0.104	0.016	0.051
30	Turicibacter	3	0.000	0.095	0.085	0.168	0.022	0.082
31	Desulfitobacterium	1	0.000	0.021	0.000	0.000	0.020	0.000
32	Parabacteroides	1	0.000	0.021	0.000	0.000	0.020	0.000
33	Dialister	1	0.000	0.078	0.000	0.001	0.012	0.001
34	Phascolarctobacterium	2	0.000	0.083	0.086	0.004	0.022	0.002
35	Dysgonomonas	2	0.000	0.089	0.168	0.019	0.020	0.026
36	Eggerthella	1	0.000	0.092	0.000	0.093	0.009	0.046
37	Erysipelothrix	2	0.000	0.094	0.026	0.157	0.015	0.077
38	Peptoniphilus	1	0.000	0.089	0.000	0.064	0.009	0.031
39	Streptobacillus	1	0.000	0.089	0.000	0.064	0.009	0.031
40	Jonquetella	1	0.000	0.092	0.000	0.093	0.009	0.046
41	Mitsuokella	1	0.000	0.021	0.000	3.46E-19	0.020	0.000
42	Ruminococcus	1	0.000	0.021	0.000	1.49E-17	0.020	0.000
43	Mycoplasma	1	0.000	0.092	0.000	0.093	0.009	0.046
44	Parascardovia	1	0.000	0.092	0.000	0.093	0.009	0.046
45	Subdoligranulum	1	0.000	0.089	0.000	0.019	0.011	0.025
46	Pseudoflavonifractor	1	0.000	0.092	0.000	0.093	0.009	0.046
47	Pyramidobacter	1	0.000	0.092	0.000	0.093	0.009	0.046
48	Slackia	1	0.000	0.092	0.000	0.093	0.009	0.046
49	Thermanaerovibrio	1	0.000	0.092	0.000	0.093	0.009	0.046

Co-occurrence network

Node_Index	Label	Degree	Cluster_Coeff	Closeness	Betweenness	Eigen_Vector	PageRank	HubScore
1	Acholeplasma	2	0.000	0.014	0.011	9.34E-18	0.016	5.39E-17
2	Desulfococcus	1	0.000	0.014	0.000	2.69E-18	0.009	1.42E-17
3	Mesoplasma	2	0.000	0.014	0.017	1.57E-17	0.015	5.39E-17
4	Akkermansia	1	0.000	0.013	0.000	0.000	0.013	2.06E-17
5	Bacteroides	1	0.000	0.013	0.000	0.000	0.013	2.06E-17
6	Alicyclobacillus	2	0.000	0.025	0.102	0.021	0.011	0.021
7	Symbiobacterium	1	0.000	0.024	0.000	0.003	0.006	0.003
8	Thioalkalivibrio	3	0.333	0.025	0.199	0.123	0.011	0.123
9	Alkalilimnicola	1	0.000	0.024	0.000	0.000	0.008	0.000
10	Thermoanaerobacter	2	0.000	0.024	0.102	0.001	0.013	0.001
11	Ammonifex	3	0.667	0.025	0.008	0.012	0.011	0.012
12	Desmospora	4	0.333	0.025	0.049	0.026	0.014	0.026
13	Sphaerobacter	3	0.667	0.025	0.008	0.012	0.011	0.012
14	Thermanaerovibrio	5	0.200	0.025	0.251	0.034	0.019	0.034
15	Anaerococcus	1	0.000	0.013	0.000	0.000	0.013	2.06E-17
16	Peptoniphilus	1	0.000	0.013	0.000	2.38E-18	0.013	2.06E-17
17	Anaerostipes	2	1.000	0.014	0.000	9.71E-18	0.013	1.57E-17
18	Blautia	2	1.000	0.014	0.000	2.79E-18	0.013	1.57E-17
19	Ruminococcus	2	1.000	0.014	0.000	3.22E-18	0.013	1.57E-17
20	Anaerotruncus	1	0.000	0.013	0.000	0.000	0.013	2.06E-17
21	Desulfitobacterium	1	0.000	0.013	0.000	2.73E-18	0.013	2.06E-17
22	Atopobium	2	0.000	0.014	0.003	0.000	0.019	1.57E-17
23	Eggerthella	1	0.000	0.014	0.000	1.65E-18	0.010	7.83E-18
24	Subdoligranulum	1	0.000	0.014	0.000	4.53E-18	0.010	7.83E-18
25	Bifidobacterium	10	0.378	0.025	1.000	0.412	0.026	0.412
26	Desulfatibacillum	7	0.714	0.025	0.043	0.362	0.018	0.362
27	Desulfomicrobium	5	1.000	0.025	0.000	0.289	0.013	0.289
28	Dyadobacter	8	0.429	0.025	0.222	0.325	0.021	0.325
29	Opitutus	7	0.714	0.025	0.043	0.362	0.018	0.362
30	Pelotomaculum	7	0.381	0.025	0.674	0.152	0.022	0.152
31	Phenylobacterium	7	0.524	0.025	0.289	0.306	0.019	0.306
32	Pseudoramibacter	6	0.467	0.025	0.537	0.146	0.019	0.146
33	Roseomonas	6	0.867	0.025	0.008	0.334	0.015	0.334
34	Selenomonas	5	0.600	0.025	0.126	0.139	0.015	0.139
35	Brachybacterium	8	0.321	0.025	0.262	0.103	0.025	0.103
36	Jonquetella	3	0.667	0.025	0.013	0.043	0.010	0.043
37	Methanocorpusculum	3	0.667	0.025	0.003	0.032	0.010	0.032
38	Pseudoflavonifractor	4	0.667	0.025	0.035	0.070	0.013	0.070
39	Pyramidobacter	2	1.000	0.025	0.000	0.024	0.008	0.024
40	Brachyspira	3	0.333	0.014	0.011	1.04E-17	0.019	6.18E-17
41	Cardiobacterium	2	1.000	0.014	0.000	1.71E-17	0.013	1.57E-17
42	Catenibacterium	3	0.333	0.014	0.017	1.92E-17	0.019	4.90E-17
43	Dorea	1	0.000	0.014	0.000	1.18E-17	0.007	1.42E-17
44	Bulleidia	1	0.000	0.014	0.000	3.95E-18	0.008	1.42E-17
45	Eubacterium	2	0.000	0.014	0.011	1.48E-17	0.014	4.12E-17
46	Capnocytophaga	1	0.000	0.025	0.000	0.004	0.006	0.004
47	Heliobacterium	3	0.000	0.025	0.383	0.025	0.015	0.025
48	Chlorobaculum	3	0.667	0.025	0.050	0.063	0.011	0.063
49	Finegoldia	2	1.000	0.024	0.000	0.021	0.008	0.021
50	Rhodothermus	3	0.667	0.025	0.050	0.063	0.011	0.063

51	Chromobacterium	1	0.000	0.013	0.000	3.00E-19	0.013	2.06E-17
52	Methanobrevibacter	1	0.000	0.013	0.000	0.000	0.013	2.06E-17
53	Fibrobacter	5	0.600	0.025	0.015	0.204	0.014	0.204
54	Desulfotomaculum	2	0.000	0.025	0.199	0.005	0.011	0.005
55	Oceanobacillus	3	0.333	0.025	0.289	0.031	0.013	0.031
56	Desulfovibrio	1	0.000	0.013	0.000	0.000	0.013	2.06E-17
57	Phascolarctobacterium	1	0.000	0.013	0.000	3.83E-19	0.013	2.06E-17
58	Magnetospirillum	3	1.000	0.025	0.000	0.103	0.009	0.103
59	Paenibacillus	3	1.000	0.025	0.000	0.103	0.009	0.103
60	Erysipelothrix	1	0.000	0.013	0.000	0.000	0.013	2.06E-17
61	Turicibacter	1	0.000	0.013	0.000	0.000	0.013	2.06E-17
62	Escherichia	1	0.000	0.013	0.000	4.63E-19	0.013	2.06E-17
63	Treponema	1	0.000	0.013	0.000	2.82E-18	0.013	2.06E-17
64	Faecalibacterium	1	0.000	0.013	0.000	0.000	0.013	2.06E-17
65	Leptotrichia	1	0.000	0.013	0.000	1.09E-18	0.013	2.06E-17
66	Gemella	1	0.000	0.014	0.000	8.35E-18	0.008	7.83E-18
67	Oxalobacter	3	0.000	0.014	0.020	1.57E-18	0.023	2.35E-17
68	Mitsuokella	2	0.000	0.025	0.199	0.004	0.012	0.004
69	Macrococcus	1	0.000	0.014	0.000	6.83E-18	0.008	7.83E-18
70	Prevotella	2	0.000	0.024	0.102	0.001	0.014	0.001
71	Moorella	1	0.000	0.025	0.000	0.006	0.005	0.006
72	Parabacteroides	1	0.000	0.013	0.000	0.000	0.013	2.06E-17
73	Roseburia	1	0.000	0.013	0.000	2.75E-18	0.013	2.06E-17
74	Slackia	1	0.000	0.024	0.000	0.000	0.008	0.000
75	Rubrobacter	1	0.000	0.013	0.000	0.000	0.013	2.06E-17
76	Scardovia	1	0.000	0.013	0.000	0.000	0.013	2.06E-17

Mutual exclusion network

Node_Index	Label	Degree	Cluster_Coeff	Closeness	Betweenness	Eigen_Vector	PageRank	HubScore
1	Abiotrophia	3	0.000	0.019	0.025	0.000	0.034	0.000
2	Anaerotruncus	1	0.000	0.019	0.000	0.000	0.012	0.000
3	Desulfitobacterium	1	0.000	0.019	0.000	0.000	0.012	0.000
4	Slackia	1	0.000	0.019	0.000	0.000	0.012	0.000
5	Actinomyces	1	0.000	0.018	0.000	4.03E-18	0.018	0.000
6	Desulfovibrio	1	0.000	0.018	0.000	5.57E-18	0.018	0.000
7	Alistipes	1	0.000	0.018	0.000	3.11E-18	0.018	0.000
8	Peptoniphilus	1	0.000	0.018	0.000	0.000	0.018	0.000
9	Anaerofustis	2	0.000	0.019	0.017	0.000	0.023	0.000
10	Rothia	2	0.000	0.019	0.017	0.000	0.023	0.000
11	Veillonella	1	0.000	0.019	0.000	0.000	0.012	0.000
12	Atopobium	1	0.000	0.019	0.000	0.000	0.012	0.000
13	Turicibacter	2	0.000	0.019	0.017	3.12E-18	0.023	0.000
14	Bifidobacterium	2	0.000	0.027	0.092	0.277	0.014	0.344
15	Parabacteroides	9	0.000	0.027	1.000	0.570	0.061	0.000
16	Roseburia	6	0.000	0.027	0.732	0.395	0.039	0.000
17	Bilophila	1	0.000	0.018	0.000	5.86E-18	0.018	0.000
18	Granulicatella	1	0.000	0.018	0.000	3.11E-18	0.018	0.000
19	Brachyspira	1	0.000	0.027	0.000	0.011	0.010	0.000
20	Fibrobacter	3	0.000	0.027	0.322	0.037	0.026	0.046
21	Caldicellulosiruptor	1	0.000	0.018	0.000	0.000	0.014	6.62E-18
22	Mobiluncus	2	0.000	0.018	0.008	7.85E-18	0.026	1.32E-17

23	Capnocytophaga	1	0.000	0.018	0.000	1.33E-18	0.018	0.000
24	Coprobacillus	1	0.000	0.018	0.000	8.33E-18	0.018	0.000
25	Cardiobacterium	1	0.000	0.027	0.000	0.011	0.010	0.000
26	Chromobacterium	1	0.000	0.018	0.000	0.000	0.014	6.62E-18
27	Clostridium	2	0.000	0.018	0.008	0.000	0.026	1.32E-17
28	Finegoldia	1	0.000	0.018	0.000	2.93E-18	0.014	6.62E-18
29	Coprococcus	1	0.000	0.019	0.000	0.000	0.012	0.000
30	Holdemania	3	0.000	0.019	0.025	0.000	0.034	0.000
31	Cryptobacterium	1	0.000	0.018	0.000	5.58E-19	0.014	6.62E-18
32	Sutterella	2	0.000	0.018	0.008	3.22E-18	0.026	1.32E-17
33	Desulfatibacillum	2	0.000	0.027	0.240	0.144	0.015	0.179
34	Dorea	3	0.000	0.027	0.474	0.108	0.022	0.000
35	Desulfomicrobium	2	0.000	0.027	0.092	0.277	0.014	0.344
36	Dialister	1	0.000	0.019	0.000	0.000	0.012	0.000
37	Opitutus	2	0.000	0.027	0.309	0.194	0.015	0.242
38	Eggerthella	2	0.000	0.019	0.017	0.000	0.023	0.000
39	Erysipelothrix	1	0.000	0.019	0.000	0.000	0.012	0.000
40	Enterococcus	1	0.000	0.027	0.000	0.056	0.010	0.000
41	Phenylobacterium	3	0.000	0.027	0.322	0.196	0.025	0.243
42	Heliobacterium	1	0.000	0.027	0.000	0.163	0.008	0.203
43	Solobacterium	1	0.000	0.019	0.000	0.000	0.012	0.000
44	Jonquetella	1	0.000	0.027	0.000	0.163	0.008	0.203
45	Lactococcus	1	0.000	0.027	0.000	0.056	0.010	0.000
46	Porphyromonas	1	0.000	0.018	0.000	0.000	0.014	6.62E-18
47	Oceanobacillus	1	0.000	0.027	0.000	0.012	0.010	0.015
48	Streptococcus	3	0.000	0.027	0.322	0.043	0.027	0.000
49	Pseudoramibacter	2	0.000	0.027	0.092	0.277	0.014	0.344
50	Roseomonas	1	0.000	0.027	0.000	0.163	0.008	0.203
51	Selenomonas	2	0.000	0.027	0.092	0.277	0.014	0.344
52	Pelotomaculum	2	0.000	0.027	0.446	0.126	0.016	0.156
53	Peptostreptococcus	1	0.000	0.018	0.000	5.95E-18	0.018	0.000
54	Shuttleworthia	1	0.000	0.018	0.000	5.98E-18	0.018	0.000
55	Succinatimonas	1	0.000	0.019	0.000	0.000	0.012	0.000
56	Ruminococcus	1	0.000	0.018	0.000	0.000	0.014	6.62E-18
57	Thermanaerovibrio	1	0.000	0.027	0.000	0.012	0.010	0.015

Co-occurrence network

Node_Index	Label	Degree	Cluster_Coeff	Closeness	Betweenness	Eigen_Vector	PageRank	HubScore
1	Abiotrophia	5	0.500	0.117	0.005	0.074	0.010	0.074
2	Anaerostipes	5	0.700	0.123	0.012	0.093	0.010	0.093
3	Anaerotruncus	9	0.667	0.127	0.044	0.171	0.015	0.171
4	Butyrivibrio	13	0.590	0.125	0.040	0.220	0.021	0.220
5	Eubacterium	9	0.583	0.120	0.023	0.132	0.016	0.132
6	Oribacterium	10	0.667	0.127	0.048	0.199	0.017	0.199
7	Acetivibrio	4	1.000	0.118	0.000	0.004	0.011	0.004
8	Anaerofustis	7	0.619	0.120	0.033	0.006	0.018	0.006
9	Brachyspira	9	0.528	0.129	0.822	0.025	0.021	0.025
10	Finegoldia	9	0.556	0.120	0.086	0.007	0.022	0.007
11	Streptobacillus	8	0.679	0.119	0.012	0.006	0.019	0.006
12	Aliivibrio	8	0.607	0.119	0.083	0.005	0.020	0.005
13	Bulleidia	3	1.000	0.108	0.000	0.001	0.009	0.001
14	Photobacterium	8	0.536	0.119	0.128	0.005	0.021	0.005
15	Succinatimonas	3	1.000	0.108	0.000	0.001	0.009	0.001
16	Tolumonas	8	0.536	0.119	0.128	0.005	0.021	0.005
17	Alistipes	6	0.800	0.129	0.093	0.097	0.012	0.097
18	Dysgonomonas	13	0.538	0.137	0.511	0.190	0.023	0.190
19	Fibrobacter	13	0.590	0.136	0.425	0.203	0.023	0.203
20	Heliobacterium	11	0.655	0.131	0.126	0.169	0.021	0.169
21	Oxalobacter	4	0.500	0.126	0.740	0.045	0.011	0.045
22	Slackia	14	0.516	0.138	1.000	0.224	0.024	0.224
23	Solobacterium	14	0.473	0.135	0.311	0.237	0.024	0.237
24	Bifidobacterium	4	0.167	0.124	0.472	0.010	0.013	0.010
25	Peptostreptococcus	5	0.600	0.122	0.047	0.012	0.012	0.012
26	Faecalibacterium	10	0.733	0.125	0.009	0.199	0.017	0.199
27	Pseudoflavonifractor	18	0.392	0.135	0.425	0.304	0.029	0.304
28	Subdoligranulum	15	0.495	0.134	0.305	0.284	0.024	0.284
29	Holdemania	15	0.514	0.132	0.266	0.274	0.024	0.274
30	Atopobium	1	0.000	0.094	0.000	8.01E-06	0.007	8.01E-06
31	Coprobacillus	3	0.333	0.102	0.118	8.84E-05	0.018	8.84E-05
32	Bacteroides	1	0.000	0.016	0.000	2.03E-18	0.016	0.000
33	Parabacteroides	1	0.000	0.016	0.000	0.000	0.016	0.000
34	Bilophila	7	0.476	0.131	0.555	0.091	0.015	0.091
35	Peptoniphilus	3	0.333	0.112	0.342	0.001	0.015	0.001
36	Eggerthella	8	0.857	0.130	0.013	0.130	0.015	0.130
37	Blautia	13	0.615	0.126	0.042	0.235	0.021	0.235
38	Capnocytophaga	9	0.417	0.129	0.170	0.126	0.017	0.126
39	Coprococcus	11	0.582	0.125	0.134	0.175	0.020	0.175
40	Dorea	8	0.821	0.121	0.005	0.140	0.014	0.140
41	Roseburia	11	0.709	0.125	0.015	0.199	0.018	0.199
42	Ruminococcus	12	0.606	0.126	0.041	0.216	0.020	0.216
43	Selenomonas	9	0.361	0.125	0.143	0.129	0.018	0.129
44	Campylobacter	1	0.000	0.016	0.000	1.13E-18	0.012	2.79E-18
45	Helicobacter	2	0.000	0.016	0.002	3.53E-18	0.023	5.59E-18
46	Mitsuokella	4	0.667	0.122	0.002	0.042	0.009	0.042
47	Porphyromonas	5	0.500	0.124	0.012	0.066	0.010	0.066
48	Citrobacter	2	1.000	0.090	0.000	7.95E-06	0.010	7.95E-06
49	Escherichia	5	0.500	0.098	0.115	4.38E-05	0.022	4.38E-05
50	Shigella	5	0.500	0.098	0.115	4.38E-05	0.022	4.38E-05

51	Clostridium	5	0.700	0.125	0.020	0.094	0.010	0.094
52	Collinsella	1	0.000	0.112	0.000	0.012	0.004	0.012
53	Fusobacterium	2	1.000	0.102	0.000	8.77E-05	0.012	8.77E-05
54	Shuttleworthia	1	0.000	0.112	0.000	0.016	0.004	0.016
55	Dialister	9	0.639	0.130	0.119	0.143	0.016	0.143
56	Pseudoramibacter	8	0.929	0.130	0.002	0.140	0.014	0.140
57	Pyramidobacter	10	0.800	0.132	0.011	0.183	0.017	0.183
58	Enterobacter	2	0.000	0.115	0.646	0.004	0.008	0.004
59	Klebsiella	4	0.500	0.106	0.549	0.000	0.017	0.000
60	Streptococcus	2	1.000	0.090	0.000	7.95E-06	0.010	7.95E-06
61	Veillonella	3	1.000	0.098	0.000	4.25E-05	0.013	4.25E-05
62	Haemophilus	2	1.000	0.108	0.000	0.001	0.007	0.001
63	Lactobacillus	1	0.000	0.016	0.000	0.000	0.012	2.79E-18
64	Megasphaera	1	0.000	0.117	0.000	0.015	0.004	0.015

Mutual exclusion network

Node_Index	Label	Degree	Cluster_Coeff	Closeness	Betweenness	Eigen_Vector	PageRank	HubScore
1	Anaerotruncus	1	0.000	0.200	0.000	1.30E-16	0.167	5.55E-17
2	Streptococcus	1	0.000	0.200	0.000	1.76E-16	0.167	5.55E-17
3	Bifidobacterium	1	0.000	0.294	0.000	0.408	0.116	0.500
4	Paraprevotella	3	0.000	0.333	1.000	0.707	0.320	0.000
5	Eggerthella	1	0.000	0.294	0.000	0.408	0.116	0.500
6	Peptoniphilus	1	0.000	0.294	0.000	0.408	0.116	0.500

Co-occurrence network

Node_Index	Label	Degree	Cluster_Coeff	Closeness	Betweenness	Eigen_Vector	PageRank	HubScore
1	Acidovorax	3	1	0.019	0.000	0.000	0.018	1.58E-17
2	Geobacter	3	1	0.019	0.000	0.000	0.018	1.58E-17
3	Thermomonospora	3	1	0.019	0.000	0.000	0.018	1.58E-17
4	Thioalkalivibrio	3	1	0.019	0.000	0.000	0.018	1.58E-17
5	Actinosynnema	7	1	0.021	0.000	0.354	0.018	0.354
6	Dermacoccus	7	1	0.021	0.000	0.354	0.018	0.354
7	Edwardsiella	7	1	0.021	0.000	0.354	0.018	0.354
8	Haliangium	7	1	0.021	0.000	0.354	0.018	0.354
9	Jonquetella	7	1	0.021	0.000	0.354	0.018	0.354
10	Myxococcus	7	1	0.021	0.000	0.354	0.018	0.354
11	Robiginitalea	7	1	0.021	0.000	0.354	0.018	0.354
12	Streptosporangium	7	1	0.021	0.000	0.354	0.018	0.354
13	Alkalilimnicola	2	1	0.019	0.000	0.000	0.018	1.81E-17
14	Kytococcus	2	1	0.019	0.000	0.000	0.018	1.81E-17
15	Rhodospirillum	2	1	0.019	0.000	0.000	0.018	1.81E-17
16	Anaeromyxobacter	2	1	0.019	0.000	0.000	0.018	1.81E-17
17	Desmospora	2	1	0.019	0.000	0.000	0.018	1.81E-17
18	Kineococcus	2	1	0.019	0.000	0.000	0.018	1.81E-17
19	Azoarcus	1	0	0.019	0.000	0.000	0.018	2.96E-18
20	Pseudoflavonifractor	1	0	0.019	0.000	4.32E-18	0.018	2.96E-18
21	Beutenbergia	1	0	0.019	0.000	0.000	0.018	2.96E-18
22	Desulfitobacterium	1	0	0.019	0.000	0.000	0.018	2.96E-18
23	Blattabacterium	4	1	0.020	0.000	0.000	0.018	3.63E-17
24	Citrobacter	4	1	0.020	0.000	0.000	0.018	3.63E-17
25	Enterobacter	4	1	0.020	0.000	0.000	0.018	3.63E-17
26	Methanocorpusculum	4	1	0.020	0.000	0.000	0.018	3.63E-17
27	Streptobacillus	4	1	0.020	0.000	0.000	0.018	3.63E-17
28	Caldanaerobacter	3	1	0.019	0.000	0.000	0.018	1.58E-17
29	Nocardioides	3	1	0.019	0.000	0.000	0.018	1.58E-17
30	Phenylobacterium	3	1	0.019	0.000	0.000	0.018	1.58E-17
31	Syntrophomonas	3	1	0.019	0.000	0.000	0.018	1.58E-17
32	Carboxydothermus	2	1	0.019	0.000	0.000	0.018	1.81E-17
33	Methylococcus	2	1	0.019	0.000	0.000	0.018	1.81E-17
34	Opitutus	2	1	0.019	0.000	0.000	0.018	1.81E-17
35	Chlorobaculum	1	0	0.019	0.000	0.000	0.018	2.96E-18
36	Holdemania	1	0	0.019	0.000	0.000	0.018	2.96E-18
37	Cronobacter	2	1	0.019	0.000	0.000	0.018	1.81E-17
38	Klebsiella	2	1	0.019	0.000	0.000	0.018	1.81E-17
39	Salmonella	2	1	0.019	0.000	0.000	0.018	1.81E-17
40	Deinococcus	1	0	0.019	0.000	0.000	0.018	2.96E-18
41	Erythrobacter	1	0	0.019	0.000	0.000	0.018	2.96E-18
42	Heliobacterium	3	1	0.019	0.000	0.000	0.018	1.58E-17
43	Pelotomaculum	3	1	0.019	0.000	0.000	0.018	1.58E-17
44	Pseudoramibacter	3	1	0.019	0.000	0.000	0.018	1.58E-17
45	Thermanaerovibrio	3	1	0.019	0.000	0.000	0.018	1.58E-17
46	Pelobacter	1	0	0.019	0.000	0.000	0.018	2.96E-18
47	Treponema	1	0	0.019	0.000	0.000	0.018	2.96E-18
48	Peptoniphilus	1	0	0.019	0.000	0.000	0.018	2.96E-18
49	Solobacterium	1	0	0.019	0.000	1.40E-18	0.018	2.96E-18
50	Rhodothermus	3	1	0.019	0.000	0.000	0.018	1.58E-17
51	Rubrobacter	3	1	0.019	0.000	0.000	0.018	1.58E-17

52	Sphaerobacter	3	1	0.019	0.000	0.000	0.018	1.58E-17
53	Symbiobacterium	3	1	0.019	0.000	0.000	0.018	1.58E-17
54	Rothia	1	0	0.019	0.000	0.000	0.018	2.96E-18
55	Streptococcus	1	0	0.019	0.000	0.000	0.018	2.96E-18

Mutual exclusion network

Node_Index	Label	Degree	Cluster_Coeff	Closeness	Betweenness	Eigen_Vector	PageRank	HubScore
1	Akkermansia	1	0	0.043	0.000	0.000	0.042	1.63E-17
2	Haemophilus	1	0	0.043	0.000	0.000	0.042	1.63E-17
3	Alistipes	2	0	0.045	0.167	0.000	0.061	1.26E-16
4	Rothia	1	0	0.045	0.000	0.000	0.032	6.32E-17
5	Streptococcus	1	0	0.045	0.000	0.000	0.032	6.32E-17
6	Caldanaerobacter	1	0	0.050	0.000	0.354	0.027	0.224
7	Faecalibacterium	4	0	0.050	1.000	0.707	0.099	0.894
8	Collinsella	1	0	0.043	0.000	0.000	0.042	1.63E-17
9	Eggerthella	1	0	0.043	0.000	0.000	0.042	1.63E-17
10	Desulfovibrio	1	0	0.043	0.000	0.000	0.042	1.63E-17
11	Thiobacillus	1	0	0.043	0.000	0.000	0.042	1.63E-17
12	Dorea	1	0	0.043	0.000	0.000	0.042	1.63E-17
13	Shigella	1	0	0.043	0.000	0.000	0.042	1.63E-17
14	Nocardioides	1	0	0.050	0.000	0.354	0.027	0.224
15	Phenylobacterium	1	0	0.050	0.000	0.354	0.027	0.224
16	Syntrophomonas	1	0	0.050	0.000	0.354	0.027	0.224
17	Fusobacterium	1	0	0.043	0.000	0.000	0.042	1.63E-17
18	Turicibacter	1	0	0.043	0.000	0.000	0.042	1.63E-17
19	Lautropia	1	0	0.043	0.000	0.000	0.042	1.63E-17
20	Mitsuokella	1	0	0.043	0.000	0.000	0.042	1.63E-17
21	Leptothrix	1	0	0.043	0.000	0.000	0.042	1.63E-17
22	Thermoanaerobacter	1	0	0.043	0.000	0.000	0.042	1.63E-17
23	Pyramidobacter	1	0	0.043	0.000	0.000	0.042	1.63E-17
24	Subdoligranulum	1	0	0.043	0.000	0.000	0.042	1.63E-17

Co-occurrence network

Node_Index	Label	Degree	Cluster_Coeff	Closeness	Betweenness	Eigen_Vector	PageRank	HubScore
1	Abiotrophia	4	0.333	0.069	0.436	0.016	0.009	0.0163
2	Anaerofustis	3	0.333	0.065	0.150	0.001	0.011	0.0009
3	Finegoldia	2	1.000	0.065	0.000	0.001	0.007	0.0009
4	Holdemania	24	0.475	0.072	0.538	0.181	0.020	0.1813
5	Roseburia	20	0.437	0.072	0.374	0.134	0.018	0.1338
6	Acetivibrio	2	0.000	0.068	0.002	0.011	0.003	0.0115
7	Alistipes	22	0.442	0.071	0.321	0.155	0.019	0.1550
8	Blautia	13	0.410	0.070	0.165	0.068	0.013	0.0676
9	Achromobacter	2	1.000	0.059	0.000	1.91E-06	0.008	1.91E-06
10	Cronobacter	4	0.333	0.062	0.295	3.53E-05	0.014	3.53E-05
11	Serratia	2	1.000	0.059	0.000	1.91E-06	0.008	1.91E-06
12	Acidaminococcus	3	0.667	0.065	0.208	0.000	0.010	0.0003
13	Megasphaera	4	0.333	0.069	0.957	0.006	0.009	0.0062
14	Mitsuokella	5	0.300	0.066	0.503	0.000	0.016	0.0003
15	Actinomyces	1	0.000	0.012	0.000	1.58E-18	0.012	0.0000
16	Rothia	1	0.000	0.012	0.000	4.31E-18	0.012	0.0000
17	Akkermansia	3	0.667	0.068	0.002	0.020	0.004	0.0202
18	Bilophila	11	0.327	0.070	0.112	0.054	0.011	0.0542
19	Haemophilus	22	0.563	0.071	0.107	0.184	0.017	0.1837
20	Alicyclobacillus	25	0.517	0.072	0.195	0.207	0.019	0.2073
21	Alkaliphilus	18	0.490	0.071	0.140	0.128	0.015	0.1280
22	Anaerotruncus	23	0.553	0.072	0.121	0.187	0.018	0.1869
23	Collinsella	13	0.744	0.071	0.021	0.126	0.011	0.1261
24	Cryptobacterium	29	0.510	0.072	0.164	0.240	0.022	0.2403
25	Desulfomicrobium	26	0.486	0.072	0.288	0.198	0.020	0.1982
26	Desulfotomaculum	17	0.566	0.071	0.046	0.132	0.014	0.1320
27	Faecalibacterium	16	0.383	0.072	1.000	0.090	0.015	0.0901
28	Fibrobacter	28	0.550	0.072	0.113	0.240	0.021	0.2396
29	Heliobacterium	25	0.540	0.072	0.083	0.213	0.019	0.2126
30	Lautropia	14	0.736	0.070	0.018	0.121	0.011	0.1209
31	Moorella	24	0.562	0.072	0.108	0.205	0.018	0.2053
32	Paraprevotella	10	0.422	0.070	0.051	0.049	0.010	0.0494
33	Phascolarctobacterium	19	0.532	0.071	0.094	0.150	0.015	0.1496
34	Pseudoflavonifractor	23	0.589	0.071	0.070	0.196	0.017	0.1961
35	Pyramidobacter	28	0.550	0.072	0.105	0.242	0.021	0.2416
36	Slackia	14	0.879	0.070	0.003	0.135	0.011	0.1351
37	Subdoligranulum	15	0.590	0.071	0.087	0.133	0.012	0.1325
38	Symbiobacterium	22	0.662	0.071	0.033	0.202	0.016	0.2024
39	Syntrophomonas	22	0.576	0.071	0.196	0.186	0.018	0.1858
40	Thioalkalivibrio	21	0.629	0.071	0.055	0.184	0.016	0.1838
41	Treponema	17	0.515	0.072	0.211	0.118	0.015	0.1179
42	Bulleidia	21	0.548	0.071	0.096	0.170	0.016	0.1697
43	Desulfitobacterium	14	0.648	0.071	0.050	0.115	0.012	0.1151
44	Dysgonomonas	18	0.510	0.071	0.102	0.127	0.015	0.1267
45	Oxalobacter	3	0.333	0.068	0.150	0.018	0.006	0.0176
46	Paenibacillus	12	0.470	0.071	0.086	0.086	0.011	0.0858
47	Porphyromonas	9	0.500	0.070	0.029	0.051	0.009	0.0512
48	Pseudoramibacter	11	0.673	0.070	0.015	0.087	0.010	0.0865
49	Selenomonas	21	0.529	0.072	0.164	0.170	0.017	0.1702
50	Shuttleworthia	18	0.654	0.071	0.036	0.156	0.014	0.1559
51	Sutterella	8	0.393	0.071	0.084	0.039	0.008	0.0390

52	Thermanaerovibrio	20	0.653	0.071	0.047	0.180	0.015	0.1800
53	Butyrivibrio	10	0.400	0.070	0.119	0.039	0.011	0.0385
54	Eubacterium	7	0.429	0.069	0.166	0.021	0.009	0.0214
55	Oribacterium	17	0.493	0.072	0.112	0.116	0.014	0.1164
56	Solobacterium	10	0.533	0.070	0.302	0.066	0.012	0.0662
57	Succinatimonas	9	0.500	0.070	0.035	0.054	0.009	0.0540
58	Anaerococcus	1	0.000	0.012	0.000	4.81E-18	0.008	0.0000
59	Staphylococcus	2	0.000	0.012	0.004	8.15E-18	0.015	0.0000
60	Variovorax	1	0.000	0.062	0.000	4.57E-05	0.005	4.57E-05
61	Anaerostipes	2	1.000	0.012	0.000	6.05E-18	0.012	0.0000
62	Clostridium	3	0.333	0.012	0.004	1.86E-18	0.017	0.0000
63	Eggerthella	2	1.000	0.012	0.000	7.56E-18	0.012	0.0000
64	Parabacteroides	9	0.333	0.070	0.036	0.040	0.009	0.0404
65	Bacteroides	6	0.467	0.069	0.019	0.013	0.007	0.0134
66	Peptostreptococcus	10	0.289	0.070	0.101	0.045	0.010	0.0446
67	Thiobacillus	9	0.389	0.070	0.056	0.031	0.009	0.0312
68	Bifidobacterium	2	0.000	0.012	0.004	1.01E-17	0.015	0.0000
69	Peptoniphilus	1	0.000	0.012	0.000	7.59E-19	0.008	0.0000
70	Prevotella	13	0.603	0.070	0.028	0.093	0.011	0.0928
71	Coprobacillus	2	1.000	0.067	0.000	0.005	0.004	0.0055
72	Dialister	4	0.833	0.069	0.000	0.026	0.005	0.0256
73	Capnocytophaga	13	0.487	0.071	0.079	0.089	0.011	0.0886
74	Desulfovibrio	6	0.600	0.070	0.148	0.030	0.007	0.0300
75	Catenibacterium	2	1.000	0.067	0.000	0.004	0.005	0.0036
76	Gemella	2	1.000	0.067	0.000	0.004	0.005	0.0036
77	Chromobacterium	1	0.000	0.064	0.000	0.001	0.004	0.0009
78	Citrobacter	2	1.000	0.062	0.000	1.85E-05	0.008	1.85E-05
79	Enterobacter	2	1.000	0.062	0.000	1.85E-05	0.008	1.85E-05
80	Ruminococcus	1	0.000	0.012	0.000	6.88E-18	0.007	0.0000
81	Coprococcus	6	1.000	0.070	0.000	0.040	0.006	0.0401
82	Dorea	1	0.000	0.066	0.000	0.001	0.003	0.0011
83	Pelotomaculum	1	0.000	0.012	0.000	3.16E-18	0.012	0.0000
84	Scardovia	1	0.000	0.012	0.000	0.000	0.012	0.0000

Mutual exclusion network

Node_Index	Label	Degree	Cluster_Coeff	Closeness	Betweenness	Eigen_Vector	PageRank	HubScore
1	Acetivibrio	1	0.000	0.130	0.000	0.159	6.29E+14	0.2246
2	Mobiluncus	7	0.000	0.144	1.000	0.480	-3.53E+15	0.0072
3	Alistipes	1	0.000	0.118	0.000	0.101	-5.55E+14	0.1428
4	Stenotrophomonas	6	0.000	0.130	0.528	0.305	2.67E+15	0.0046
5	Anaerotruncus	1	0.000	0.130	0.000	0.159	6.29E+14	0.2246
6	Bacteroides	2	0.000	0.141	0.719	0.298	4.95E+14	0.4208
7	Enterococcus	8	0.000	0.137	0.839	0.419	8.61E+14	0.0063
8	Capnocytophaga	1	0.000	0.124	0.000	0.139	-1.34E+14	0.1962
9	Clostridium	2	0.000	0.042	0.007	1.70E-17	-0.418	0.0000
10	Pelotomaculum	1	0.000	0.042	0.000	5.20E-18	-0.070	0.0000
11	Scardovia	1	0.000	0.042	0.000	1.39E-17	0.050	0.0000
12	Dysgonomonas	2	0.000	0.137	0.262	0.260	7.40E+13	0.3675
13	Heliobacterium	1	0.000	0.124	0.000	0.139	-1.34E+14	0.1962
14	Paenibacillus	1	0.000	0.124	0.000	0.139	-1.34E+14	0.1962
15	Parabacteroides	1	0.000	0.124	0.000	0.139	-1.34E+14	0.1962

16	Paraprevotella	1	0.000	0.124	0.000	0.139	-1.34E+14	0.1962
17	Prevotella	1	0.000	0.124	0.000	0.139	-1.34E+14	0.1962
18	Thiobacillus	1	0.000	0.124	0.000	0.139	-1.34E+14	0.1962
19	Haemophilus	1	0.000	0.130	0.000	0.159	6.29E+14	0.2246
20	Porphyromonas	1	0.000	0.130	0.000	0.159	6.29E+14	0.2246
21	Sutterella	2	0.000	0.137	0.262	0.260	7.40E+13	0.3675
22	Pseudoramibacter	1	0.000	0.118	0.000	0.101	-5.55E+14	0.1428
23	Selenomonas	1	0.000	0.118	0.000	0.101	-5.55E+14	0.1428
24	Shigella	1	0.000	0.040	0.000	1.95E-18	-0.053	0.0000
25	Variovorax	1	0.000	0.040	0.000	0.000	-0.168	0.0000
26	Shuttleworthia	1	0.000	0.118	0.000	0.101	-5.55E+14	0.1428

Co-occurrence network

Node_Index	Label	Degree	Cluster_Coeff	Closeness	Betweenness	Eigen_Vector	PageRank	HubScore
1	Abiotrophia	3	0.333	0.065	0.214	0.006	0.011	0.006
2	Butyrivibrio	5	0.400	0.067	0.444	0.017	0.014	0.017
3	Eubacterium	2	0.000	0.064	0.171	0.002	0.009	0.002
4	Streptobacillus	8	0.500	0.067	0.320	0.026	0.020	0.026
5	Acetivibrio	4	0.333	0.069	0.796	0.042	0.010	0.042
6	Desulfitobacterium	6	0.467	0.069	0.370	0.151	0.015	0.151
7	Finegoldia	9	0.556	0.068	1.000	0.038	0.021	0.038
8	Leptotrichia	8	0.714	0.067	0.361	0.033	0.018	0.033
9	Moorella	7	0.381	0.069	0.498	0.117	0.018	0.117
10	Acholeplasma	3	1.000	0.064	0.000	0.010	0.008	0.010
11	Anaerococcus	9	0.639	0.066	0.116	0.032	0.021	0.032
12	Mesoplasma	4	0.833	0.064	0.002	0.014	0.010	0.014
13	Thermoanaerobacter	12	0.439	0.067	0.464	0.036	0.028	0.036
14	Aerococcus	1	0.000	0.063	0.000	0.004	0.004	0.004
15	Akkermansia	2	1.000	0.063	0.000	0.013	0.008	0.013
16	Magnetospirillum	5	0.500	0.067	0.161	0.062	0.015	0.062
17	Rhodospirillum	4	0.667	0.066	0.046	0.042	0.013	0.042
18	Alicyclobacillus	10	0.422	0.069	0.265	0.268	0.024	0.268
19	Heliobacterium	11	0.509	0.069	0.554	0.329	0.026	0.329
20	Holdemania	4	0.833	0.068	0.004	0.149	0.010	0.149
21	Pelotomaculum	9	0.250	0.070	0.699	0.176	0.024	0.176
22	Pseudoflavonifractor	4	0.667	0.067	0.008	0.103	0.011	0.103
23	Pseudoramibacter	11	0.509	0.069	0.161	0.330	0.026	0.330
24	Pyramidobacter	13	0.397	0.070	0.813	0.362	0.030	0.362
25	Roseomonas	10	0.311	0.069	0.912	0.160	0.029	0.160
26	Symbiobacterium	9	0.583	0.069	0.144	0.298	0.021	0.298
27	Alistipes	2	0.000	0.066	0.376	0.020	0.010	0.020
28	Parabacteroides	2	0.000	0.062	0.192	0.003	0.013	0.003
29	Alkaliphilus	6	0.800	0.065	0.012	0.023	0.014	0.023
30	Caldicellulosiruptor	9	0.694	0.067	0.077	0.033	0.020	0.033
31	Fusobacterium	9	0.611	0.066	0.204	0.031	0.021	0.031
32	Gemella	5	0.500	0.064	0.036	0.015	0.013	0.015
33	Turicibacter	10	0.622	0.066	0.105	0.034	0.022	0.034
34	Anaerofustis	4	0.333	0.069	0.243	0.101	0.011	0.101
35	Eggerthella	9	0.528	0.069	0.468	0.270	0.024	0.270
36	Peptostreptococcus	3	0.333	0.069	0.656	0.039	0.009	0.039
37	Anaerostipes	2	1.000	0.015	0.000	0.000	0.015	7.97E-18
38	Blautia	2	1.000	0.015	0.000	0.000	0.015	7.97E-18
39	Clostridium	2	1.000	0.015	0.000	0.000	0.015	7.97E-18
40	Anaerotruncus	1	0.000	0.062	0.000	0.004	0.006	0.004
41	Subdoligranulum	2	0.000	0.065	0.192	0.034	0.010	0.034
42	Atopobium	6	0.533	0.067	0.224	0.165	0.018	0.165
43	Collinsella	5	0.300	0.067	0.559	0.100	0.017	0.100
44	Mobiluncus	1	0.000	0.063	0.000	0.020	0.005	0.020
45	Slackia	10	0.533	0.069	0.305	0.292	0.025	0.292
46	Bacteroides	1	0.000	0.059	0.000	0.000	0.008	0.000
47	Bifidobacterium	1	0.000	0.063	0.000	0.012	0.005	0.012
48	Bilophila	1	0.000	0.065	0.000	0.020	0.005	0.020
49	Bulleidia	1	0.000	0.015	0.000	0.000	0.015	3.98E-18
50	Granulicatella	1	0.000	0.015	0.000	0.000	0.015	3.98E-18
51	Oribacterium	2	0.000	0.068	0.437	0.037	0.007	0.037

52	Capnocytophaga	2	1.000	0.016	0.000	0.000	0.014	1.65E-18
53	Paraprevotella	2	1.000	0.016	0.000	0.000	0.014	1.65E-18
54	Prevotella	3	0.333	0.016	0.014	0.000	0.021	2.47E-18
55	Coprococcus	3	0.000	0.065	0.363	0.013	0.014	0.013
56	Dialister	1	0.000	0.062	0.000	0.002	0.006	0.002
57	Dysgonomonas	1	0.000	0.016	0.000	0.000	0.009	0.000
58	Porphyromonas	2	0.000	0.016	0.011	0.000	0.016	1.65E-18
59	Selenomonas	10	0.467	0.069	0.579	0.283	0.025	0.283
60	Faecalibacterium	1	0.000	0.015	0.000	0.000	0.015	3.98E-18
61	Roseburia	1	0.000	0.015	0.000	0.000	0.015	3.98E-18
62	Peptoniphilus	2	1.000	0.063	0.000	0.006	0.006	0.006
63	Jonquetella	7	0.429	0.068	0.198	0.110	0.019	0.110
64	Oxalobacter	6	0.533	0.068	0.114	0.118	0.016	0.118
65	Treponema	2	1.000	0.065	0.000	0.028	0.007	0.028
66	Mitsuokella	2	1.000	0.066	0.000	0.071	0.006	0.071
67	Thermanaerovibrio	1	0.000	0.066	0.000	0.022	0.005	0.022
68	Shuttleworthia	4	1.000	0.068	0.000	0.156	0.010	0.156

Mutual exclusion network

Node_Index	Label	Degree	Cluster_Coeff	Closeness	Betweenness	Eigen_Vector	PageRank	HubScore
1	Anaerococcus	2	0.000	0.067	0.128	0.271	0.030	0.357
2	Bacteroides	9	0.000	0.069	1.000	0.659	0.132	0.000
3	Parabacteroides	3	0.000	0.066	0.151	0.192	0.046	0.000
4	Anaerofustis	1	0.000	0.065	0.000	0.055	0.019	0.072
5	Blautia	6	0.000	0.068	0.639	0.171	0.097	0.000
6	Anaerostipes	3	0.000	0.040	0.100	0.000	0.053	0.000
7	Capnocytophaga	2	0.000	0.040	0.046	0.000	0.037	7.24E-18
8	Paraprevotella	1	0.000	0.040	0.000	0.000	0.020	0.000
9	Prevotella	2	0.000	0.040	0.073	0.000	0.036	0.000
10	Caldicellulosiruptor	1	0.000	0.067	0.000	0.210	0.017	0.276
11	Coprococcus	1	0.000	0.067	0.000	0.210	0.017	0.276
12	Eubacterium	1	0.000	0.067	0.000	0.210	0.017	0.276
13	Finegoldia	2	0.000	0.067	0.128	0.271	0.030	0.357
14	Mitsuokella	1	0.000	0.067	0.000	0.210	0.017	0.276
15	Selenomonas	2	0.000	0.068	0.603	0.264	0.031	0.348
16	Streptobacillus	1	0.000	0.067	0.000	0.210	0.017	0.276
17	Thermoanaerobacter	1	0.000	0.067	0.000	0.210	0.017	0.276
18	Eggerthella	1	0.000	0.065	0.000	0.055	0.019	0.072
19	Pyramidobacter	1	0.000	0.065	0.000	0.055	0.019	0.072
20	Shuttleworthia	1	0.000	0.065	0.000	0.055	0.019	0.072
21	Slackia	1	0.000	0.065	0.000	0.055	0.019	0.072
22	Bulleidia	1	0.000	0.034	0.000	0.000	0.025	0.000
23	Dysgonomonas	2	0.000	0.034	0.009	0.000	0.047	0.000
24	Clostridium	1	0.000	0.039	0.000	0.000	0.021	0.000
25	Dialister	2	0.000	0.034	0.009	0.000	0.047	0.000
26	Lawsonia	1	0.000	0.034	0.000	0.000	0.025	0.000
27	Phascolarctobacterium	1	0.000	0.034	0.000	0.000	0.025	0.000
28	Granulicatella	1	0.000	0.034	0.000	0.000	0.025	0.000
29	Solobacterium	1	0.000	0.063	0.000	0.061	0.018	0.081
30	Porphyromonas	1	0.000	0.039	0.000	0.000	0.021	0.000
31	Ruminococcus	2	0.000	0.040	0.046	0.000	0.038	0.000
