# Supplementary material for: Global investigation of composition and interaction networks in gut microbiomes of individuals belonging to diverse geographies and age-groups
Source: Gut Pathog. 2016 May 6;8:17. doi: 10.1186/s13099-016-0099-z (PMC4858888; doi:10.1186/s13099-016-0099-z)
Supplement: Supplementary file 11 — 10.1186/s13099-016-0099-z Genera occurrences in the common networks between specific pairs of nationalities. [file 13099_2016_99_MOESM11_ESM.pdf]

Additional File 11: Centrality measures of different genera in the gut microbiomes of common nodes between combination of geographies for co-occurrence and mutual exclusion networks

Co-occurrence network

Node_Index	Label	Degree	Cluster_Coeff	Closeness	Betweenness	Eigen_Vector	PageRank	HubScore
1	Abiotrophia	12	0.424	0.116	0.674	0.130	0.028	0.130
2	Anaerococcus	8	0.714	0.113	0.049	0.079	0.019	0.079
3	Anaerofustis	10	0.422	0.114	0.238	0.118	0.022	0.118
4	Anaerostipes	11	0.545	0.115	0.426	0.135	0.025	0.135
5	Anaerotruncus	2	1.000	0.105	0.000	0.028	0.007	0.028
6	Atopobium	16	0.417	0.115	0.671	0.205	0.033	0.205
7	Bacteroides	1	0.000	0.022	0.000	1.82E-18	0.022	4.00E-18
8	Bilophila	1	0.000	0.105	0.000	0.017	0.005	0.017
9	Blautia	3	1.000	0.105	0.000	0.020	0.010	0.020
10	Bulleidia	3	1.000	0.111	0.000	0.047	0.009	0.047
11	Butyrivibrio	6	0.467	0.109	0.547	0.049	0.020	0.049
12	Capnocytophaga	2	1.000	0.024	0.000	0.000	0.018	7.99E-18
13	Catenibacterium	15	0.438	0.117	1.000	0.183	0.035	0.183
14	Clostridium	6	0.733	0.111	0.082	0.054	0.016	0.054
15	Collinsella	17	0.610	0.117	0.405	0.275	0.034	0.275
16	Coprobacillus	1	0.000	0.106	0.000	0.014	0.005	0.014
17	Coprococcus	15	0.505	0.117	0.512	0.199	0.032	0.199
18	Dorea	11	0.564	0.115	0.270	0.118	0.025	0.118
19	Dysgonomonas	1	0.000	0.024	0.000	3.43E-18	0.011	5.71E-18
20	Eggerthella	11	0.891	0.114	0.023	0.198	0.022	0.198
21	Eubacterium	2	1.000	0.106	0.000	0.014	0.008	0.014
22	Faecalibacterium	2	0.000	0.107	0.001	0.031	0.007	0.031
23	Finegoldia	6	0.933	0.110	0.006	0.053	0.015	0.053
24	Heliobacterium	14	0.670	0.115	0.554	0.223	0.030	0.223
25	Holdemania	13	0.641	0.114	0.492	0.205	0.028	0.205
26	Mitsuokella	7	1.000	0.110	0.000	0.135	0.015	0.135
27	Oribacterium	16	0.517	0.117	0.488	0.238	0.033	0.238
28	Parabacteroides	1	0.000	0.022	0.000	1.02E-18	0.022	4.00E-18
29	Paraprevotella	3	0.667	0.024	0.013	0.000	0.026	1.37E-17
30	Peptoniphilus	11	0.509	0.114	0.329	0.106	0.025	0.106
31	Peptostreptococcus	6	0.600	0.113	0.016	0.101	0.014	0.101
32	Porphyromonas	3	0.333	0.024	0.040	3.63E-18	0.027	1.37E-17
33	Prevotella	3	0.667	0.024	0.013	0.000	0.026	1.37E-17
34	Pseudoflavonifractor	10	0.778	0.113	0.186	0.166	0.022	0.166
35	Pseudoramibacter	20	0.479	0.118	0.842	0.294	0.040	0.294
36	Pyramidobacter	17	0.603	0.117	0.340	0.273	0.034	0.273
37	Roseburia	1	0.000	0.100	0.000	0.004	0.006	0.004
38	Ruminococcus	7	0.714	0.110	0.081	0.072	0.018	0.072
39	Selenomonas	13	0.756	0.115	0.082	0.226	0.026	0.226
40	Shuttleworthia	16	0.517	0.117	0.554	0.251	0.032	0.251
41	Slackia	16	0.625	0.116	0.284	0.257	0.032	0.257
42	Solobacterium	16	0.325	0.116	0.795	0.181	0.034	0.181
43	Streptobacillus	6	0.667	0.110	0.022	0.058	0.015	0.058
44	Subdoligranulum	13	0.462	0.115	0.581	0.194	0.028	0.194
45	Symbiobacterium	13	0.744	0.115	0.090	0.220	0.026	0.220
46	Turicibacter	7	0.857	0.111	0.013	0.061	0.017	0.061

Mutual exclusion network

Node_Index	Label	Degree	Cluster_Coeff	Closeness	Betweenness	Eigen_Vector	PageRank	HubScore

1	Abiotrophia	1	0.000	0.471	0.000	0.154	0.021	0.062
2	Alistipes	1	0.000	0.353	0.000	0.042	0.022	0.057
3	Anaerococcus	1	0.000	0.471	0.000	0.154	0.021	0.062
4	Anaerofustis	1	0.000	0.471	0.000	0.154	0.021	0.062
5	Anaerostipes	2	0.000	0.490	0.038	0.171	0.037	0.069
6	Bacteroides	20	0.000	0.857	1.000	0.696	0.357	0.944
7	Butyrivibrio	1	0.000	0.471	0.000	0.154	0.021	0.062
8	Catenibacterium	1	0.000	0.471	0.000	0.154	0.021	0.062
9	Clostridium	2	0.000	0.490	0.042	0.170	0.037	0.068
10	Collinsella	1	0.000	0.471	0.000	0.154	0.021	0.062
11	Coprococcus	2	0.000	0.490	0.042	0.170	0.037	0.068
12	Dorea	1	0.000	0.471	0.000	0.154	0.021	0.062
13	Dysgonomonas	1	0.000	0.353	0.000	0.042	0.022	0.057
14	Mitsuokella	1	0.000	0.471	0.000	0.154	0.021	0.062
15	Oribacterium	1	0.000	0.471	0.000	0.154	0.021	0.062
16	Parabacteroides	2	0.000	0.343	0.002	0.075	0.038	0.102
17	Paraprevotella	2	0.000	0.364	0.006	0.079	0.038	0.108
18	Peptoniphilus	1	0.000	0.471	0.000	0.154	0.021	0.062
19	Pseudoramibacter	1	0.000	0.471	0.000	0.154	0.021	0.062
20	Pyramidobacter	1	0.000	0.471	0.000	0.154	0.021	0.062
21	Ruminococcus	4	0.000	0.533	0.208	0.189	0.074	0.076
22	Selenomonas	1	0.000	0.471	0.000	0.154	0.021	0.062
23	Shuttleworthia	1	0.000	0.471	0.000	0.154	0.021	0.062
24	Solobacterium	1	0.000	0.471	0.000	0.154	0.021	0.062
25	Subdoligranulum	1	0.000	0.471	0.000	0.154	0.021	0.062

Co-occurrence network

Node_Index	Label	Degree	Cluster_Coeff	Closeness	Betweenness	Eigen_Vector	PageRank	HubScore
1	Abiotrophia	1	0.000	0.146	0.000	0.014	0.005	0.014
2	Acetivibrio	14	0.945	0.167	0.003	0.206	0.024	0.206
3	Acholeplasma	19	0.637	0.177	0.305	0.247	0.033	0.247
4	Aerococcus	6	0.733	0.161	0.004	0.086	0.012	0.086
5	Alicyclobacillus	16	0.583	0.177	0.367	0.172	0.029	0.172
6	Alkaliphilus	19	0.632	0.180	0.470	0.256	0.032	0.256
7	Anaerococcus	15	0.819	0.168	0.303	0.207	0.028	0.207
8	Anaerofustis	14	0.703	0.177	0.254	0.196	0.025	0.196
9	Anaerotruncus	1	0.000	0.144	0.000	0.005	0.005	0.005
10	Atopobium	1	0.000	0.146	0.000	0.014	0.005	0.014
11	Bifidobacterium	2	1.000	0.145	0.000	0.007	0.007	0.007
12	Caldicellulosiruptor	17	0.743	0.178	0.180	0.238	0.029	0.238
13	Capnocytophaga	2	1.000	0.024	0.000	6.68E-18	0.022	0.000
14	Collinsella	6	0.733	0.152	0.042	0.037	0.015	0.037
15	Dehalococcoides	1	0.000	0.154	0.000	0.010	0.005	0.010
16	Desulfitobacterium	7	1.000	0.164	0.000	0.067	0.015	0.067
17	Eggerthella	13	0.808	0.169	0.079	0.108	0.025	0.108
18	Fibrobacter	3	0.667	0.158	0.001	0.038	0.008	0.038
19	Finegoldia	15	0.857	0.168	0.020	0.214	0.026	0.214
20	Fusobacterium	14	0.879	0.167	0.018	0.199	0.025	0.199
21	Gemella	14	0.901	0.174	0.050	0.209	0.024	0.209
22	Heliobacterium	17	0.574	0.179	0.584	0.145	0.034	0.145
23	Holdemania	4	1.000	0.161	0.000	0.041	0.010	0.041
24	Jonquetella	10	0.756	0.173	0.150	0.096	0.020	0.096
25	Mesoplasma	19	0.632	0.180	0.350	0.251	0.033	0.251
26	Mitsuokella	9	1.000	0.165	0.000	0.085	0.018	0.085
27	Paraprevotella	2	1.000	0.024	0.000	4.74E-20	0.022	0.000
28	Parascardovia	12	0.909	0.167	0.006	0.107	0.023	0.107
29	Peptoniphilus	15	0.819	0.168	0.303	0.207	0.028	0.207
30	Peptostreptococcus	2	1.000	0.154	0.000	0.033	0.006	0.033
31	Porphyromonas	1	0.000	0.024	0.000	1.13E-18	0.013	0.000
32	Prevotella	3	0.333	0.024	0.015	2.17E-17	0.033	0.000
33	Pseudoflavonifractor	11	0.582	0.165	0.564	0.070	0.026	0.070
34	Pseudoramibacter	15	0.733	0.171	0.104	0.118	0.029	0.118
35	Pyramidobacter	16	0.667	0.171	0.149	0.121	0.031	0.121
36	Selenomonas	23	0.435	0.183	1.000	0.237	0.040	0.237
37	Shuttleworthia	12	0.818	0.167	0.022	0.103	0.023	0.103
38	Slackia	15	0.733	0.171	0.104	0.118	0.029	0.118
39	Solobacterium	13	0.795	0.175	0.134	0.192	0.023	0.192
40	Streptobacillus	13	0.897	0.167	0.013	0.189	0.023	0.189
41	Symbiobacterium	15	0.676	0.177	0.271	0.140	0.028	0.140
42	Thermanaerovibrio	17	0.559	0.179	0.423	0.152	0.031	0.152
43	Thermoanaerobacter	16	0.750	0.174	0.177	0.218	0.028	0.218
44	Treponema	10	0.467	0.172	0.161	0.123	0.019	0.123
45	Turicibacter	18	0.667	0.177	0.278	0.239	0.032	0.239

Mutual exclusion network

Node_Index	Label	Degree	Cluster_Coeff	Closeness	Betweenness	Eigen_Vector	PageRank	HubScore
1	Acholeplasma	1	0.000	0.131	0.000	0.204	0.030	0.288

2	Alistipes	1	0.000	0.056	0.000	3.41E-17	0.053	0.000
3	Anaerococcus	1	0.000	0.131	0.000	0.204	0.030	0.288
4	Anaerofustis	1	0.000	0.131	0.000	0.204	0.030	0.288
5	Anaerostipes	1	0.000	0.056	0.000	9.22E-18	0.053	0.000
6	Bacteroides	12	0.000	0.143	1.000	0.707	0.319	0.084
7	Butyrivibrio	1	0.000	0.131	0.000	0.204	0.030	0.288
8	Dialister	1	0.000	0.056	0.000	1.16E-17	0.053	0.000
9	Finegoldia	1	0.000	0.131	0.000	0.204	0.030	0.288
10	Gemella	1	0.000	0.131	0.000	0.204	0.030	0.288
11	Heliobacterium	1	0.000	0.131	0.000	0.204	0.030	0.288
12	Mesoplasma	1	0.000	0.131	0.000	0.204	0.030	0.288
13	Oribacterium	1	0.000	0.131	0.000	0.204	0.030	0.288
14	Phascolarctobacterium	1	0.000	0.056	0.000	8.78E-18	0.053	0.000
15	Prevotella	1	0.000	0.056	0.000	1.75E-17	0.053	0.000
16	Pseudoramibacter	1	0.000	0.131	0.000	0.204	0.030	0.288
17	Selenomonas	1	0.000	0.131	0.000	0.204	0.030	0.288
18	Subdoligranulum	1	0.000	0.056	0.000	0.000	0.053	0.000
19	Thermoanaerobacter	1	0.000	0.131	0.000	0.204	0.030	0.288

Co-occurrence network

Node_Index	Label	Degree	Cluster_Coeff	Closeness	Betweenness	Eigen_Vector	PageRank	HubScore
1	Acetivibrio	1	0.000	0.046	0.000	6.60E-17	0.009	4.71E-17
2	Acholeplasma	3	1.000	0.047	0.000	2.16E-16	0.018	8.92E-17
3	Aerococcus	1	0.000	0.047	0.000	7.55E-17	0.009	6.48E-17
4	Alicyclobacillus	4	1.000	0.051	0.000	0.209	0.024	0.209
5	Alkaliphilus	5	0.800	0.047	0.027	2.96E-16	0.027	2.17E-16
6	Anaerococcus	8	0.643	0.047	0.181	6.18E-16	0.041	2.35E-16
7	Bifidobacterium	1	0.000	0.050	0.000	0.008	0.013	0.008
8	Caldicellulosiruptor	7	0.810	0.047	0.036	4.16E-16	0.035	2.78E-16
9	Capnocytophaga	2	1.000	0.032	0.000	0.000	0.029	1.16E-17
10	Collinsella	2	0.000	0.051	0.438	0.053	0.021	0.053
11	Desulfitobacterium	2	1.000	0.051	0.000	0.106	0.014	0.106
12	Eggerthella	6	1.000	0.052	0.000	0.312	0.034	0.312
13	Finegoldia	7	0.667	0.047	0.380	4.03E-16	0.038	2.78E-16
14	Fusobacterium	8	0.571	0.047	0.359	4.74E-16	0.041	2.35E-16
15	Gemella	5	0.500	0.047	0.083	3.43E-16	0.028	1.22E-16
16	Heliobacterium	8	0.679	0.052	0.227	0.352	0.045	0.352
17	Holdemania	1	0.000	0.051	0.000	0.052	0.009	0.052
18	Jonquetella	1	0.000	0.051	0.000	0.056	0.009	0.056
19	Mesoplasma	4	0.833	0.047	0.014	2.81E-16	0.023	1.41E-16
20	Mitsuokella	2	1.000	0.051	0.000	0.103	0.014	0.103
21	Paraprevotella	2	1.000	0.032	0.000	0.000	0.029	1.16E-17
22	Peptoniphilus	2	1.000	0.047	0.000	1.24E-16	0.014	7.06E-17
23	Porphyromonas	1	0.000	0.032	0.000	0.000	0.017	5.78E-18
24	Prevotella	3	0.333	0.032	0.063	0.000	0.043	1.84E-17
25	Pseudoflavonifractor	1	0.000	0.051	0.000	0.050	0.009	0.050
26	Pseudoramibacter	8	0.750	0.052	0.102	0.365	0.044	0.365
27	Pyramidobacter	10	0.489	0.052	0.742	0.385	0.058	0.385
28	Selenomonas	9	0.528	0.052	0.625	0.358	0.053	0.358
29	Shuttleworthia	4	1.000	0.052	0.000	0.212	0.024	0.212
30	Slackia	9	0.528	0.052	1.000	0.358	0.053	0.358
31	Streptobacillus	6	0.733	0.047	0.063	4.58E-16	0.031	2.26E-16
32	Symbiobacterium	8	0.643	0.052	0.492	0.345	0.046	0.345
33	Thermoanaerobacter	10	0.467	0.047	0.638	6.13E-16	0.053	2.45E-16
34	Turicibacter	9	0.611	0.047	0.217	4.83E-16	0.045	2.68E-16

Mutual exclusion network

Node_Index	Label	Degree	Cluster_Coeff	Closeness	Betweenness	Eigen_Vector	PageRank	HubScore
1	Anaerococcus	1	0.000	0.286	0.000	0.354	0.094	0.224
2	Bacteroides	4	0.000	0.333	1.000	0.707	0.340	0.894
3	Dialister	1	0.000	0.167	0.000	2.58E-17	0.143	0.000
4	Finegoldia	1	0.000	0.286	0.000	0.354	0.094	0.224
5	Phascolarctobacterium	1	0.000	0.167	0.000	9.30E-18	0.143	0.000
6	Selenomonas	1	0.000	0.286	0.000	0.354	0.094	0.224
7	Thermoanaerobacter	1	0.000	0.286	0.000	0.354	0.094	0.224

Co-occurrence network

Node_Index	Label	Degree	Cluster_Coeff	Closeness	Betweenness	Eigen_Vector	PageRank	HubScore
1	Alistipes	3	0.000	0.115	0.813	0.016	0.035	0.016
2	Anaerococcus	6	0.800	0.117	0.026	0.022	0.034	0.022
3	Anaerofustis	4	1.000	0.115	0.000	0.012	0.024	0.012
4	Anaerotruncus	5	0.600	0.123	1.000	0.143	0.029	0.143
5	Atopobium	1	0.000	0.114	0.000	0.006	0.010	0.006
6	Bilophila	2	0.000	0.106	0.288	0.002	0.029	0.002
7	Capnocytophaga	2	1.000	0.036	0.000	0.000	0.032	1.81E-18
8	Collinsella	5	1.000	0.120	0.000	0.175	0.022	0.175
9	Dorea	1	0.000	0.033	0.000	7.80E-19	0.032	9.05E-19
10	Dysgonomonas	1	0.000	0.106	0.000	0.002	0.015	0.002
11	Eggerthella	10	0.778	0.128	0.249	0.309	0.041	0.309
12	Eubacterium	1	0.000	0.033	0.000	0.000	0.032	9.05E-19
13	Finegoldia	5	0.900	0.117	0.010	0.021	0.029	0.021
14	Heliobacterium	9	0.917	0.126	0.011	0.298	0.036	0.298
15	Holdemania	3	1.000	0.122	0.000	0.104	0.015	0.104
16	Mitsuokella	6	1.000	0.122	0.000	0.208	0.026	0.208
17	Parabacteroides	1	0.000	0.098	0.000	0.000	0.017	0.000
18	Paraprevotella	2	1.000	0.036	0.000	0.000	0.032	1.81E-18
19	Peptoniphilus	8	0.429	0.124	0.896	0.051	0.046	0.051
20	Porphyromonas	1	0.000	0.036	0.000	0.000	0.018	4.24E-18
21	Prevotella	3	0.333	0.036	0.025	0.000	0.047	0.000
22	Pseudoflavonifractor	10	0.778	0.128	0.249	0.309	0.041	0.309
23	Pseudoramibacter	10	0.822	0.126	0.045	0.316	0.040	0.316
24	Pyramidobacter	12	0.636	0.129	0.402	0.340	0.049	0.340
25	Selenomonas	11	0.655	0.128	0.338	0.316	0.045	0.316
26	Shuttleworthia	10	0.667	0.129	0.910	0.293	0.042	0.293
27	Slackia	11	0.727	0.129	0.295	0.330	0.045	0.330
28	Solobacterium	6	0.467	0.124	0.515	0.081	0.031	0.081
29	Streptobacillus	5	0.900	0.115	0.003	0.014	0.029	0.014
30	Symbiobacterium	10	0.756	0.128	0.275	0.306	0.041	0.306
31	Turicibacter	6	0.800	0.117	0.026	0.022	0.034	0.022

Mutual exclusion network

Node_Index	Label	Degree	Cluster_Coeff	Closeness	Betweenness	Eigen_Vector	PageRank	HubScore
1	Anaerococcus	1	0.000	0.276	0.000	0.289	0.069	0.135
2	Anaerofustis	1	0.000	0.276	0.000	0.289	0.069	0.135
3	Bacteroides	6	0.000	0.333	1.000	0.707	0.366	0.944
4	Butyrivibrio	1	0.000	0.276	0.000	0.289	0.069	0.135
5	Faecalibacterium	1	0.000	0.125	0.000	3.67E-17	0.111	0.000
6	Fusobacterium	1	0.000	0.125	0.000	6.79E-17	0.111	0.000
7	Oribacterium	1	0.000	0.276	0.000	0.289	0.069	0.135
8	Pseudoramibacter	1	0.000	0.276	0.000	0.289	0.069	0.135
9	Selenomonas	1	0.000	0.276	0.000	0.289	0.069	0.135

Co-occurrence network

Node_Index	Label	Degree	Cluster_Coeff	Closeness	Betweenness	Eigen_Vector	PageRank	HubScore
1	Abiotrophia	2	0.000	0.062	0.281	0.001	0.020	0.001
2	Alistipes	9	0.444	0.067	0.417	0.253	0.035	0.253
3	Anaerofustis	1	0.000	0.059	0.000	0.000	0.012	0.000
4	Anaerostipes	1	0.000	0.026	0.000	0.000	0.019	0.000
5	Anaerotruncus	10	0.622	0.067	0.058	0.331	0.036	0.331
6	Bacteroides	2	0.000	0.026	0.011	0.000	0.036	0.000
7	Bilophila	1	0.000	0.026	0.000	0.000	0.019	0.000
8	Blautia	1	0.000	0.025	0.000	0.000	0.024	0.000
9	Bulleidia	5	0.400	0.067	1.000	0.141	0.023	0.141
10	Butyrivibrio	2	0.000	0.062	0.540	0.002	0.021	0.002
11	Capnocytophaga	2	1.000	0.026	0.000	0.000	0.024	0.000
12	Catenibacterium	1	0.000	0.062	0.000	0.003	0.011	0.003
13	Clostridium	2	0.000	0.026	0.011	0.000	0.036	0.000
14	Collinsella	6	0.867	0.066	0.006	0.224	0.022	0.224
15	Coprobacillus	1	0.000	0.025	0.000	0.000	0.024	0.000
16	Coprococcus	3	0.333	0.065	0.199	0.027	0.018	0.027
17	Dorea	1	0.000	0.058	0.000	3.17E-05	0.014	3.17E-05
18	Dysgonomonas	3	0.667	0.065	0.015	0.072	0.015	0.072
19	Eubacterium	2	0.000	0.060	0.281	0.000	0.025	0.000
20	Faecalibacterium	4	0.167	0.066	0.598	0.059	0.022	0.059
21	Heliobacterium	11	0.600	0.067	0.104	0.356	0.039	0.356
22	Holdemania	8	0.500	0.067	0.426	0.242	0.031	0.242
23	Megasphaera	1	0.000	0.025	0.000	0.000	0.024	0.000
24	Mitsuokella	1	0.000	0.025	0.000	0.000	0.024	0.000
25	Oribacterium	6	0.333	0.067	0.379	0.150	0.025	0.150
26	Parabacteroides	1	0.000	0.026	0.000	0.000	0.019	0.000
27	Paraprevotella	2	1.000	0.026	0.000	0.000	0.024	0.000
28	Peptostreptococcus	2	0.000	0.064	0.778	0.017	0.017	0.017
29	Porphyromonas	2	1.000	0.064	0.000	0.039	0.011	0.039
30	Prevotella	2	1.000	0.026	0.000	0.000	0.024	0.000
31	Pseudoflavonifractor	10	0.533	0.067	0.217	0.301	0.037	0.301
32	Pseudoramibacter	7	0.524	0.067	0.266	0.215	0.027	0.215
33	Pyramidobacter	11	0.527	0.068	0.462	0.337	0.040	0.337
34	Roseburia	3	0.333	0.064	0.540	0.011	0.022	0.011
35	Ruminococcus	1	0.000	0.026	0.000	0.000	0.019	0.000
36	Selenomonas	8	0.500	0.067	0.585	0.229	0.033	0.229
37	Shuttleworthia	10	0.556	0.068	0.360	0.313	0.037	0.313
38	Slackia	3	1.000	0.065	0.000	0.111	0.013	0.111
39	Solobacterium	2	0.000	0.065	0.281	0.028	0.016	0.028
40	Subdoligranulum	7	0.429	0.067	0.423	0.210	0.027	0.210
41	Symbiobacterium	9	0.583	0.067	0.267	0.281	0.033	0.281

Mutual exclusion network

No common nodes in mutual exclusion network

Co-occurrence network

Node_Index	Label	Degree	Cluster_Coeff	Closeness	Betweenness	Eigen_Vector	PageRank	HubScore
1	Alistipes	2	0.000	0.106	0.019	0.157	0.035	0.157
2	Anaerotruncus	3	0.333	0.114	0.278	0.329	0.047	0.329
3	Bacteroides	1	0.000	0.048	0.000	1.49E-17	0.045	4.04E-18
4	Butyrivibrio	1	0.000	0.050	0.000	1.70E-18	0.035	0.000
5	Clostridium	1	0.000	0.048	0.000	7.81E-18	0.045	4.04E-18
6	Coprococcus	1	0.000	0.100	0.000	0.018	0.025	0.018
7	Dorea	1	0.000	0.050	0.000	1.68E-17	0.035	0.000
8	Dysgonomonas	2	0.000	0.110	0.092	0.203	0.035	0.203
9	Eubacterium	2	0.000	0.050	0.019	1.67E-17	0.066	0.000
10	Faecalibacterium	3	0.000	0.112	0.468	0.190	0.053	0.190
11	Heliobacterium	4	0.167	0.112	0.364	0.328	0.066	0.328
12	Holdemania	4	0.000	0.112	0.313	0.223	0.068	0.223
13	Oribacterium	2	0.000	0.110	0.013	0.172	0.035	0.172
14	Parabacteroides	1	0.000	0.048	0.000	1.06E-17	0.045	4.04E-18
15	Pseudoflavonifractor	6	0.200	0.117	1.000	0.532	0.090	0.532
16	Pseudoramibacter	1	0.000	0.104	0.000	0.097	0.021	0.097
17	Pyramidobacter	3	0.667	0.111	0.025	0.328	0.048	0.328
18	Roseburia	2	0.000	0.106	0.247	0.061	0.043	0.061
19	Ruminococcus	1	0.000	0.048	0.000	1.46E-17	0.045	4.04E-18
20	Selenomonas	1	0.000	0.105	0.000	0.066	0.021	0.066
21	Slackia	2	1.000	0.109	0.000	0.254	0.033	0.254
22	Subdoligranulum	4	0.167	0.115	0.598	0.361	0.063	0.361

Mutual exclusion network

No common nodes in mutual exclusion network

Co-occurrence network

Node_Index	Label	Degree	Cluster_Coeff	Closeness	Betweenness	Eigen_Vector	PageRank	HubScore
1	Acetivibrio	2	0.000	0.043	0.003	0.076	0.012	0.076
2	Acholeplasma	3	1.000	0.043	0.000	0.119	0.016	0.119
3	Aerococcus	1	0.000	0.043	0.000	0.051	0.008	0.051
4	Alicyclobacillus	5	0.700	0.049	0.074	3.62E-16	0.027	0.000
5	Alkaliphilus	6	0.800	0.043	0.027	0.254	0.026	0.254
6	Anaerococcus	9	0.611	0.043	0.136	0.341	0.038	0.341
7	Bifidobacterium	1	0.000	0.047	0.000	7.75E-18	0.012	1.07E-17
8	Caldicellulosiruptor	8	0.786	0.043	0.025	0.337	0.034	0.337
9	Capnocytophaga	2	1.000	0.029	0.000	0.000	0.027	2.15E-17
10	Collinsella	2	0.000	0.048	0.309	9.27E-17	0.019	0.000
11	Desulfitobacterium	2	1.000	0.048	0.000	1.46E-16	0.013	0.000
12	Eggerthella	6	1.000	0.049	0.000	5.12E-16	0.031	0.000
13	Finegoldia	7	0.667	0.043	0.119	0.283	0.031	0.283
14	Fusobacterium	9	0.583	0.043	0.245	0.336	0.039	0.336
15	Gemella	5	0.500	0.043	0.051	0.174	0.024	0.174
16	Heliobacterium	8	0.679	0.049	0.140	5.07E-16	0.041	0.000
17	Holdemania	1	0.000	0.048	0.000	8.92E-17	0.009	0.000
18	Jonquetella	2	0.000	0.049	0.579	8.45E-17	0.019	0.000
19	Leptotrichia	7	0.667	0.043	0.120	0.283	0.031	0.283
20	Magnetospirillum	2	0.000	0.048	0.309	4.08E-18	0.024	3.35E-17
21	Mesoplasma	4	0.833	0.043	0.009	0.163	0.019	0.163
22	Mitsuokella	2	1.000	0.048	0.000	1.56E-16	0.013	0.000
23	Paraprevotella	2	1.000	0.029	0.000	0.000	0.027	2.15E-17
24	Peptoniphilus	2	1.000	0.043	0.000	0.069	0.012	0.069
25	Porphyromonas	1	0.000	0.029	0.000	0.000	0.015	4.73E-18
26	Prevotella	3	0.333	0.029	0.039	0.000	0.040	3.22E-17
27	Pseudoflavonifractor	2	1.000	0.048	0.000	1.19E-16	0.013	0.000
28	Pseudoramibacter	8	0.750	0.049	0.064	6.15E-16	0.040	0.000
29	Pyramidobacter	10	0.489	0.049	1.000	6.31E-16	0.053	0.000
30	Rhodospirillum	1	0.000	0.047	0.000	0.000	0.014	1.68E-17
31	Selenomonas	9	0.528	0.049	0.442	5.67E-16	0.048	0.000
32	Shuttleworthia	4	1.000	0.049	0.000	3.26E-16	0.022	0.000
33	Slackia	9	0.528	0.049	0.712	5.43E-16	0.049	0.000
34	Streptobacillus	6	0.733	0.043	0.035	0.254	0.026	0.254
35	Symbiobacterium	8	0.679	0.049	0.249	5.68E-16	0.041	0.000
36	Thermoanaerobacter	11	0.473	0.043	0.432	0.377	0.049	0.377
37	Turicibacter	10	0.600	0.043	0.149	0.377	0.042	0.377

Mutual exclusion network

Node_Index	Label	Degree	Cluster_Coeff	Closeness	Betweenness	Eigen_Vector	PageRank	HubScore
1	Anaerococcus	1	0.000	0.286	0.000	0.354	0.094	0.224
2	Bacteroides	4	0.000	0.333	1.000	0.707	0.340	0.894
3	Dialister	1	0.000	0.167	0.000	2.58E-17	0.143	0.000
4	Finegoldia	1	0.000	0.286	0.000	0.354	0.094	0.224
5	Phascolarctobacterium	1	0.000	0.167	0.000	9.30E-18	0.143	0.000
6	Selenomonas	1	0.000	0.286	0.000	0.354	0.094	0.224
7	Thermoanaerobacter	1	0.000	0.286	0.000	0.354	0.094	0.224
