# Supplementary material for: Global investigation of composition and interaction networks in gut microbiomes of individuals belonging to diverse geographies and age-groups
Source: Gut Pathog. 2016 May 6;8:17. doi: 10.1186/s13099-016-0099-z (PMC4858888; doi:10.1186/s13099-016-0099-z)
Supplement: Supplementary file 12 — 10.1186/s13099-016-0099-z Order-level affiliations of the various genera constituting the gut microbial networks of different geographies, along with the number of co-occurrence and mutual exclusion interactions between them. [file 13099_2016_99_MOESM12_ESM.pdf]

Additional File 12: Order-level affiliations of the various genera constituting the gut microbial networks of different geographies, along with the number of co-occurrence and mutual exclusion interactions between them

Comparison of relationships among taxonomic groups at order level in the microbial networks across different geographies

Given the preference of specific bacterial genera to exist either in the co-occurrence or the mutual exclusion network of the gut microbiomes, the relationships between various bacterial groups in the guts of individuals from different geographies were investigated. The objective was to identify common trends of interactions that exist amongst the different bacterial groups across gut microbiomes of individuals belonging to different nationalities. For this purpose, the order-level affiliations of the various genera constituting the gut microbial networks were obtained for the different geographies, along with the number of co-occurrence and mutual exclusions among them. These were subsequently compared across different nationalities as summarized in the tables below.

In the gut microbiome of the American individuals, 126 interactions out of 866 co-occurrence relations were observed to be within genera belonging to the order Clostridiales. Clostridiales also showed a noticeably high number of positive interactions with Erysipelotrichales, Coriobacteriales, Thermoanaerobacterales, Synergistales, Bifidobacteriales, Selenomonadales, Bacillales, Lactobacillales (all more than 20 interactions). On the other hand, as observed in the previous section for the constituent genera Bacteroides, the corresponding order Bacteroidales was observed to serve as the hub for mutual exclusion relationships. Of these, the maximum number of mutual exclusion relationships (41) was found to be with the Clostridiales order.

Individuals from Denmark, Spain and China were found to have similar trend with Clostridiales order showing maximum co-occurrence interactions amongst them as well as with Synergistales, Erysipelotrichales, Fusobacteriales, Selenomonadales, Coriobacteriales (in Denmark), Clostridiales, Coriobacteriales, Thermoanaerobacterales (in Spain) and Erysipelotrichales, Coriobacteriales (in China). As in the American population, the pattern of mutual exclusion interactions also showed similar patterns (for these regions), with most of them again formed by Bacteroidales with Clostridiales. These results indicate the overall similarities in the taxonomic composition of the gut microbial co-occurrence/co-exclusion networks across different geographies.

The current study detected the largest number of co-occurrence relations between genera belonging to the same taxonomic order (Clostridiales v/s Clostridiales). Genera belonging to Clostridiales (phylum Firmicutes) were also observed to have large number of co-occurrence relationships with genera belonging to Ruminococcales, Lactobacillales and Erysipelotrichales (all belonging to the phylum Firmicutes). The gene content of bacterial species showed progressive similarity with decreasing phylogenetic distance. Consequently, bacterial groups belonging to the same taxonomic clades are expected to have similarities in their gene content and hence metabolic potential (consequently their nutrient requirements). The increased co-occurrence relationships between taxonomically similar genera could thus be a consequence of the above observation. The similarity in metabolic profile as one of the reasons for co-occurrence is further evident in the investigation of the gut microbial interaction networks across individuals from various age-groups.

Co-occurrence network

Order	Clostridiales	Erysipelotrichales	Coriobacteriales	Synergistales	Thermoanaerobacterales	Lactobacillales	Bifidobacteriales
**Clostridiales**	122	68	56	36	39	22	29
**Erysipelotrichales**	68	4	13	7	9	6	3
**Coriobacteriales**	56	13	6	11	9	4	7
**Synergistales**	36	7	11	3	8	3	5
**Thermoanaerobacterales**	39	9	9	8	3	3	5
**Lactobacillales**	22	6	4	3	3	0	3
**Bifidobacteriales**	29	3	7	5	5	3	1
**Bacillales**	24	5	6	5	6	2	2
**Selenomonadales**	24	8	8	6	6	2	4
**Spirochaetales**	13	4	4	3	3	2	1
**Bacteroidales**	0	0	0	0	0	0	0
**Fusobacteriales**	14	5	3	1	4	2	0
**Acholeplasmatales**	13	3	3	2	3	2	1
**Desulfovibrionales**	10	2	4	2	1	1	1
**Asterales**	12	3	4	3	3	1	1
**Entomoplasmatales**	13	3	3	2	3	2	1
**Fibrobacterales**	8	3	4	3	3	1	1
**Aeromonadales**	5	0	0	0	1	0	0
**Flavobacteriales**	0	0	0	0	0	0	0
**Dehalococcoidales**	5	1	1	1	1	1	0
**Burkholderiales**	5	1	1	2	2	0	1
**Verrucomicrobiales**	2	0	0	0	0	0	0
**Cytophagales**	0	0	0	0	0	0	1
**Actinomycetales**	2	0	1	0	0	0	0
**Sphingobacteriales**	0	0	0	0	0	0	0

Order	Bacillales	Selenomonadales	Spirochaetales	Bacteroidales	Fusobacteriales	Acholeplasmatales	Desulfovibrionales
**Clostridiales**	24	24	13	0	14	13	10
**Erysipelotrichales**	5	8	4	0	5	3	2
**Coriobacteriales**	6	8	4	0	3	3	4
**Synergistales**	5	6	3	0	1	2	2
**Thermoanaerobacterales**	6	6	3	0	4	3	1
**Lactobacillales**	2	2	2	0	2	2	1
**Bifidobacteriales**	2	4	1	0	0	1	1
**Bacillales**	1	4	2	0	2	2	1
**Selenomonadales**	4	1	2	0	1	2	2

**Spirochaetales**	2	2	0	0	2	1	1
**Bacteroidales**	0	0	0	13	0	0	0
**Fusobacteriales**	2	1	2	0	1	2	0
**Acholeplasmatales**	2	2	1	0	2	0	0
**Desulfovibrionales**	1	2	1	0	0	0	0
**Asterales**	2	2	1	0	1	1	1
**Entomoplasmatales**	2	2	1	0	2	1	0
**Fibrobacterales**	2	2	1	0	1	1	0
**Aeromonadales**	0	0	1	0	0	0	0
**Flavobacteriales**	0	0	0	5	0	0	0
**Dehalococcoidales**	1	0	1	0	0	0	0
**Burkholderiales**	1	1	1	1	1	1	0
**Verrucomicrobiales**	0	0	0	0	0	1	0
**Cytophagales**	0	0	0	3	0	0	0
**Actinomycetales**	0	0	0	0	0	0	0
**Sphingobacteriales**	0	0	0	1	0	0	0

Order	Asterales	Entomoplasmatales	Fibrobacterales	Aeromonadales	Flavobacteriales	Dehalococcoidales	Burkholderiales
**Clostridiales**	12	13	8	5	0	5	5
**Erysipelotrichales**	3	3	3	0	0	1	1
**Coriobacteriales**	4	3	4	0	0	1	1
**Synergistales**	3	2	3	0	0	1	2
**Thermoanaerobacterales**	3	3	3	1	0	1	2
**Lactobacillales**	1	2	1	0	0	1	0
**Bifidobacteriales**	1	1	1	0	0	0	1
**Bacillales**	2	2	2	0	0	1	1
**Selenomonadales**	2	2	2	0	0	0	1
**Spirochaetales**	1	1	1	1	0	1	1
**Bacteroidales**	0	0	0	0	5	0	1
**Fusobacteriales**	1	2	1	0	0	0	1
**Acholeplasmatales**	1	1	1	0	0	0	1
**Desulfovibrionales**	1	0	0	0	0	0	0
**Asterales**	0	1	1	0	0	1	1
**Entomoplasmatales**	1	0	1	0	0	0	1
**Fibrobacterales**	1	1	0	0	0	0	1
**Aeromonadales**	0	0	0	0	0	1	0
**Flavobacteriales**	0	0	0	0	0	0	0
**Dehalococcoidales**	1	0	0	1	0	0	0
**Burkholderiales**	1	1	1	0	0	0	0

**Verrucomicrobiales**	0	0	0	0	0	0	0
**Cytophagales**	0	0	1	0	0	0	1
**Actinomycetales**	0	0	0	0	0	0	0
**Sphingobacteriales**	0	0	0	0	1	0	0

Order	Verrucomicrobiales	Cytophagales	Actinomycetales	Sphingobacteriales
**Clostridiales**	2	0	2	0
**Erysipelotrichales**	0	0	0	0
**Coriobacteriales**	0	0	1	0
**Synergistales**	0	0	0	0
**Thermoanaerobacterales**	0	0	0	0
**Lactobacillales**	0	0	0	0
**Bifidobacteriales**	0	1	0	0
**Bacillales**	0	0	0	0
**Selenomonadales**	0	0	0	0
**Spirochaetales**	0	0	0	0
**Bacteroidales**	0	3	0	1
**Fusobacteriales**	0	0	0	0
**Acholeplasmatales**	1	0	0	0
**Desulfovibrionales**	0	0	0	0
**Asterales**	0	0	0	0
**Entomoplasmatales**	0	0	0	0
**Fibrobacterales**	0	1	0	0
**Aeromonadales**	0	0	0	0
**Flavobacteriales**	0	0	0	1
**Dehalococcoidales**	0	0	0	0
**Burkholderiales**	0	1	0	0
**Verrucomicrobiales**	0	0	0	0
**Cytophagales**	0	0	0	1
**Actinomycetales**	0	0	0	0
**Sphingobacteriales**	0	1	0	0

Mutual exclusion network

Order	Selenomonadales	Entomoplasmatales	Cytophagales	Coriobacteriales	Burkholderiales	Lactobacillales	Bacteroidales
**Selenomonadales**	1	1	1	1	1	0	2
**Entomoplasmatales**	1	0	0	0	0	0	1
**Cytophagales**	1	0	0	0	0	0	0

**Coriobacteriales**	1	0	0	0	0	0	4
**Burkholderiales**	1	0	0	0	0	0	0
**Lactobacillales**	0	0	0	0	0	0	1
**Bacteroidales**	2	1	0	4	0	1	0
**Erysipelotrichales**	0	0	0	0	0	0	5
**Clostridiales**	0	0	1	0	2	0	41
**Sphingobacteriales**	0	0	0	0	0	0	0
**Fusobacteriales**	0	0	0	0	0	0	1
**Spirochaetales**	0	0	0	0	0	0	1
**Fibrobacterales**	0	0	0	0	0	0	1
**Desulfovibrionales**	0	0	0	0	0	0	1
**Bacillales**	0	0	0	0	0	0	2
**Asterales**	0	0	0	0	0	0	1
**Aeromonadales**	0	0	0	0	0	0	1
**Acholeplasmatales**	0	0	0	0	0	0	1
**Flavobacteriales**	0	0	0	0	0	0	0
**Thermoanaerobacterales**	0	0	0	0	0	0	2
**Bifidobacteriales**	0	0	0	0	0	0	2
**Synergistales**	0	0	0	0	0	0	3

**Order**	**Erysipelotrichales**	**Clostridiales**	**Sphingobacteriales**	**Fusobacteriales**	**Spirochaetales**	**Fibrobacterales**	**Desulfovibrionales**
**Selenomonadales**	0	0	0	0	0	0	0
**Entomoplasmatales**	0	0	0	0	0	0	0
**Cytophagales**	0	1	0	0	0	0	0
**Coriobacteriales**	0	0	0	0	0	0	0
**Burkholderiales**	0	2	0	0	0	0	0
**Lactobacillales**	0	0	0	0	0	0	0
**Bacteroidales**	5	41	0	1	1	1	1
**Erysipelotrichales**	0	0	0	0	0	0	0
**Clostridiales**	0	4	1	1	0	0	0
**Sphingobacteriales**	0	1	0	0	0	0	0
**Fusobacteriales**	0	1	0	0	0	0	0
**Spirochaetales**	0	0	0	0	0	0	0
**Fibrobacterales**	0	0	0	0	0	0	0
**Desulfovibrionales**	0	0	0	0	0	0	0
**Bacillales**	0	0	0	0	0	0	0
**Asterales**	0	0	0	0	0	0	0
**Aeromonadales**	0	0	0	0	0	0	0
**Acholeplasmatales**	0	1	0	0	0	0	0

**Flavobacteriales**	0	2	0	0	0	0	0
**Thermoanaerobacterales**	0	0	0	0	0	0	0
**Bifidobacteriales**	0	0	0	0	0	0	0
**Synergistales**	0	0	0	0	0	0	0

Order	Bacillales	Asterales	Aeromonadales	Acholeplasmatales	Flavobacteriales	Thermoanaerobacterales	Bifidobacteriales
**Selenomonadales**	0	0	0	0	0	0	0
**Entomoplasmatales**	0	0	0	0	0	0	0
**Cytophagales**	0	0	0	0	0	0	0
**Coriobacteriales**	0	0	0	0	0	0	0
**Burkholderiales**	0	0	0	0	0	0	0
**Lactobacillales**	0	0	0	0	0	0	0
**Bacteroidales**	2	1	1	1	0	2	2
**Erysipelotrichales**	0	0	0	0	0	0	0
**Clostridiales**	0	0	0	1	2	0	0
**Sphingobacteriales**	0	0	0	0	0	0	0
**Fusobacteriales**	0	0	0	0	0	0	0
**Spirochaetales**	0	0	0	0	0	0	0
**Fibrobacterales**	0	0	0	0	0	0	0
**Desulfovibrionales**	0	0	0	0	0	0	0
**Bacillales**	0	0	0	0	0	0	0
**Asterales**	0	0	0	0	0	0	0
**Aeromonadales**	0	0	0	0	0	0	0
**Acholeplasmatales**	0	0	0	0	0	0	0
**Flavobacteriales**	0	0	0	0	0	0	0
**Thermoanaerobacterales**	0	0	0	0	0	0	0
**Bifidobacteriales**	0	0	0	0	0	0	0
**Synergistales**	0	0	0	0	0	0	0

Order	Synergistales
**Selenomonadales**	0
**Entomoplasmatales**	0
**Cytophagales**	0
**Coriobacteriales**	0
**Burkholderiales**	0
**Lactobacillales**	0
**Bacteroidales**	3
**Erysipelotrichales**	0
**Clostridiales**	0

**Sphingobacteriales**	0
**Fusobacteriales**	0
**Spirochaetales**	0
**Fibrobacterales**	0
**Desulfovibrionales**	0
**Bacillales**	0
**Asterales**	0
**Aeromonadales**	0
**Acholeplasmatales**	0
**Flavobacteriales**	0
**Thermoanaerobacterales**	0
**Bifidobacteriales**	0
**Synergistales**	0

Co-occurrence network

Order	Clostridiales	Erysipelotrichales	Coriobacteriales	Lactobacillales	Selenomonadales	Bacteroidales	Synergistales	Asterales
**Clostridiales**	104	58	37	13	17	7	11	6
**Erysipelotrichales**	58	6	13	1	3	1	4	1
**Coriobacteriales**	37	13	6	0	7	2	4	2
**Lactobacillales**	13	1	0	0	0	0	0	0
**Selenomonadales**	17	3	7	0	7	5	4	4
**Bacteroidales**	7	1	2	0	5	10	1	0
**Synergistales**	11	4	4	0	4	1	0	1
**Asterales**	6	1	2	0	4	0	1	0
**Fusobacteriales**	5	2	3	0	0	0	0	0
**Enterobacteriales**	0	0	0	0	2	0	0	0
**Flavobacteriales**	0	0	0	0	0	3	0	0
**Desulfovibrionales**	1	0	0	0	0	4	0	0
**Burkholderiales**	0	0	0	0	0	1	0	0

Order	Fusobacteriales	Enterobacteriales	Flavobacteriales	Desulfovibrionales	Burkholderiales
**Clostridiales**	5	0	0	1	0
**Erysipelotrichales**	2	0	0	0	0
**Coriobacteriales**	3	0	0	0	0
**Lactobacillales**	0	0	0	0	0
**Selenomonadales**	0	2	0	0	0
**Bacteroidales**	0	0	3	4	1
**Synergistales**	0	0	0	0	0
**Asterales**	0	0	0	0	0
**Fusobacteriales**	0	0	0	0	0
**Enterobacteriales**	0	1	0	0	0
**Flavobacteriales**	0	0	0	0	0
**Desulfovibrionales**	0	0	0	0	0
**Burkholderiales**	0	0	0	0	0

Mutual exclusion network

Order	Lactobacillales	Desulfovibrionales	Burkholderiales	Fusobacteriales	Erysipelotrichales	Flavobacteriales	Clostridiales	Coriobacteriales
**Lactobacillales**	0	1	1	0	0	0	0	0
**Desulfovibrionales**	1	0	0	0	0	0	1	0
**Burkholderiales**	1	0	0	0	0	0	0	0

**Fusobacteriales**	0	0	0	0	1	0	3	0
**Erysipelotrichales**	0	0	0	1	0	0	0	0
**Flavobacteriales**	0	0	0	0	0	0	1	0
**Clostridiales**	0	1	0	3	0	1	0	0
**Coriobacteriales**	0	0	0	0	0	0	0	0
**Selenomonadales**	0	0	0	0	0	0	7	1
**Bacteroidales**	2	0	0	0	2	0	29	1
**Synergistales**	0	0	0	0	0	0	0	0
**Enterobacteriales**	2	0	0	0	0	0	14	0

**Order**	**Selenomonadales**	**Bacteroidales**	**Synergistales**	**Enterobacteriales**
**Lactobacillales**	0	2	0	2
**Desulfovibrionales**	0	0	0	0
**Burkholderiales**	0	0	0	0
**Fusobacteriales**	0	0	0	0
**Erysipelotrichales**	0	2	0	0
**Flavobacteriales**	0	0	0	0
**Clostridiales**	7	29	0	14
**Coriobacteriales**	1	1	0	0
**Selenomonadales**	0	3	0	0
**Bacteroidales**	3	0	1	0
**Synergistales**	0	1	0	0
**Enterobacteriales**	0	0	0	0

Co-occurrence network

Order	Clostridiales	Synergistales	Erysipelotrichales	Fusobacteriales	Thermoanaerobacterales	Selenomonadales	Coriobacteriales
**Clostridiales**	40	24	24	20	12	16	16
**Synergistales**	24	3	5	1	3	5	6
**Erysipelotrichales**	24	5	3	8	6	4	0
**Fusobacteriales**	20	1	8	3	6	0	0
**Thermoanaerobacterales**	12	3	6	6	1	1	0
**Selenomonadales**	16	5	4	0	1	3	6
**Coriobacteriales**	16	6	0	0	0	6	3
**Bacillales**	14	2	4	3	2	3	2
**Asterales**	9	3	3	0	2	3	2
**Acholeplasmatales**	8	1	4	3	2	1	0
**Lactobacillales**	11	1	4	4	4	1	0
**Mycoplasmatales**	10	2	3	3	2	1	0
**Entomoplasmatales**	8	1	4	3	2	1	0
**Bifidobacteriales**	8	3	0	0	0	2	3
**Spirochaetales**	7	1	2	4	3	1	0
**Verrucomicrobiales**	3	3	2	1	1	1	2
**Bacteroidales**	3	0	0	0	0	0	0
**Rhodospirillales**	2	1	0	2	0	0	0
**Flavobacteriales**	0	0	0	0	0	0	0
**Desulfovibrionales**	3	2	0	0	0	0	0
**Fibrobacterales**	1	0	0	0	0	0	0
**Dehalococcoidales**	1	0	0	0	0	0	0

Order	Bacillales	Asterales	Acholeplasmatales	Lactobacillales	Mycoplasmatales	Entomoplasmatales	Bifidobacteriales
**Clostridiales**	14	9	8	11	10	8	8
**Synergistales**	2	3	1	1	2	1	3
**Erysipelotrichales**	4	3	4	4	3	4	0
**Fusobacteriales**	3	0	3	4	3	3	0
**Thermoanaerobacterales**	2	2	2	4	2	2	0
**Selenomonadales**	3	3	1	1	1	1	2
**Coriobacteriales**	2	2	0	0	0	0	3
**Bacillales**	0	2	2	3	2	2	1
**Asterales**	2	0	1	1	1	1	1
**Acholeplasmatales**	2	1	0	2	1	1	0
**Lactobacillales**	3	1	2	1	2	1	0
**Mycoplasmatales**	2	1	1	2	0	1	1

**Entomoplasmatales**	2	1	1	1	1	0	0
**Bifidobacteriales**	1	1	0	0	1	0	0
**Spirochaetales**	1	0	2	3	2	2	0
**Verrucomicrobiales**	0	1	0	0	1	0	1
**Bacteroidales**	0	0	0	0	0	0	0
**Rhodospirillales**	0	0	0	2	0	0	0
**Flavobacteriales**	0	0	0	0	0	0	0
**Desulfovibrionales**	1	0	0	0	1	0	1
**Fibrobacterales**	0	0	0	0	0	1	0
**Dehalococcoidales**	0	0	0	0	0	0	0

**Order**	**Spirochaetales**	**Verrucomicrobiales**	**Bacteroidales**	**Rhodospirillales**	**Flavobacteriales**	**Desulfovibrionales**	**Fibrobacterales**
**Clostridiales**	7	3	3	2	0	3	1
**Synergistales**	1	3	0	1	0	2	0
**Erysipelotrichales**	2	2	0	0	0	0	0
**Fusobacteriales**	4	1	0	2	0	0	0
**Thermoanaerobacterales**	3	1	0	0	0	0	0
**Selenomonadales**	1	1	0	0	0	0	0
**Coriobacteriales**	0	2	0	0	0	0	0
**Bacillales**	1	0	0	0	0	1	0
**Asterales**	0	1	0	0	0	0	0
**Acholeplasmatales**	2	0	0	0	0	0	0
**Lactobacillales**	3	0	0	2	0	0	0
**Mycoplasmatales**	2	1	0	0	0	1	0
**Entomoplasmatales**	2	0	0	0	0	0	1
**Bifidobacteriales**	0	1	0	0	0	1	0
**Spirochaetales**	0	0	0	1	0	1	1
**Verrucomicrobiales**	0	0	0	0	0	0	0
**Bacteroidales**	0	0	3	0	2	2	0
**Rhodospirillales**	1	0	0	1	0	2	0
**Flavobacteriales**	0	0	2	0	0	0	0
**Desulfovibrionales**	1	0	2	2	0	0	0
**Fibrobacterales**	1	0	0	0	0	0	0
**Dehalococcoidales**	0	0	0	0	0	0	0

**Order**	**Dehalococcoidales**
**Clostridiales**	1
**Synergistales**	0
**Erysipelotrichales**	0

**Fusobacteriales**	0
**Thermoanaerobacterales**	0
**Selenomonadales**	0
**Coriobacteriales**	0
**Bacillales**	0
**Asterales**	0
**Acholeplasmatales**	0
**Lactobacillales**	0
**Mycoplasmatales**	0
**Entomoplasmatales**	0
**Bifidobacteriales**	0
**Spirochaetales**	0
**Verrucomicrobiales**	0
**Bacteroidales**	0
**Rhodospirillales**	0
**Flavobacteriales**	0
**Desulfovibrionales**	0
**Fibrobacterales**	0
**Dehalococcoidales**	0

Mutual exclusion network

Order	Selenomonadales	Clostridiales	Mycoplasmatales	Entomoplasmatales	Coriobacteriales	Burkholderiales	Synergistales
**Selenomonadales**	1	2	0	0	0	0	0
**Clostridiales**	2	8	1	2	2	0	3
**Mycoplasmatales**	0	1	0	0	0	0	0
**Entomoplasmatales**	0	2	0	0	0	0	0
**Coriobacteriales**	0	2	0	0	0	1	0
**Burkholderiales**	0	0	0	0	1	0	0
**Synergistales**	0	3	0	0	0	0	0
**Bacillales**	0	2	0	0	0	0	0
**Bifidobacteriales**	0	1	0	0	0	0	0
**Bacteroidales**	2	11	0	1	0	0	0
**Thermoanaerobacterales**	0	0	0	0	0	0	0
**Fusobacteriales**	0	2	0	0	0	0	0
**Asterales**	0	1	0	0	0	0	0
**Acholeplasmatales**	0	2	0	0	0	0	0
**Verrucomicrobiales**	0	2	0	0	0	0	0

**Erysipelotrichales**	0	6	0	0	0	0	0

**Order**	**Bacillales**	**Bifidobacteriales**	**Bacteroidales**	**Thermoanaerobacterales**	**Fusobacteriales**	**Asterales**	**Acholeplasmatales**
**Selenomonadales**	0	0	2	0	0	0	0
**Clostridiales**	2	1	11	0	2	1	2
**Mycoplasmatales**	0	0	0	0	0	0	0
**Entomoplasmatales**	0	0	1	0	0	0	0
**Coriobacteriales**	0	0	0	0	0	0	0
**Burkholderiales**	0	0	0	0	0	0	0
**Synergistales**	0	0	0	0	0	0	0
**Bacillales**	0	0	1	0	0	0	0
**Bifidobacteriales**	0	0	1	0	0	0	0
**Bacteroidales**	1	1	3	1	1	1	1
**Thermoanaerobacterales**	0	0	1	0	0	0	0
**Fusobacteriales**	0	0	1	0	0	0	0
**Asterales**	0	0	1	0	0	0	0
**Acholeplasmatales**	0	0	1	0	0	0	0
**Verrucomicrobiales**	0	0	0	0	0	0	0
**Erysipelotrichales**	0	0	0	0	0	0	0

**Order**	**Verrucomicrobiales**	**Erysipelotrichales**
**Selenomonadales**	0	0
**Clostridiales**	2	6
**Mycoplasmatales**	0	0
**Entomoplasmatales**	0	0
**Coriobacteriales**	0	0
**Burkholderiales**	0	0
**Synergistales**	0	0
**Bacillales**	0	0
**Bifidobacteriales**	0	0
**Bacteroidales**	0	0
**Thermoanaerobacterales**	0	0
**Fusobacteriales**	0	0
**Asterales**	0	0
**Acholeplasmatales**	0	0
**Verrucomicrobiales**	0	0
**Erysipelotrichales**	0	0

Co-occurrence network							
**Order**	**Clostridiales**	**Selenomonadales**	**Actinomycetales**	**Thermoanaerobacterales**	**Synergistales**	**Erysipelotrichales**	**Cytophagales**
**Clostridiales**	9	3	3	1	1	2	0
**Selenomonadales**	3	0	1	0	1	0	0
**Actinomycetales**	3	1	0	0	2	0	0
**Thermoanaerobacterales**	1	0	0	0	2	0	0
**Synergistales**	1	1	2	2	1	0	0
**Erysipelotrichales**	2	0	0	0	0	1	0
**Cytophagales**	0	0	0	0	0	0	0
**Rhodospirillales**	0	0	0	0	0	0	2
**Bifidobacteriales**	2	1	0	0	0	0	1
**Bacillales**	3	0	1	1	1	0	1
**Burkholderiales**	0	0	0	0	0	0	0
**Verrucomicrobiales**	0	0	0	0	0	0	0
**Bacteroidales**	1	1	0	0	0	0	0
**Sphaerobacterales**	0	0	0	1	1	0	0
**Spirochaetales**	1	0	0	0	0	1	0
**Cardiobacteriales**	0	0	0	0	0	1	0
**Rubrobacterales**	0	0	0	0	0	0	0
**Opitutales**	0	0	0	0	0	0	1
**Caulobacterales**	0	0	0	0	0	0	0
**Neisseriales**	0	0	0	0	0	0	0
**Methanobacteriales**	0	0	0	0	0	0	0
**Methanomicrobiales**	1	0	1	0	0	0	0
**Flavobacteriales**	1	0	0	0	0	0	0
**Fibrobacterales**	0	0	0	0	0	0	1
**Entomoplasmatales**	0	0	0	0	0	0	0
**Enterobacteriales**	0	0	0	0	0	0	0
**Desulfovibrionales**	0	1	0	0	0	0	0
**Desulfobacterales**	0	0	0	0	0	0	1
**Chromatiales**	0	0	0	1	0	0	1
**Coriobacteriales**	1	0	0	0	0	0	0
**Fusobacteriales**	1	0	0	0	0	0	0
**Bacteroidetes**	1	0	0	0	0	0	0
**Chlorobiales**	1	0	0	0	0	0	0
**Asterales**	0	0	0	0	0	0	0
**Acholeplasmatales**	0	0	0	0	0	0	0

Order	Rhodospirillales	Bifidobacteriales	Bacillales	Burkholderiales	Verrucomicrobiales	Bacteroidales	Sphaerobacterales
Clostridiales	0	2	3	0	0	1	0
Selenomonadales	0	1	0	0	0	1	0
Actinomycetales	0	0	1	0	0	0	0
Thermoanaerobacterales	0	0	1	0	0	0	1
Synergistales	0	0	1	0	0	0	1
Erysipelotrichales	0	0	0	0	0	0	0
Cytophagales	2	1	1	0	0	0	0
Rhodospirillales	0	1	1	0	0	0	0
Bifidobacteriales	1	0	0	0	0	0	0
Bacillales	1	0	0	2	0	0	1
Burkholderiales	0	0	2	0	0	0	0
Verrucomicrobiales	0	0	0	0	0	1	0
Bacteroidales	0	0	0	0	1	0	0
Sphaerobacterales	0	0	1	0	0	0	0
Spirochaetales	0	0	0	0	0	0	0
Cardiobacteriales	0	0	0	0	0	0	0
Rubrobacterales	0	1	0	0	0	0	0
Opitutales	1	1	0	0	0	0	0
Caulobacterales	1	1	0	0	0	0	0
Neisseriales	0	0	0	0	0	0	0
Methanobacteriales	0	0	0	0	0	0	0
Methanomicrobiales	0	0	1	0	0	0	0
Flavobacteriales	0	0	0	0	0	0	0
Fibrobacterales	1	0	1	0	0	0	0
Entomoplasmatales	0	0	0	1	0	0	0
Enterobacteriales	0	0	0	0	0	0	0
Desulfovibrionales	1	1	0	0	0	0	0
Desulfobacterales	1	1	0	0	0	0	0
Chromatiales	0	1	1	0	0	0	0
Coriobacteriales	0	0	0	0	0	1	0
Fusobacteriales	0	0	0	0	0	0	0
Bacteroidetes	0	0	0	0	0	0	0
Chlorobiales	0	0	0	0	0	0	0
Asterales	0	0	0	0	0	1	0
Acholeplasmatales	0	0	0	0	0	0	0

Order	Spirochaetales	Cardiobacteriales	Rubrobacterales	Opitutales	Caulobacterales	Neisseriales	Methanobacteriales
Clostridiales	1	0	0	0	0	0	0

**Selenomonadales**	0	0	0	0	0	0	0
**Actinomycetales**	0	0	0	0	0	0	0
**Thermoanaerobacterales**	0	0	0	0	0	0	0
**Synergistales**	0	0	0	0	0	0	0
**Erysipelotrichales**	1	1	0	0	0	0	0
**Cytophagales**	0	0	0	1	0	0	0
**Rhodospirillales**	0	0	0	1	1	0	0
**Bifidobacteriales**	0	0	1	1	1	0	0
**Bacillales**	0	0	0	0	0	0	0
**Burkholderiales**	0	0	0	0	0	0	0
**Verrucomicrobiales**	0	0	0	0	0	0	0
**Bacteroidales**	0	0	0	0	0	0	0
**Sphaerobacterales**	0	0	0	0	0	0	0
**Spirochaetales**	0	1	0	0	0	0	0
**Cardiobacteriales**	1	0	0	0	0	0	0
**Rubrobacterales**	0	0	0	0	0	0	0
**Opitutales**	0	0	0	0	1	0	0
**Caulobacterales**	0	0	0	1	0	0	0
**Neisseriales**	0	0	0	0	0	0	1
**Methanobacteriales**	0	0	0	0	0	1	0
**Methanomicrobiales**	0	0	0	0	0	0	0
**Flavobacteriales**	0	0	0	0	0	0	0
**Fibrobacterales**	0	0	0	1	0	0	0
**Entomoplasmatales**	0	0	0	0	0	0	0
**Enterobacteriales**	1	0	0	0	0	0	0
**Desulfovibrionales**	0	0	0	1	1	0	0
**Desulfobacterales**	0	0	0	1	1	0	0
**Chromatiales**	0	0	0	0	0	0	0
**Coriobacteriales**	0	0	0	0	0	0	0
**Fusobacteriales**	0	0	0	0	0	0	0
**Bacteroidetes**	0	0	0	0	1	0	0
**Chlorobiales**	0	0	0	0	1	0	0
**Asterales**	0	0	0	0	0	0	0
**Acholeplasmatales**	0	0	0	0	0	0	0

Order	Methanomicrobiales	Flavobacteriales	Fibrobacterales	Entomoplasmatales	Enterobacteriales	Desulfovibrionales	Desulfobacterales
**Clostridiales**	1	1	0	0	0	0	0
**Selenomonadales**	0	0	0	0	0	1	0
**Actinomycetales**	1	0	0	0	0	0	0

**Thermoanaerobacterales**	0	0	0	0	0	0	0
**Synergistales**	0	0	0	0	0	0	0
**Erysipelotrichales**	0	0	0	0	0	0	0
**Cytophagales**	0	0	1	0	0	0	1
**Rhodospirillales**	0	0	1	0	0	1	1
**Bifidobacteriales**	0	0	0	0	0	1	1
**Bacillales**	1	0	1	0	0	0	0
**Burkholderiales**	0	0	0	1	0	0	0
**Verrucomicrobiales**	0	0	0	0	0	0	0
**Bacteroidales**	0	0	0	0	0	0	0
**Sphaerobacterales**	0	0	0	0	0	0	0
**Spirochaetales**	0	0	0	0	1	0	0
**Cardiobacteriales**	0	0	0	0	0	0	0
**Rubrobacterales**	0	0	0	0	0	0	0
**Opitutales**	0	0	1	0	0	1	1
**Caulobacterales**	0	0	0	0	0	1	1
**Neisseriales**	0	0	0	0	0	0	0
**Methanobacteriales**	0	0	0	0	0	0	0
**Methanomicrobiales**	0	0	0	0	0	0	0
**Flavobacteriales**	0	0	0	0	0	0	0
**Fibrobacterales**	0	0	0	0	0	0	1
**Entomoplasmatales**	0	0	0	0	0	0	0
**Enterobacteriales**	0	0	0	0	0	0	0
**Desulfovibrionales**	0	0	0	0	0	0	1
**Desulfobacterales**	0	0	1	0	0	1	0
**Chromatiales**	0	0	0	0	0	0	0
**Coriobacteriales**	0	0	0	0	0	0	0
**Fusobacteriales**	0	0	0	0	0	0	0
**Bacteroidetes**	0	0	0	0	0	0	0
**Chlorobiales**	0	0	0	0	0	0	0
**Asterales**	0	0	0	0	0	0	0
**Acholeplasmatales**	0	0	0	1	0	0	1

Order	Chromatiales	Coriobacteriales	Fusobacteriales	Bacteroidetes	Chlorobiales	Asterales	Acholeplasmatales
**Clostridiales**	0	1	1	1	1	0	0
**Selenomonadales**	0	0	0	0	0	0	0
**Actinomycetales**	0	0	0	0	0	0	0
**Thermoanaerobacterales**	1	0	0	0	0	0	0
**Synergistales**	0	0	0	0	0	0	0

**Erysipelotrichales**	0	0	0	0	0	0	0
**Cytophagales**	1	0	0	0	0	0	0
**Rhodospirillales**	0	0	0	0	0	0	0
**Bifidobacteriales**	1	0	0	0	0	0	0
**Bacillales**	1	0	0	0	0	0	0
**Burkholderiales**	0	0	0	0	0	0	0
**Verrucomicrobiales**	0	0	0	0	0	0	0
**Bacteroidales**	0	1	0	0	0	1	0
**Sphaerobacterales**	0	0	0	0	0	0	0
**Spirochaetales**	0	0	0	0	0	0	0
**Cardiobacteriales**	0	0	0	0	0	0	0
**Rubrobacterales**	0	0	0	0	0	0	0
**Opitutales**	0	0	0	0	0	0	0
**Caulobacterales**	0	0	0	1	1	0	0
**Neisseriales**	0	0	0	0	0	0	0
**Methanobacteriales**	0	0	0	0	0	0	0
**Methanomicrobiales**	0	0	0	0	0	0	0
**Flavobacteriales**	0	0	0	0	0	0	0
**Fibrobacterales**	0	0	0	0	0	0	0
**Entomoplasmatales**	0	0	0	0	0	0	1
**Enterobacteriales**	0	0	0	0	0	0	0
**Desulfovibrionales**	0	0	0	0	0	0	0
**Desulfobacterales**	0	0	0	0	0	0	1
**Chromatiales**	0	0	0	0	0	0	0
**Coriobacteriales**	0	1	0	0	0	0	0
**Fusobacteriales**	0	0	0	0	0	0	0
**Bacteroidetes**	0	0	0	0	1	0	0
**Chlorobiales**	0	0	0	1	0	0	0
**Asterales**	0	0	0	0	0	0	0
**Acholeplasmatales**	0	0	0	0	0	0	0

Mutual exclusion network

Order	Thermoanaerobacterales	Actinomycetales	Synergistales	Bacteroidales	Spirochaetales	Fibrobacterales	Selenomonadales
**Thermoanaerobacterales**	0	1	0	0	0	0	0
**Actinomycetales**	1	0	0	1	0	0	0
**Synergistales**	0	0	0	1	0	0	0
**Bacteroidales**	0	1	1	0	0	0	1

**Spirochaetales**	0	0	0	0	0	1	0
**Fibrobacterales**	0	0	0	0	1	0	0
**Selenomonadales**	0	0	0	1	0	0	0
**Erysipelotrichales**	0	0	0	0	0	0	1
**Opitutales**	0	0	0	1	0	0	0
**Neisseriales**	0	0	0	0	0	0	0
**Clostridiales**	0	0	0	3	0	1	2
**Lactobacillales**	0	0	1	0	0	0	0
**Coriobacteriales**	0	0	0	0	0	0	0
**Flavobacteriales**	0	0	0	0	0	0	0
**Fabales**	0	0	0	0	0	0	0
**Aeromonadales**	0	0	0	0	0	0	0
**Desulfovibrionales**	0	1	0	1	0	0	0
**Burkholderiales**	0	0	0	0	0	0	0
**Cardiobacteriales**	0	0	0	0	0	1	0
**Bifidobacteriales**	0	0	0	1	0	0	0
**Rhodospirillales**	0	0	0	1	0	0	0
**Caulobacterales**	0	0	0	1	0	0	0
**Bacillales**	0	0	0	0	0	0	0
**Desulfobacterales**	0	0	0	0	0	0	0

**Order**	**Erysipelotrichales**	**Opitutales**	**Neisseriales**	**Clostridiales**	**Lactobacillales**	**Coriobacteriales**	**Flavobacteriales**
**Thermoanaerobacterales**	0	0	0	0	0	0	0
**Actinomycetales**	0	0	0	0	0	0	0
**Synergistales**	0	0	0	0	1	0	0
**Bacteroidales**	0	1	0	3	0	0	0
**Spirochaetales**	0	0	0	0	0	0	0
**Fibrobacterales**	0	0	0	1	0	0	0
**Selenomonadales**	1	0	0	2	0	0	0
**Erysipelotrichales**	1	0	0	1	0	3	1
**Opitutales**	0	0	0	1	0	0	0
**Neisseriales**	0	0	0	1	0	0	0
**Clostridiales**	1	1	1	4	3	0	0
**Lactobacillales**	0	0	0	3	0	1	0
**Coriobacteriales**	3	0	0	0	1	0	0
**Flavobacteriales**	1	0	0	0	0	0	0
**Fabales**	0	0	0	1	0	0	0
**Aeromonadales**	0	0	0	0	0	0	0
**Desulfovibrionales**	0	0	0	1	1	0	0

**Burkholderiales**	0	0	0	1	0	1	0
**Cardiobacteriales**	0	0	0	0	0	0	0
**Bifidobacteriales**	0	0	0	1	0	0	0
**Rhodospirillales**	0	0	0	0	0	0	0
**Caulobacterales**	0	0	0	0	2	0	0
**Bacillales**	0	0	0	0	1	0	0
**Desulfobacterales**	0	0	0	2	0	0	0

**Order**	**Fabales**	**Aeromonadales**	**Desulfovibrionales**	**Burkholderiales**	**Cardiobacteriales**	**Bifidobacteriales**	**Rhodospirillales**
**Thermoanaerobacterales**	0	0	0	0	0	0	0
**Actinomycetales**	0	0	1	0	0	0	0
**Synergistales**	0	0	0	0	0	0	0
**Bacteroidales**	0	0	1	0	0	1	1
**Spirochaetales**	0	0	0	0	0	0	0
**Fibrobacterales**	0	0	0	0	1	0	0
**Selenomonadales**	0	0	0	0	0	0	0
**Erysipelotrichales**	0	0	0	0	0	0	0
**Opitutales**	0	0	0	0	0	0	0
**Neisseriales**	0	0	0	0	0	0	0
**Clostridiales**	1	0	1	1	0	1	0
**Lactobacillales**	0	0	1	0	0	0	0
**Coriobacteriales**	0	0	0	1	0	0	0
**Flavobacteriales**	0	0	0	0	0	0	0
**Fabales**	0	1	0	0	0	0	0
**Aeromonadales**	1	0	0	0	0	0	0
**Desulfovibrionales**	0	0	0	0	0	0	0
**Burkholderiales**	0	0	0	0	0	0	0
**Cardiobacteriales**	0	0	0	0	0	0	0
**Bifidobacteriales**	0	0	0	0	0	0	0
**Rhodospirillales**	0	0	0	0	0	0	0
**Caulobacterales**	0	0	0	0	0	0	0
**Bacillales**	0	0	0	0	0	0	0
**Desulfobacterales**	0	0	0	0	0	0	0

**Order**	**Caulobacterales**	**Bacillales**	**Desulfobacterales**
**Thermoanaerobacterales**	0	0	0
**Actinomycetales**	0	0	0
**Synergistales**	0	0	0
**Bacteroidales**	1	0	0

**Spirochaetales**	0	0	0
**Fibrobacterales**	0	0	0
**Selenomonadales**	0	0	0
**Erysipelotrichales**	0	0	0
**Opitutales**	0	0	0
**Neisseriales**	0	0	0
**Clostridiales**	0	0	2
**Lactobacillales**	2	1	0
**Coriobacteriales**	0	0	0
**Flavobacteriales**	0	0	0
**Fabales**	0	0	0
**Aeromonadales**	0	0	0
**Desulfovibrionales**	0	0	0
**Burkholderiales**	0	0	0
**Cardiobacteriales**	0	0	0
**Bifidobacteriales**	0	0	0
**Rhodospirillales**	0	0	0
**Caulobacterales**	0	0	0
**Bacillales**	0	0	0
**Desulfobacterales**	0	0	0

Co-occurrence network

Order	Clostridiales	Erysipelotrichales	Enterobacteriales	Lactobacillales	Bacteroidales	Vibrionales	Aeromonadales
Clostridiales	62	19	0	5	6	3	1
Erysipelotrichales	19	1	0	0	2	2	1
Enterobacteriales	0	0	6	2	0	0	0
Lactobacillales	5	0	2	0	0	0	0
Bacteroidales	6	2	0	0	2	0	0
Vibrionales	3	2	0	0	0	1	4
Aeromonadales	1	1	0	0	0	4	1
Fibrobacterales	4	1	0	0	2	0	0
Selenomonadales	7	1	3	0	3	0	0
Fusobacteriales	5	1	0	0	0	2	1
Flavobacteriales	5	0	0	0	2	0	0
Coriobacteriales	6	4	0	0	3	0	0
Synergistales	4	1	0	0	1	0	0
Asterales	1	1	0	0	1	0	0
Spirochaetales	4	0	0	0	0	2	1
Desulfovibrionales	1	1	0	0	1	0	0
Bifidobacteriales	3	0	0	0	0	0	0
Burkholderiales	0	0	1	0	2	0	0
Pasteurellales	0	0	0	0	0	1	1
Campylobacterales	0	0	0	1	0	0	0

Order	Fibrobacterales	Selenomonadales	Fusobacteriales	Flavobacteriales	Coriobacteriales	Synergistales	Asterales
Clostridiales	4	7	5	5	6	4	1
Erysipelotrichales	1	1	1	0	4	1	1
Enterobacteriales	0	3	0	0	0	0	0
Lactobacillales	0	0	0	0	0	0	0
Bacteroidales	2	3	0	2	3	1	1
Vibrionales	0	0	2	0	0	0	0
Aeromonadales	0	0	1	0	0	0	0
Fibrobacterales	0	1	0	0	2	1	0
Selenomonadales	1	3	0	2	2	1	3
Fusobacteriales	0	0	0	0	0	0	0
Flavobacteriales	0	2	0	0	0	0	0
Coriobacteriales	2	2	0	0	1	2	0
Synergistales	1	1	0	0	2	0	0
Asterales	0	3	0	0	0	0	0

**Spirochaetales**	0	0	1	0	1	0	0
**Desulfovibrionales**	1	0	0	0	2	0	0
**Bifidobacteriales**	0	0	0	0	0	0	0
**Burkholderiales**	1	0	0	0	0	0	0
**Pasteurellales**	0	0	0	0	0	0	0
**Campylobacterales**	0	0	0	0	0	0	0

Order	Spirochaetales	Desulfovibrionales	Bifidobacteriales	Burkholderiales	Pasteurellales	Campylobacterales
**Clostridiales**	4	1	3	0	0	0
**Erysipelotrichales**	0	1	0	0	0	0
**Enterobacteriales**	0	0	0	1	0	0
**Lactobacillales**	0	0	0	0	0	1
**Bacteroidales**	0	1	0	2	0	0
**Vibrionales**	2	0	0	0	1	0
**Aeromonadales**	1	0	0	0	1	0
**Fibrobacterales**	0	1	0	1	0	0
**Selenomonadales**	0	0	0	0	0	0
**Fusobacteriales**	1	0	0	0	0	0
**Flavobacteriales**	0	0	0	0	0	0
**Coriobacteriales**	1	2	0	0	0	0
**Synergistales**	0	0	0	0	0	0
**Asterales**	0	0	0	0	0	0
**Spirochaetales**	0	0	0	0	0	0
**Desulfovibrionales**	0	0	1	0	0	0
**Bifidobacteriales**	0	1	0	0	0	0
**Burkholderiales**	0	0	0	0	0	0
**Pasteurellales**	0	0	0	0	0	0
**Campylobacterales**	0	0	0	0	0	1

Mutual exclusion network

Order	Coriobacteriales	Bacteroidales	Clostridiales	Lactobacillales	Bifidobacteriales
**Coriobacteriales**	0	1	0	0	0
**Bacteroidales**	1	0	1	0	1
**Clostridiales**	0	1	0	1	0
**Lactobacillales**	0	0	1	0	0
**Bifidobacteriales**	0	1	0	0	0

Co-occurrence network

Order	Enterobacteriales	Actinomycetales	Myxococcales	Clostridiales	Synergistales	Flavobacteriales	Hexacorallia
Enterobacteriales	4	0	0	0	0	2	0
Actinomycetales	0	3	7	2	3	3	3
Myxococcales	0	7	1	0	2	2	2
Clostridiales	0	2	0	3	3	0	0
Synergistales	0	3	2	3	0	1	1
Flavobacteriales	2	3	2	0	1	0	1
Hexacorallia	0	3	2	0	1	1	0
Methanomicrobiales	2	0	0	0	0	1	0
Fusobacteriales	2	0	0	0	0	1	0
Thermoanaerobacterales	0	1	0	1	0	0	0
Opitutales	0	0	0	0	0	0	0
Methylococcales	0	0	0	0	0	0	0
Caulobacterales	0	1	0	1	0	0	0
Sphaerobacterales	0	0	0	1	0	0	0
Rubrobacterales	0	0	0	1	0	0	0
Rhodocyclales	0	0	0	1	0	0	0
Bacillales	0	1	1	0	0	0	0
Fabales	0	0	0	0	0	0	0
Lactobacillales	0	0	0	0	0	0	0
Desulfuromonadales	0	1	0	0	0	0	0
Spirochaetales	0	0	0	0	0	0	0
Chromatiales	0	2	0	0	0	0	0
Deinococcales	0	0	0	0	0	0	0
Sphingomonadales	0	0	0	0	0	0	0
Erysipelotrichales	0	0	0	1	0	0	0
Rhodospirillales	0	1	0	0	0	0	0
Chlorobiales	0	0	0	0	0	0	0
Burkholderiales	0	1	0	0	0	0	0
Asterales	0	1	0	0	0	0	0
Bacteroidales	0	0	0	1	0	0	0

Order	Methanomicrobiales	Fusobacteriales	Thermoanaerobacterales	Opitutales	Methylococcales	Caulobacterales	Sphaerobacterales
Enterobacteriales	2	2	0	0	0	0	0
Actinomycetales	0	0	1	0	0	1	0
Myxococcales	0	0	0	0	0	0	0
Clostridiales	0	0	1	0	0	1	1

**Synergistales**	0	0	0	0	0	0	0
**Flavobacteriales**	1	1	0	0	0	0	0
**Hexacorallia**	0	0	0	0	0	0	0
**Methanomicrobiales**	0	1	0	0	0	0	0
**Fusobacteriales**	1	0	0	0	0	0	0
**Thermoanaerobacterales**	0	0	0	1	1	1	0
**Opitutales**	0	0	1	0	1	0	0
**Methylococcales**	0	0	1	1	0	0	0
**Caulobacterales**	0	0	1	0	0	0	0
**Sphaerobacterales**	0	0	0	0	0	0	0
**Rubrobacterales**	0	0	0	0	0	0	1
**Rhodocyclales**	0	0	0	0	0	0	0
**Bacillales**	0	0	0	0	0	0	0
**Fabales**	0	0	0	0	0	0	0
**Lactobacillales**	0	0	0	0	0	0	0
**Desulfuromonadales**	0	0	0	0	0	0	0
**Spirochaetales**	0	0	0	0	0	0	0
**Chromatiales**	0	0	0	0	0	0	0
**Deinococcales**	0	0	0	0	0	0	0
**Sphingomonadales**	0	0	0	0	0	0	0
**Erysipelotrichales**	0	0	0	0	0	0	0
**Rhodospirillales**	0	0	0	0	0	0	0
**Chlorobiales**	0	0	0	0	0	0	0
**Burkholderiales**	0	0	0	0	0	0	0
**Asterales**	0	0	0	0	0	0	0
**Bacteroidales**	0	0	0	0	0	0	1

**Order**	**Rubrobacterales**	**Rhodocyclales**	**Bacillales**	**Fabales**	**Lactobacillales**	**Desulfuromonadales**	**Spirochaetales**
**Enterobacteriales**	0	0	0	0	0	0	0
**Actinomycetales**	0	0	1	0	0	1	0
**Myxococcales**	0	0	1	0	0	0	0
**Clostridiales**	1	1	0	0	0	0	0
**Synergistales**	0	0	0	0	0	0	0
**Flavobacteriales**	0	0	0	0	0	0	0
**Hexacorallia**	0	0	0	0	0	0	0
**Methanomicrobiales**	0	0	0	0	0	0	0
**Fusobacteriales**	0	0	0	0	0	0	0
**Thermoanaerobacterales**	0	0	0	0	0	0	0
**Opitutales**	0	0	0	0	0	0	0

**Methylococcales**	0	0	0	0	0	0	0
**Caulobacterales**	0	0	0	0	0	0	0
**Sphaerobacterales**	1	0	0	0	0	0	0
**Rubrobacterales**	0	0	0	0	0	0	0
**Rhodocyclales**	0	0	0	0	0	0	0
**Bacillales**	0	0	0	0	0	0	0
**Fabales**	0	0	0	0	1	0	0
**Lactobacillales**	0	0	0	1	0	0	0
**Desulfuromonadales**	0	0	0	0	0	0	1
**Spirochaetales**	0	0	0	0	0	1	0
**Chromatiales**	0	0	0	0	0	1	0
**Deinococcales**	0	0	0	0	0	0	0
**Sphingomonadales**	0	0	0	0	0	0	0
**Erysipelotrichales**	0	0	0	0	0	0	0
**Rhodospirillales**	0	0	0	0	0	0	0
**Chlorobiales**	0	0	0	0	0	0	0
**Burkholderiales**	0	0	0	0	0	1	0
**Asterales**	0	0	0	0	0	1	0
**Bacteroidales**	1	0	0	0	0	0	0

Order	Chromatiales	Deinococcales	Sphingomonadales	Erysipelotrichales	Rhodospirillales	Chlorobiales	Burkholderiales
**Enterobacteriales**	0	0	0	0	0	0	0
**Actinomycetales**	2	0	0	0	1	0	1
**Myxococcales**	0	0	0	0	0	0	0
**Clostridiales**	0	0	0	1	0	0	0
**Synergistales**	0	0	0	0	0	0	0
**Flavobacteriales**	0	0	0	0	0	0	0
**Hexacorallia**	0	0	0	0	0	0	0
**Methanomicrobiales**	0	0	0	0	0	0	0
**Fusobacteriales**	0	0	0	0	0	0	0
**Thermoanaerobacterales**	0	0	0	0	0	0	0
**Opitutales**	0	0	0	0	0	0	0
**Methylococcales**	0	0	0	0	0	0	0
**Caulobacterales**	0	0	0	0	0	0	0
**Sphaerobacterales**	0	0	0	0	0	0	0
**Rubrobacterales**	0	0	0	0	0	0	0
**Rhodocyclales**	0	0	0	0	0	0	0
**Bacillales**	0	0	0	0	0	0	0
**Fabales**	0	0	0	0	0	0	0

**Lactobacillales**	0	0	0	0	0	0	0
**Desulfuromonadales**	1	0	0	0	0	0	1
**Spirochaetales**	0	0	0	0	0	0	0
**Chromatiales**	0	0	0	0	1	0	1
**Deinococcales**	0	0	1	0	0	0	0
**Sphingomonadales**	0	1	0	0	0	0	0
**Erysipelotrichales**	0	0	0	0	0	1	0
**Rhodospirillales**	1	0	0	0	0	0	0
**Chlorobiales**	0	0	0	1	0	0	0
**Burkholderiales**	1	0	0	0	0	0	0
**Asterales**	1	0	0	0	0	0	1
**Bacteroidales**	0	0	0	0	0	0	0

**Order**	**Asterales**	**Bacteroidales**
**Enterobacteriales**	0	0
**Actinomycetales**	1	0
**Myxococcales**	0	0
**Clostridiales**	0	1
**Synergistales**	0	0
**Flavobacteriales**	0	0
**Hexacorallia**	0	0
**Methanomicrobiales**	0	0
**Fusobacteriales**	0	0
**Thermoanaerobacterales**	0	0
**Opitutales**	0	0
**Methylococcales**	0	0
**Caulobacterales**	0	0
**Sphaerobacterales**	0	1
**Rubrobacterales**	0	1
**Rhodocyclales**	0	0
**Bacillales**	0	0
**Fabales**	0	0
**Lactobacillales**	0	0
**Desulfuromonadales**	1	0
**Spirochaetales**	0	0
**Chromatiales**	1	0
**Deinococcales**	0	0
**Sphingomonadales**	0	0
**Erysipelotrichales**	0	0

**Rhodospirillales**	0	0
**Chlorobiales**	0	0
**Burkholderiales**	1	0
**Asterales**	0	0
**Bacteroidales**	0	0

Mutual exclusion network

Order	Verrucomicrobiales	Pasteurellales	Thermoanaerobacterales	Clostridiales	Synergistales	Fusobacteriales	Erysipelotrichales
**Verrucomicrobiales**	0	1	0	0	0	0	0
**Pasteurellales**	1	0	0	0	0	0	0
**Thermoanaerobacterales**	0	0	0	1	0	0	0
**Clostridiales**	0	0	1	1	1	0	0
**Synergistales**	0	0	0	1	0	0	0
**Fusobacteriales**	0	0	0	0	0	0	1
**Erysipelotrichales**	0	0	0	0	0	1	0
**Desulfovibrionales**	0	0	0	0	0	0	0
**Hydrogenophilales**	0	0	0	0	0	0	0
**Coriobacteriales**	0	0	0	0	0	0	0
**Enterobacteriales**	0	0	0	1	0	0	0
**Caulobacterales**	0	0	0	1	0	0	0
**Actinomycetales**	0	0	0	1	0	0	0
**Burkholderiales**	0	0	0	0	0	0	0
**Selenomonadales**	0	0	0	0	0	0	0
**Bacteroidales**	0	0	0	0	0	0	0
**Lactobacillales**	0	0	0	0	0	0	0
**Fabales**	0	0	0	0	0	0	0

Order	Desulfovibrionales	Hydrogenophilales	Coriobacteriales	Enterobacteriales	Caulobacterales	Actinomycetales	Burkholderiales
**Verrucomicrobiales**	0	0	0	0	0	0	0
**Pasteurellales**	0	0	0	0	0	0	0
**Thermoanaerobacterales**	0	0	0	0	0	0	0
**Clostridiales**	0	0	0	1	1	1	0
**Synergistales**	0	0	0	0	0	0	0
**Fusobacteriales**	0	0	0	0	0	0	0
**Erysipelotrichales**	0	0	0	0	0	0	0
**Desulfovibrionales**	0	1	0	0	0	0	0
**Hydrogenophilales**	1	0	0	0	0	0	0

**Coriobacteriales**	0	0	1	0	0	0	0
**Enterobacteriales**	0	0	0	0	0	0	0
**Caulobacterales**	0	0	0	0	0	0	0
**Actinomycetales**	0	0	0	0	0	0	0
**Burkholderiales**	0	0	0	0	0	0	0
**Selenomonadales**	0	0	0	0	0	0	1
**Bacteroidales**	0	0	0	0	0	0	0
**Lactobacillales**	0	0	0	0	0	0	0
**Fabales**	0	0	0	0	0	0	0

Order	Selenomonadales	Bacteroidales	Lactobacillales	Fabales
**Verrucomicrobiales**	0	0	0	0
**Pasteurellales**	0	0	0	0
**Thermoanaerobacterales**	0	0	0	0
**Clostridiales**	0	0	0	0
**Synergistales**	0	0	0	0
**Fusobacteriales**	0	0	0	0
**Erysipelotrichales**	0	0	0	0
**Desulfovibrionales**	0	0	0	0
**Hydrogenophilales**	0	0	0	0
**Coriobacteriales**	0	0	0	0
**Enterobacteriales**	0	0	0	0
**Caulobacterales**	0	0	0	0
**Actinomycetales**	0	0	0	0
**Burkholderiales**	1	0	0	0
**Selenomonadales**	0	0	0	0
**Bacteroidales**	0	0	1	1
**Lactobacillales**	0	1	0	0
**Fabales**	0	1	0	0

Co-occurrence network

Order	Clostridiales	Bacteroidales	Coriobacteriales	Selenomonadales	Erysipelotrichales	Synergistales	Bacillales
**Clostridiales**	69	30	21	18	24	16	13
**Bacteroidales**	30	8	2	1	3	4	4
**Coriobacteriales**	21	2	2	2	4	5	3
**Selenomonadales**	18	1	2	4	4	3	2
**Erysipelotrichales**	24	3	4	4	2	3	4
**Synergistales**	16	4	5	3	3	1	2
**Bacillales**	13	4	3	2	4	2	0
**Spirochaetales**	9	0	1	2	1	1	1
**Asterales**	12	2	2	1	2	1	1
**Fibrobacterales**	9	2	3	2	2	2	2
**Desulfovibrionales**	8	7	2	4	2	2	3
**Chromatiales**	5	1	3	2	1	2	1
**Burkholderiales**	6	6	2	0	0	2	1
**Aeromonadales**	5	0	1	0	0	0	0
**Thermoanaerobacterales**	7	1	2	2	3	2	1
**Pasteurellales**	5	3	2	1	1	2	1
**Hydrogenophilales**	2	4	0	0	1	0	1
**Lactobacillales**	3	0	0	0	1	0	0
**Flavobacteriales**	3	2	1	1	1	0	1
**Enterobacteriales**	0	0	0	4	0	0	0
**Verrucomicrobiales**	0	1	0	0	0	0	0
**Neisseriales**	0	0	0	0	0	0	0
**Bifidobacteriales**	2	0	0	0	0	0	1
**Actinomycetales**	0	0	0	0	0	0	0
**Fabales**	0	0	0	0	0	0	0

Order	Spirochaetales	Asterales	Fibrobacterales	Desulfovibrionales	Chromatiales	Burkholderiales	Aeromonadales
**Clostridiales**	9	12	9	8	5	6	5
**Bacteroidales**	0	2	2	7	1	6	0
**Coriobacteriales**	1	2	3	2	3	2	1
**Selenomonadales**	2	1	2	4	2	0	0
**Erysipelotrichales**	1	2	2	2	1	0	0
**Synergistales**	1	1	2	2	2	2	0
**Bacillales**	1	1	2	3	1	1	0
**Spirochaetales**	0	1	0	1	0	0	1
**Asterales**	1	0	1	0	0	0	0

**Fibrobacterales**	0	1	0	1	1	1	0
**Desulfovibrionales**	1	0	1	1	2	3	0
**Chromatiales**	0	0	1	2	0	1	0
**Burkholderiales**	0	0	1	3	1	0	1
**Aeromonadales**	1	0	0	0	0	1	0
**Thermoanaerobacterales**	0	0	1	1	1	1	1
**Pasteurellales**	0	0	1	2	1	1	0
**Hydrogenophilales**	0	0	0	0	0	0	0
**Lactobacillales**	0	0	0	0	0	0	0
**Flavobacteriales**	0	0	1	2	0	0	0
**Enterobacteriales**	0	0	0	0	0	2	0
**Verrucomicrobiales**	0	0	0	1	0	0	0
**Neisseriales**	0	0	0	0	0	1	0
**Bifidobacteriales**	0	0	0	0	0	0	0
**Actinomycetales**	0	0	0	0	0	0	0
**Fabales**	0	0	0	0	0	0	0

Order	Thermoanaerobacterales	Pasteurellales	Hydrogenophilales	Lactobacillales	Flavobacteriales	Enterobacteriales	Verrucomicrobiales
**Clostridiales**	7	5	2	3	3	0	0
**Bacteroidales**	1	3	4	0	2	0	1
**Coriobacteriales**	2	2	0	0	1	0	0
**Selenomonadales**	2	1	0	0	1	4	0
**Erysipelotrichales**	3	1	1	1	1	0	0
**Synergistales**	2	2	0	0	0	0	0
**Bacillales**	1	1	1	0	1	0	0
**Spirochaetales**	0	0	0	0	0	0	0
**Asterales**	0	0	0	0	0	0	0
**Fibrobacterales**	1	1	0	0	1	0	0
**Desulfovibrionales**	1	2	0	0	2	0	1
**Chromatiales**	1	1	0	0	0	0	0
**Burkholderiales**	1	1	0	0	0	2	0
**Aeromonadales**	1	0	0	0	0	0	0
**Thermoanaerobacterales**	0	1	0	0	0	0	0
**Pasteurellales**	1	0	0	0	0	0	1
**Hydrogenophilales**	0	0	0	0	1	0	0
**Lactobacillales**	0	0	0	0	0	0	0
**Flavobacteriales**	0	0	1	0	0	0	0
**Enterobacteriales**	0	0	0	0	0	2	0
**Verrucomicrobiales**	0	1	0	0	0	0	0

**Neisseriales**	0	0	0	0	0	0	0
**Bifidobacteriales**	0	0	0	0	0	0	0
**Actinomycetales**	0	0	0	0	0	0	0
**Fabales**	0	0	0	0	0	0	0

Order	Neisseriales	Bifidobacteriales	Actinomycetales	Fabales
**Clostridiales**	0	2	0	0
**Bacteroidales**	0	0	0	0
**Coriobacteriales**	0	0	0	0
**Selenomonadales**	0	0	0	0
**Erysipelotrichales**	0	0	0	0
**Synergistales**	0	0	0	0
**Bacillales**	0	1	0	0
**Spirochaetales**	0	0	0	0
**Asterales**	0	0	0	0
**Fibrobacterales**	0	0	0	0
**Desulfovibrionales**	0	0	0	0
**Chromatiales**	0	0	0	0
**Burkholderiales**	1	0	0	0
**Aeromonadales**	0	0	0	0
**Thermoanaerobacterales**	0	0	0	0
**Pasteurellales**	0	0	0	0
**Hydrogenophilales**	0	0	0	0
**Lactobacillales**	0	0	0	0
**Flavobacteriales**	0	0	0	0
**Enterobacteriales**	0	0	0	0
**Verrucomicrobiales**	0	0	0	0
**Neisseriales**	0	0	0	0
**Bifidobacteriales**	0	0	0	0
**Actinomycetales**	0	0	0	1
**Fabales**	0	0	1	0

Mutual exclusion network

Order	Xanthomonadales	Burkholderiales	Selenomonadales	Pasteurellales	Actinomycetales	Lactobacillales	Hydrogenophilales
**Xanthomonadales**	0	1	1	0	0	0	0
**Burkholderiales**	1	0	0	0	1	0	0
**Selenomonadales**	1	0	0	0	0	0	0

**Pasteurellales**	0	0	0	0	1	0	0
**Actinomycetales**	0	1	0	1	0	0	0
**Lactobacillales**	0	0	0	0	0	0	1
**Hydrogenophilales**	0	0	0	0	0	1	0
**Clostridiales**	2	0	0	0	2	1	0
**Bacillales**	0	0	0	0	0	1	0
**Flavobacteriales**	0	0	0	0	0	1	0
**Enterobacteriales**	0	1	0	0	0	0	0
**Bifidobacteriales**	0	0	0	0	0	0	0
**Bacteroidales**	2	0	0	0	3	4	0

**Order**	**Clostridiales**	**Bacillales**	**Flavobacteriales**	**Enterobacteriales**	**Bifidobacteriales**	**Bacteroidales**
**Xanthomonadales**	2	0	0	0	0	2
**Burkholderiales**	0	0	0	1	0	0
**Selenomonadales**	0	0	0	0	0	0
**Pasteurellales**	0	0	0	0	0	0
**Actinomycetales**	2	0	0	0	0	3
**Lactobacillales**	1	1	1	0	0	4
**Hydrogenophilales**	0	0	0	0	0	0
**Clostridiales**	1	0	0	0	1	0
**Bacillales**	0	0	0	0	0	0
**Flavobacteriales**	0	0	0	0	0	0
**Enterobacteriales**	0	0	0	0	0	0
**Bifidobacteriales**	1	0	0	0	0	0
**Bacteroidales**	0	0	0	0	0	0

Co-occurrence network

Order	Clostridiales	Fusobacteriales	Coriobacteriales	Thermoanaerobacterales	Bacillales	Synergistales	Bacteroidales
**Clostridiales**	23	11	13	11	8	7	0
**Fusobacteriales**	11	1	0	6	2	0	0
**Coriobacteriales**	13	0	5	0	0	2	0
**Thermoanaerobacterales**	11	6	0	1	1	1	0
**Bacillales**	8	2	0	1	0	1	0
**Synergistales**	7	0	2	1	1	1	0
**Bacteroidales**	0	0	0	0	0	0	5
**Rhodospirillales**	2	0	0	1	1	4	1
**Selenomonadales**	6	0	3	0	0	1	0
**Erysipelotrichales**	5	3	0	2	2	0	0
**Asterales**	3	0	2	0	0	1	0
**Verrucomicrobiales**	0	0	0	0	0	0	0
**Lactobacillales**	2	1	0	1	0	0	0
**Flavobacteriales**	0	0	0	0	0	0	2
**Burkholderiales**	1	0	0	1	0	2	0
**Spirochaetales**	0	0	0	0	0	1	0
**Entomoplasmatales**	1	0	0	1	0	0	0
**Desulfovibrionales**	0	0	0	0	0	0	0
**Actinomycetales**	0	0	1	0	0	0	0
**Bifidobacteriales**	0	0	1	0	0	0	0
**Acholeplasmatales**	1	0	0	1	0	0	0

Order	Rhodospirillales	Selenomonadales	Erysipelotrichales	Asterales	Verrucomicrobiales	Lactobacillales	Flavobacteriales
**Clostridiales**	2	6	5	3	0	2	0
**Fusobacteriales**	0	0	3	0	0	1	0
**Coriobacteriales**	0	3	0	2	0	0	0
**Thermoanaerobacterales**	1	0	2	0	0	1	0
**Bacillales**	1	0	2	0	0	0	0
**Synergistales**	4	1	0	1	0	0	0
**Bacteroidales**	1	0	0	0	0	0	2
**Rhodospirillales**	3	0	0	0	2	0	0
**Selenomonadales**	0	1	1	1	0	0	0
**Erysipelotrichales**	0	1	0	0	0	1	0
**Asterales**	0	1	0	0	0	0	0
**Verrucomicrobiales**	2	0	0	0	0	0	0
**Lactobacillales**	0	0	1	0	0	0	0

**Flavobacteriales**	0	0	0	0	0	0	0
**Burkholderiales**	1	0	0	0	0	0	0
**Spirochaetales**	0	0	0	0	0	0	0
**Entomoplasmatales**	0	0	1	0	0	0	0
**Desulfovibrionales**	1	0	0	0	0	0	0
**Actinomycetales**	0	0	0	0	0	0	0
**Bifidobacteriales**	0	0	0	0	0	0	0
**Acholeplasmatales**	0	0	0	0	0	0	0

Order	Burkholderiales	Spirochaetales	Entomoplasmatales	Desulfovibrionales	Actinomycetales	Bifidobacteriales	Acholeplasmatales
**Clostridiales**	1	0	1	0	0	0	1
**Fusobacteriales**	0	0	0	0	0	0	0
**Coriobacteriales**	0	0	0	0	1	1	0
**Thermoanaerobacterales**	1	0	1	0	0	0	1
**Bacillales**	0	0	0	0	0	0	0
**Synergistales**	2	1	0	0	0	0	0
**Bacteroidales**	0	0	0	0	0	0	0
**Rhodospirillales**	1	0	0	1	0	0	0
**Selenomonadales**	0	0	0	0	0	0	0
**Erysipelotrichales**	0	0	1	0	0	0	0
**Asterales**	0	0	0	0	0	0	0
**Verrucomicrobiales**	0	0	0	0	0	0	0
**Lactobacillales**	0	0	0	0	0	0	0
**Flavobacteriales**	0	0	0	0	0	0	0
**Burkholderiales**	0	1	0	0	0	0	0
**Spirochaetales**	1	0	0	0	0	0	0
**Entomoplasmatales**	0	0	0	0	0	0	1
**Desulfovibrionales**	0	0	0	0	0	0	0
**Actinomycetales**	0	0	0	0	0	0	0
**Bifidobacteriales**	0	0	0	0	0	0	0
**Acholeplasmatales**	0	0	1	0	0	0	0

Mutual exclusion network

Order	Selenomonadales	Anthribidae	Flavobacteriales	Clostridiales	Erysipelotrichales	Bacteroidales	Synergistales
**Selenomonadales**	1	1	0	1	0	2	0
**Anthribidae**	1	0	0	0	0	0	0
**Flavobacteriales**	0	0	0	2	0	0	0

**Clostridiales**	1	0	2	2	0	10	1
**Erysipelotrichales**	0	0	0	0	0	2	0
**Bacteroidales**	2	0	0	10	2	0	0
**Synergistales**	0	0	0	1	0	0	0
**Asterales**	0	0	0	2	0	0	0
**Lactobacillales**	0	0	0	0	0	1	0
**Fusobacteriales**	0	0	0	0	0	1	0
**Coriobacteriales**	0	0	0	2	0	0	0
**Thermoanaerobacterales**	0	0	0	0	0	2	0

**Order**	**Asterales**	**Lactobacillales**	**Fusobacteriales**	**Coriobacteriales**	**Thermoanaerobacterales**
**Selenomonadales**	0	0	0	0	0
**Anthribidae**	0	0	0	0	0
**Flavobacteriales**	0	0	0	0	0
**Clostridiales**	2	0	0	2	0
**Erysipelotrichales**	0	0	0	0	0
**Bacteroidales**	0	1	1	0	2
**Synergistales**	0	0	0	0	0
**Asterales**	0	0	0	0	0
**Lactobacillales**	0	0	0	0	0
**Fusobacteriales**	0	0	0	0	0
**Coriobacteriales**	0	0	0	0	0
**Thermoanaerobacterales**	0	0	0	0	0
