# Supplementary material for: Global investigation of composition and interaction networks in gut microbiomes of individuals belonging to diverse geographies and age-groups
Source: Gut Pathog. 2016 May 6;8:17. doi: 10.1186/s13099-016-0099-z (PMC4858888; doi:10.1186/s13099-016-0099-z)
Supplement: Supplementary file 13 — 10.1186/s13099-016-0099-z List of genus occurring only in older age groups in gut microbial compositions. [file 13099_2016_99_MOESM13_ESM.pdf]

List of genus occurring only in older age groups in gut microbial compositions

1	Saccharopolyspora
2	Methylocella
3	Acidiphilum
4	Oligotropha
5	Alkalilimnicola
6	Nitrobacter
7	Acidobacterium
8	Gluconacetobacter
9	Chloroflexus
10	Clavibacter
11	Nocardioides
12	Thermomonospora
13	Rhodothermus
14	Methylibium
15	Fikenella
16	Marinobacter
17	Scardovia
18	Thermotoga
19	Methylococcus
20	Chromohalobacter
21	Eremococcus
22	Gemmatimonas
23	Borrelia
24	Lawsonia
25	Erysipelothrix
26	Dechloromonas
27	Bartonella
28	Sphingopyxis
29	Mesorhizobium
30	Paracoccus
31	Cytophaga
32	Ruegeria
33	Actinosynnema
34	Aromatoleum
