# Supplementary material for: Global investigation of composition and interaction networks in gut microbiomes of individuals belonging to diverse geographies and age-groups
Source: Gut Pathog. 2016 May 6;8:17. doi: 10.1186/s13099-016-0099-z (PMC4858888; doi:10.1186/s13099-016-0099-z)
Supplement: Supplementary file 14 — 10.1186/s13099-016-0099-z Regression coefficients of the abundance patterns of various genera with first component (PC1) of the PLS regression analysis. [file 13099_2016_99_MOESM14_ESM.pdf]

Regression coefficients of the abundance patterns of various genera with first component (PC1) of the PLS regression analysis

Genera	Regression Coefficient with PC1 components (R^2)
Bulleidia	0.9115
Porphyromonas	0.8780
Bacteroides	0.8752
Finegoldia	0.8541
Paraprevotella	0.8377
Succinatimonas	0.8356
Prevotella	0.8305
Dysgonomonas	0.7968
Faecalibacterium	0.7857
Butyrivibrio	0.7729
Ruminococcus	0.7582
Eggerthella	0.7537
Phascolarctobacterium	0.7510
Roseburia	0.7396
Mitsuokella	0.7327
Alistipes	0.7192
Clostridium	0.7131
Selenomonas	0.7092
Collinsella	0.7080
Blautia	0.6873
Catenibacterium	0.6801
Pseudoflavonifractor	0.6751
Abiotropha	0.6718
Peptoniphilus	0.6681
Fibrobacter	0.6679
Shuttleworthia	0.6557
Slackia	0.6514
Pyramidobacter	0.6484917
Oribacterium	0.6383648
Anaerofustis	0.6373584
Dialister	0.6226295
Turicibacter	0.6213367
Symbiobacterium	0.6207816
Atopobium	0.6205472
Akkermansia	0.6165538
Eubacterium	0.6146568
Heliobacterium	0.60824
Subdoligranulum	0.5925884
Holdemania	0.5870195

Desulfitobacterium	0.5800244
Solobacterium	0.5672282
Sutterella	0.5654783
Coprobacillus	0.5588766
Capnocytophaga	0.5555231
Anaerostipes	0.549974
Acidaminococcus	0.5387127
Bifidobacterium	0.5385378
Coprococcus	0.5316589
Anaerotruncus	0.5031967
Bilophila	0.4909073
Parabacteroides	0.4796097
Veillonella	0.4790428
Dorea	0.4777974
Escherichia	0.4698832
Megasphaera	0.4558009
Peptostreptococcus	0.450941
Pyramidobacter	0.6484917
