# Supplementary material for: Global investigation of composition and interaction networks in gut microbiomes of individuals belonging to diverse geographies and age-groups
Source: Gut Pathog. 2016 May 6;8:17. doi: 10.1186/s13099-016-0099-z (PMC4858888; doi:10.1186/s13099-016-0099-z)
Supplement: Supplementary file 18 — 10.1186/s13099-016-0099-z Nation specific intake of the various food components (computed for 1000 individuals) obtained from the website of the Food and Agricultural Organization of the United Nations and the food diversity indices (Berry Index and Healthy Food Diversity (HFD) index computed for each geographic region. [file 13099_2016_99_MOESM18_ESM.pdf]

Food Components	America	China	Denmark	France	India	Italy	Japan	Spain
Cereals - Excluding Beer	1.0331	0.3290	1.5010	0.5684	0.1765	0.4171	0.2506	0.6429
Starchy Roots	0.0719	0.1363	0.2802	0.0924	0.0419	0.0476	0.0406	0.0807
Sugar Crops	0.1686	0.0899	0.5230	0.5993	0.2804	0.0584	0.0357	0.0901
Sugar & Sweeteners	0.0636	0.0114	0.1159	0.0613	0.0242	0.0314	0.0292	0.0260
Pulses	0.0037	0.0032	0.0054	0.0087	0.0169	0.0068	0.0019	0.0157
Treenuts	0.0026	0.0027	0.0052	0.0041	0.0014	0.0084	0.0017	0.0067
Oilcrops	0.1893	0.0824	0.1117	0.1161	0.0410	0.0963	0.0468	0.2640
Vegetable Oils	0.0429	0.0216	0.0750	0.0494	0.0136	0.0518	0.0215	0.0594
Vegetables	0.1208	0.3947	0.1283	0.1220	0.0858	0.1732	0.1107	0.1550
Fruits - Excluding Wine	0.1155	0.0985	0.1058	0.2353	0.0604	0.2591	0.0577	0.2087
Coffee/Tea	0.0074	0.0011	0.0138	0.0105	0.0007	0.0078	0.0061	0.0075
Meat	0.1180	0.0579	0.0753	0.0900	0.0041	0.0870	0.0497	0.0930
Offals	0.0030	0.0037	0.0056	0.0063	0.0004	0.0026	0.0025	0.0027
Animal fats	0.0186	0.0033	0.0766	0.0146	0.0030	0.0140	0.0045	0.0192
Eggs	0.0167	0.0204	0.0178	0.0140	0.0028	0.0124	0.0199	0.0153
Milk - Excluding Butter	0.2759	0.0336	0.4065	0.2857	0.1049	0.2753	0.0733	0.2263
Fish, Seafood	0.0255	0.0408	0.0626	0.0347	0.0057	0.0294	0.0698	0.0470
Aquatic Products, Other	0.0002	0.0092	0.0016	0.0008	0.0000	0.0005	0.0056	0.0008

**HFD Index**	0.1066	0.1438	0.1019	0.0987	0.0944	0.1277	0.1244	0.1165
**Berry Index**	0.7566	0.8248	0.7692	0.8370	0.8173	0.8498	0.8569	0.8355
