# Supplementary material for: Global investigation of composition and interaction networks in gut microbiomes of individuals belonging to diverse geographies and age-groups
Source: Gut Pathog. 2016 May 6;8:17. doi: 10.1186/s13099-016-0099-z (PMC4858888; doi:10.1186/s13099-016-0099-z)
Supplement: Supplementary file 19 — 10.1186/s13099-016-0099-z Evaluation of possible biases arising due to differences in datasets used in the present study and effects of such biases on the results. [file 13099_2016_99_MOESM19_ESM.pdf]

## Datasets Used

Publicly available 399 gut metagenomes were downloaded as summarized in Table 1 below.

*Table1. Geographic distribution of datasets used*

| Geographic region | Number of datasets | Other Information | Reference number of main manuscript |
|---|---|---|---|
| America | 90 | - | 6 |
| China | 144 | 70 datasets from diabetic subjects | 8 |
| Denmark | 81 | - | 4 |
| France | 8 | - | 7 |
| India | 22 | 14 datasets from malnourished subjects (severely malnourished + borderline malnourishment indices) | 6, 22 |
| Italy | 6 | - | 7 |
| Japan | 13 | - | 7 |
| Spain | 35 | 35 datasets from individuals suffering from either Crohn's disease or Ulcerative colitis (out of which 23 had IBD) | 4 |
| Total | 399 | - | - |

**Evaluation of possible biases arising due to using different sequencing methods in different studies and effects of such biases on the results:**

Previous studies have noted that a major concern with using samples from multiple studies is the presence of study-specific biases [reference 40 of main manuscript]. In the context of metagenomic datasets (in contrast to 16S rDNA-based amplicon studies), these biases primarily originate from differences in DNA extraction protocols and the sequencing platforms used for obtaining the metagenomic data. Such biases result in the abundance profiles of the different samples clustering by study (with inter-study variations being much stronger than inter-individual ones). To evaluate whether there were study specific biases in the datasets used in our analysis, a Principal Component Analysis (PCA) was performed for the abundance profiles of the 8 regions (obtained from the five different studies). It was observed that all the samples clustered together as seen in Figure 1 below.

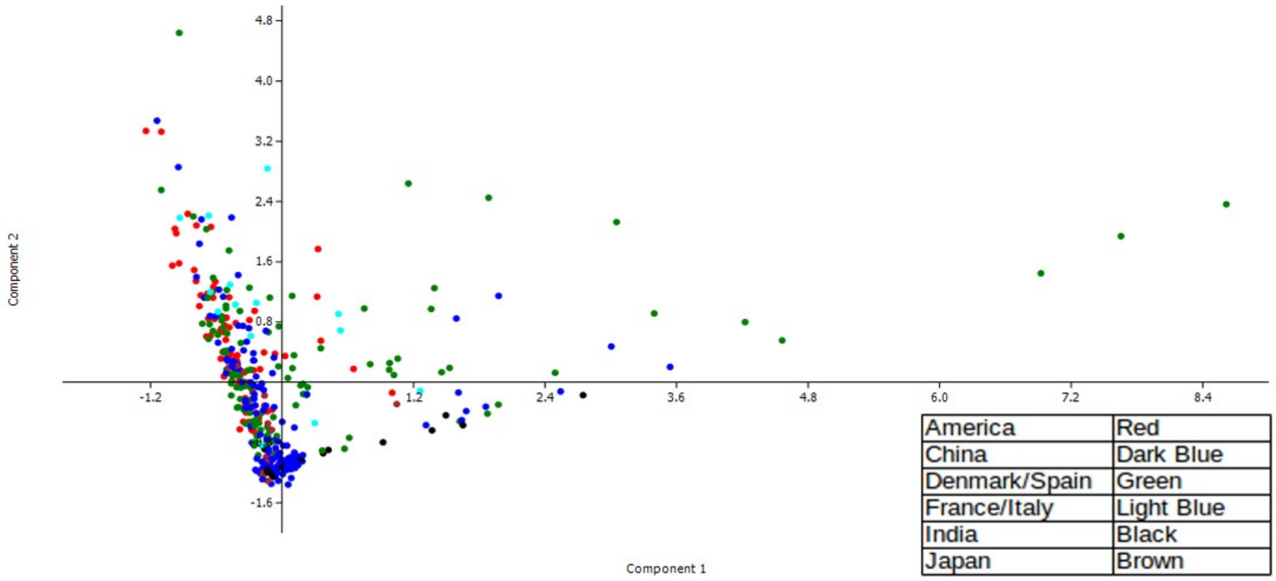


*Figure 1. PCA of the abundance profiles of the 8 regions*

A comparison of the first and second principal components of all 8 regions (Figure 2 A and B) indicated no significant differences in either the PC1 ($P < 0.768$) or PC2 ($P < 0.784$) components. These results indicate the absence of study-specific clustering of the abundance profiles of the different samples.

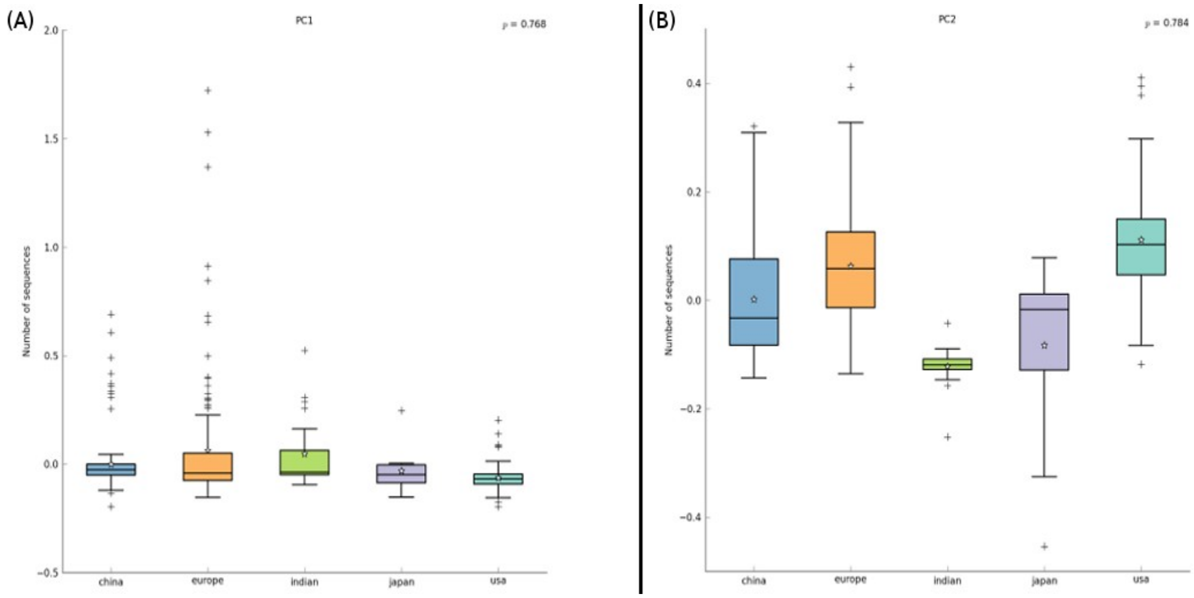


*Figure2. Box plot of PC1 and PC2 components of the 8 regions*

**Evaluation of possible biases arising due to datasets having different average contig lengths and effects of such biases on the results:**

Subsequently, the average contig lengths from all regions were compared. This was to ensure that the findings of the current study were not artefacts of the differences in contig lengths across different

samples. As seen in Figure 3 below, the contigs from Chinese samples were observed to be the longest. On the other hand, contigs of samples from France and Italy were the shortest in length.

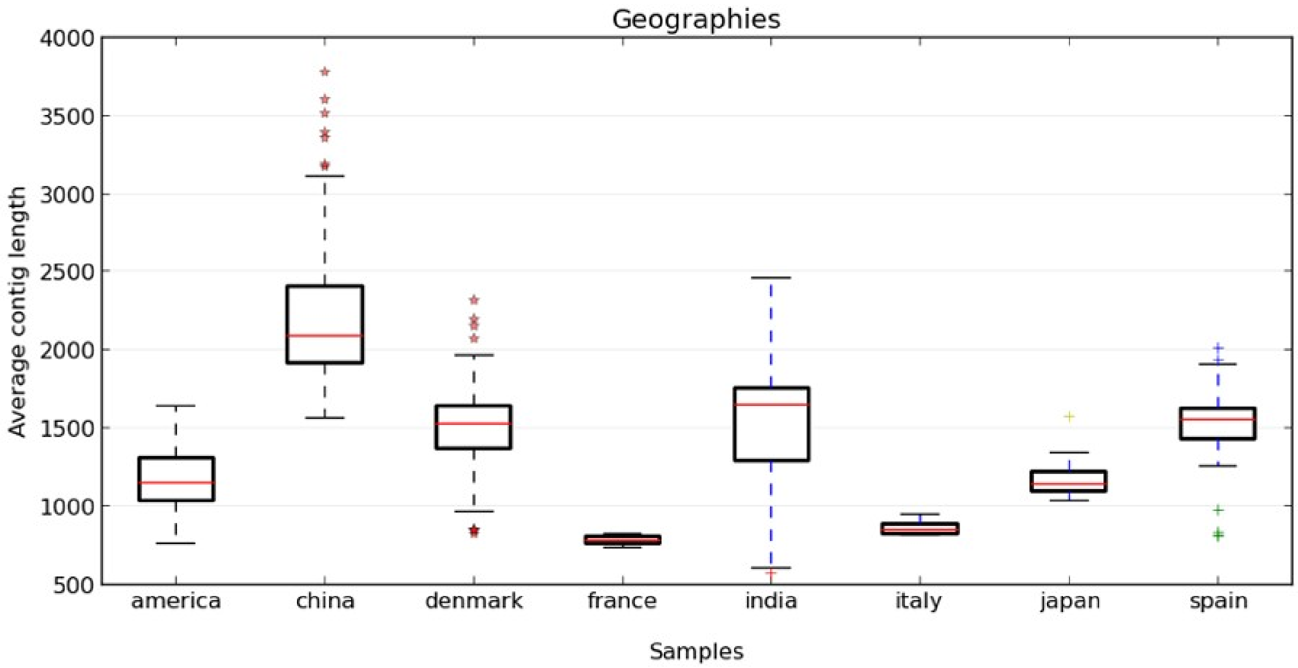


*Figure 3. Box plot of average contig lengths of the 8 regions*

To confirm the accuracy of our results (and verify that differences in contig lengths did not have any influence on the detection sensitivity of the various genera), random sub-strings of contigs were obtained from the different American, Chinese, Danish, Indian, Japanese and Spanish samples, considering the average length of substring to be similar to that of French samples (having the lowest average contig length of around 784 bp). These were referred to as 'Short Sequence' datasets for each sample. The taxonomic assignment of these short sequence datasets was then performed and the genera abundances thus obtained were compared with the original abundance profile of the corresponding sample. Table 2 below gives the average correlation between taxonomic assignments of the various genera from original datasets and the corresponding short sequence datasets.

*Table 2. Average correlation between taxonomic assignments of various genera from original datasets and the corresponding short sequence datasets*

| Geographic region | Average Correlation |
|---|---|
| America | 0.829 |
| China | 0.897 |
| Denmark | 0.884 |
| India | 0.94 |
| Japan | 0.951 |
| Spain | 0.884 |

It can be observed that the average correlation between the abundances of various genera exceeded 0.824 for all the geographies. For five out of the six geographies, the average correlation exceeded 0.88, indicating that even by taking shorter lengths of contigs (similar to the French and Italy samples), the taxonomic abundance pattern obtained (for the other samples) was similar to the original profile. Thus it can be concluded that differences in contig lengths were not likely to contribute to study-specific artefactual biases in the current analysis.

**Evaluation of possible biases arising due to differences in health status of subjects:**

As suggested by a few previous studies [reference 10, 11 in main manuscript], the geography specific differences in the microbiome may be expected to be significantly more prominent than changes related to the health conditions (relevant to the datasets considered in our study). For example, a comparative study by Karlsson and co-workers (reference 36 in main manuscript), performed with gut-microbiome datasets from Sweden and China, has indicated that gut-microbiomes from Swedish individuals having diabetes are more similar in composition to those of healthy Swedish individuals, when compared to Chinese individuals (either diabetic or healthy). We evaluated whether disease condition specific variations in the gut microbiota may confound our understanding of geography specific variations using the following method. Two separate abundance profiles were created for microbiome samples collected from diabetic and non-diabetic individuals from China [reference 8 in main manuscript]. Similarly, three separate abundance profiles were created for Indian datasets, viz., apparently healthy (AH), borderline (BL) and severely malnourished (SM) [reference 22 in main manuscript]. Euclidean distances (based on average abundance profiles) between these sub-categories from a single geography, as well as between each of the sub-categories and the remaining metagenomic datasets (from other geographies) were calculated.

The results depicted in Tables 1A and 1B indicate that metagenomic datasets belonging to the same geography, irrespective of disease status, are separated by a smaller Euclidean distance when compared to the metagenomic datasets from other nationalities.

*Table 3A: Matrix depicting Euclidean distances between metagenomic abundance profiles (based on mean abundance values of a genus across metagenomes) of diabetic and non-diabetic datasets from the Chinese cohort and metagenomic abundance profiles from other nationalities.*

| Metagenomes | Chinese Diabetic | Chinese Non-diabetic | Other nationalities |
|---|---|---|---|
| Chinese Diabetic | - | 0.1018 | 0.1281 |
| Chinese Non-diabetic | 0.1018 | - | 0.1567 |

*Table 3B: Matrix depicting Euclidean distances between metagenomic abundance profiles (based on mean abundance values of a genus across metagenomes) of malnourished (SM), borderline (BL) and healthy (AH) Indian datasets and metagenomic abundance profiles from other nationalities.*

| Metagenomes | AH | BL | SM | Other nationalities |
|---|---|---|---|---|
| AH | - | 0.1436 | 0.2602 | 0.2689 |
| BL | 0.1436 | - | 0.1686 | 0.2696 |
| SM | 0.2602 | 0.1686 | - | 0.3167 |
