# Supplementary material for: Global investigation of composition and interaction networks in gut microbiomes of individuals belonging to diverse geographies and age-groups
Source: Gut Pathog. 2016 May 6;8:17. doi: 10.1186/s13099-016-0099-z (PMC4858888; doi:10.1186/s13099-016-0099-z)
Supplement: Supplementary file 20 — 10.1186/s13099-016-0099-z Short description of the network analysis tool, NAMAP, developed for this study. [file 13099_2016_99_MOESM20_ESM.pdf]

Network Analysis for Metagenomic Abundance Profiles(NAMAP)

For the study carried out (as given in the paper), a tool was developed to create and analyze the networks using metagenomic data. It also has a component which can create abundance profiles (*i.e.* the abundances of different taxonomic groups) of genera from the metagenomic samples.

Metagenomic contigs corresponding to each gut microbiome were taxonomically classified using the approach previously adopted by Ghosh et al. (2014). In this approach, a similarity search of the metagenomic contigs was first performed against a reference database of 2352 bacterial/archaeal genomes [reference 22 of main manuscipt]. Subsequently, the BLASTN output thus obtained was provided as input to the DiScRIBinATE method for obtaining the final taxonomic assignment of the metagenomic contigs (constituting each dataset).

During shotgun sequencing, highly abundant genera are expected to be sequenced with a deeper coverage. Consequently, during contig assembly, (a higher number of) reads from such genera are expected to assemble into longer contigs. In turn, relatively longer contigs will result in longer alignments against database sequences, from an appropriate genus, during a BLAST search. Given this, the taxonomic abundance profile of each gut microbiome was obtained at the genera level using the following formula:

For each genus *i*, its abundance was calculated as -

$$abundance_i = \frac{\frac{M_i}{N}}{database\_proportion_i}$$

where,

M_i is the total number of bases from the contigs (constituting a sample/ dataset) which were aligned to database sequences belonging to the genus 'i'.

N is the total number of bases constituting the contigs present in the metagenomic sample/dataset

and, $database\_proportion_i$ is the proportion of sequences present in the database which belongs to genus 'i'.

Microbial genera that were not identified in at least of the metagenomes under study were identified as sparse and not considered for subsequent analysis. The abundance profiles for gut microbiomes belonging to the different nationalities were then grouped separately and represented as separate abundance matrices (for each nationality).

Abundance based co-occurrence and exclusion networks have been used in many earlier research studies in order to understand the microbial community structures using network analysis [reference 1, 15-17 in main manuscript]. Different types of correlation measures (e.g. Pearson,

Spearman, etc.) have been utilized in these studies to infer co-occurrence and exclusion of bacterial taxonomic groups [reference 15 in main manuscript]. The tool developed for building bacterial interaction networks allows creation of networks from abundance profile using any of the following three methods:

1. Correlation based

Correlation between each pair of micro-organisms was calculated using either Pearson product-moment correlation coefficient or Spearman's ranked correlation coefficient.

$$r_{xy}=\frac{n\sum x_i y_i-\sum x_i\sum y_i}{\sqrt{(n-1)\sum x_{i^2}\left(\sum x_i\right)^2}\sqrt{(n-1)\sum y_{i^2}\left(\sum y_i\right)^2}}$$

2. Expectancy based

In first step correlation between each pair of micro-organisms was calculated. A pair of abundances of samples was swapped for each species at one randomization step. Correlation between each pair of micro-organisms was calculated at each randomization step. Several randomization steps are performed. The expectancy value was calculated using the formula,

$$p-value=\frac{n(p>r)}{N}$$

3. ReBoot method

Sampling of the original abundance data is done. 75% samples are taken randomly and with replacement from the original data. Correlation between each pair of micro-organisms was calculated at each randomization step. Several randomization steps are performed. The mean and standard deviation is for all the correlation coefficients calculated in the randomization steps. These will be the ReBoot values. A parallel run is performed for calculating the expectancy scores. This will serve as the null method. Finally the z-score is calculated using the formula,

$$z=\frac{mean_{reboot}-mean_{null}-g_{avg}}{\sqrt{avg_{std}}}$$

where,

mean(resampling) is the mean of the correlation values in the ReSampling distribution,

mean(null) is the mean of the correlation values in the null distribution,

g(avg) is the global average of all the mean(nulls) obtained for all the pairs of genera and,

avg(std) is computed using the following formula:

$$avg_{std}=\frac{stddev_{reboot}^2\times(N-1)+stddev_{null}^2\times(N-1)}{N-2}$$

stdev(resampling) is the standard deviation of the correlation values in the ReSampling distribution,

stdev(null) is the standard deviation of the correlation values in the null distribution and,

N is the number of randomizations

Construction of edges in the network:

Identify taxa having significant co-occurence or mutual exclusion patterns based on thresholds.

For correlation based,

$$ThresholdR=\frac{t}{\sqrt{n-2+t^2}}$$

where, t - critical t-value, N - number of samples

For expectancy based – threshold p= 0.01 or 0.05

For reboot method – threshold z= 1.65 (at 5%) or 1.96 (at 1%)

Edges were built between genera with significant positive correlations. As a result co-occurrence and mutual exclusion network is formed. Network properties and centrality measures are computed for each node.

Network properties which can be computed are, (i) Number of vertices and edges, (ii) Average Degree, (iii) Diameter (Longest geodesic), (iv) Average shortest path length, (v) Network Density, (vi) Global clustering coefficient (transitivity) and (vii) Network Centralization

Centrality measures which can be computed are, (I) Degree, (ii) Local clustering Coefficient, (iii) Closeness, (iv) Betweenness, (v) Eigen Vector centrality, (vi) PageRank and (vii) Hub Score/Authority Score
